# Small terrestrial mammals of Albania: distribution and diversity (Mammalia, Eulipotyphla, Rodentia)

**DOI:** 10.3897/zookeys.742.22364

**Published:** 2018-03-12

**Authors:** Ferdinand Bego, Enerit Saçdanaku, Michela Pacifici, Carlo Rondinini

**Affiliations:** 1 Department of Biology, Faculty of Natural Sciences, University of Tirana, Albania; 2 Research Centre for Flora and Fauna, Faculty of Natural Sciences, University of Tirana, Albania; 3 Global Mammal Assessment programme, Department of Biology and Biotechnologies “Charles Darwin”, Sapienza University of Rome, Italy

**Keywords:** Balkans, Eulipotyphla, live traps, Natura 2000, rodents

## Abstract

In this paper new records are reported for 23 species of small terrestrial mammals (STM) of Albania collected during the field work campaigns organised in the framework of the project “Strengthening capacity in National Nature Protection – preparation for Natura 2000 network” (NaturAL) in Albania during the summer and autumn of 2016 and 2017 Data on small mammals were primarily collected through Sherman live-trapping campaigns in six high priority protected areas of Albania: Korab-Koritnik, Bredhi i Hotovës, Tomorri, Llogara-Karaburun, Divjakë-Karavasta, Liqeni i Shkodrës (Skadar lake), Lëpushë-Vermosh. Other data were obtained by analysis of owl pellets or by direct observation of individuals (dead or alive) in the field. For 21 species *Erinaceus roumanicus*, *Neomys anomalus*, *Crocidura suaveolens*, *Crocidura leucodon*, *Suncus etruscus*, *Talpa stankovici/caeca*, *Myocastor coypus*, *Sciurus vulgaris*, *Glis glis*, *Dryomys nitedula*, *Muscardinus avellanarius*, *Microtus levis/arvalis*, *Microtus subterraneus*, *Microtus thomasi*, *Microtus felteni*, *Myodes glareolus*, *Apodemus sylvaticus*, *Apodemus flavicollis*, *Apodemus epimelas*, *Mus musculus*, and *Mus macedonicus* additional records are provide and their distributions reviewed, while the presence of two new species of shrews (*Sorex araneus* and *Sorex minutus*) for Albania is reported for the first time. A comprehensive review of the published and unpublished distribution records of STM species of the country is made, together with an updated checklist and distribution maps of the species.

## Introduction

Although the first records of small terrestrial mammals (STM) from Albania date back to Miller (1912), subsequent studies were published only after 1945 and were largely based on very limited records (Puzanov et al. 1955, Rosický and Gjini 1960, Koçi 1961, Bajrami and Serezi 1981, Vangjeli 1987, Anděra 1991, Kryštufek 1994, Prigioni 1996, Macholán and Vohralík 1997, Macholán et al. 2003). More comprehensive studies, published in Albanian language (Bego 1997, 2001, 2003), remained largely unnoticed by mammalogists outside the country. Consequently, by the end of the 20^th^ century, the mammal fauna of Albania was the least known in Europe (Prigioni 1996, Mitchell-Jones et al. 1999), in contrast with other terrestrial vertebrates (amphibians, reptiles and birds) that were better known and recently evaluated (Bego and Koni 1999, Zeneli et al. 2014, Mizsei et al. 2017, Szabolcs et al. 2017).

In 2008, Bego et al. reported a synopsis of the small mammals of Albania (Eulipotyphla, Rodentia), outlining both new records and previously published data. Twenty-four species (seven eulipotyphla and 17 rodents) have been reported for the country. Nine species (*Neomys anomalus*, *Crocidura leucodon*, *Talpa stankovici*, *Dryomys nitedula*, *Muscardinus avellanarius*, *Micromys minutus*, *Mus macedonicus*, *Myodes glareolus*, and *Microtus thomasi*) were recorded in Albania for the first time. Furthermore, a probable presence of a further eleven species was anticipated. Few years later, Paspali et al. (2013a, b) provided supplementary data on small mammals from the southern region of Albania. In 2014, Bego et al. reported the first record of Spalax (Nannospalax) leucodon in Albania. Ciechanowski and Sachanowicz (2014) contributed further knowledge on the distribution and habitat usage of the fat dormouse (*Glis glis*), by providing data on 14 new localities distributed mostly in the northern half of Albania. The number of known small mammal species for Albania has increased recently, with three new species of Cricetids (*Dinaromys bogdanovi, Chionomys nivalis* and *Microtus subterraneus*) recorded by Stolarik et al. (2017) and Stolarik and Jablonski (2017).

The aim of this paper is to provide the most recent records for 23 small mammal species found in protected areas visited within the framework of the “Strengthening capacity in National Nature Protection – preparation for Natura 2000 network” (NaturAL) project, with focus on the new records for the two new shrew species for Albania, and ultimately, to summarise the current distribution of species belonging to the orders Eulipotyphla and Rodentia in the country. Simultaneously, this paper represents the most updated checklist and distribution of the small terrestrial mammals of Albania.

## Materials and methods

**Figure 1.**
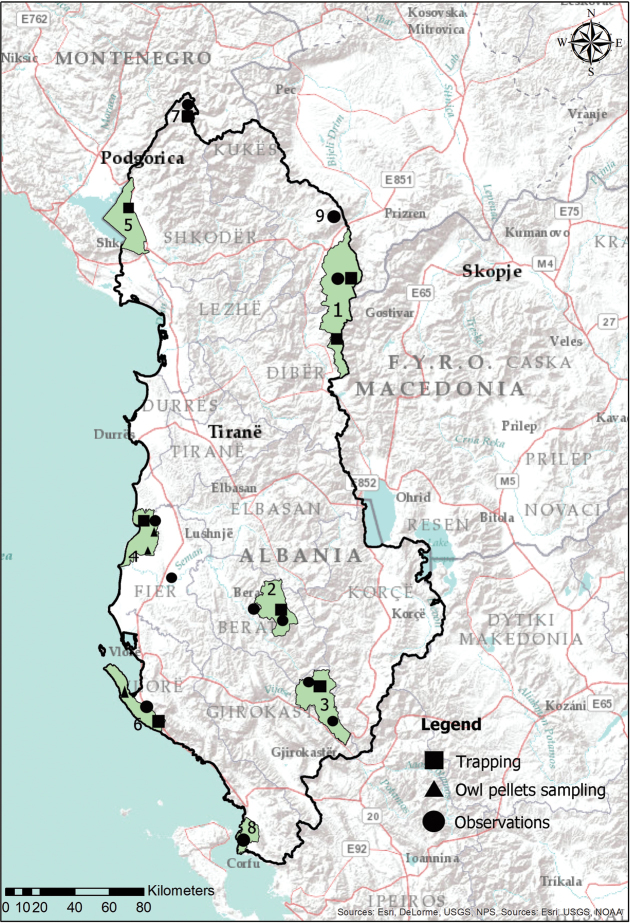
Map of priority Protected Areas of Albania sampled for small mammals: **1** Korab-Koritnik [Novosejë-1, ca. 1400 m a.s.l., Novosejë-2 (Balaj), ca. 1300 m a.s.l.,; Novosejë-3 ca.1350 m a.s.l., Rabdisht (Korab), 1200 m a.s.l, Sllatinë, 1750 m a.s.l.] **2** Tomorr [Ujanik (1440–1500 m a.s.l.) and Peshtan (143 m a.s.l.)] **3** Bredhi i Hotovës [close to Rangers’ Hut (1250 m a.s.l.) and Bënja thermal water springs (368 m a.s.l.)] **4** Divjakë-Karavasta [Divjaka Pine forest (0–2 m a.s.l.), Shën Thanasi monastery (3 m a.s.l.), Shën Kolli monastery (10 m a.s.l.) and Vajkan (10 m a.s.l.)] **5** Liqeni i Shkodrës (lake Skadar) [Breg Liqen (12 m a.s.l.) and Kamice (14 m a.s.l.)] **6** Llogara-Karaburun [Llogara (950 m a.s.l.), Cave of Duk Gjonit (Karaburun, 30 m a.s.l.), and (Dukat, 350 m)] **7** Lëpushë-Vermosh [Lëpushë (1300–1400 m a.s.l.) and Lëpusha stream (ca. 1250 m)] **8** Butrint (Butrint ancient city and lake Bufi, 0-3 m a.s.l.) **9** Pashtrik [Krumë (527 m a.s.l.), Pashtrik (Çaban, ca. 1200 m a.s.l.), and Tej Drinit të Bardhë (ca.1250 m a.s.l.)]

Data on small mammals were primarily collected through Sherman live-trapping campaigns organised in summer and autumn 2016 and 2017 in various high priority protected areas of Albania selected by the NaturAL project: Korab-Koritnik, Bredhi i Hotovës, Mali i Tomorrit, Divjakë-Karavasta, Shkodra lake (Skadar lake), Llogara-Karaburun and Lëpushë-Vermosh. A set of 100 Sherman live traps has been set for 3–4 nights in each of the surveyed protected areas. A mixture of peanut butter and cereals was used as bait. Sometimes, pieces of apple were added inside the trap as additional bait. Captured individuals were handled in plastic bags. After being measured (weight), species and sex determined, including reproduction activity (sexually active or not active, pregnant, lactation etc.), the captured individuals were released. Only animals found dead (very few) inside the traps and few individuals of shrews species recorded for the first time in Albania were taken for collection. Other data were obtained by analysis of owl pellets (20 STM specimens), mostly *Tyto alba* pellets, collected in three sites (two monasteries in Divjakë-Karavasta area, and one in Llogara-Karaburun, Cave of Duk Gjonit), supplemented by direct and indirect (footprints, feeding signs, faeces, burrows, carrions, etc.) observations (28 records) in all surveyed protected areas (Fig. 1). Specimens retrieved from owl pellets were determined at species level, with the help of a Stereomicroscope. The voucher specimens of few individuals found dead inside the traps, including the two new red-toothed shrew species, were morphometrically measured [head and body length (HB), tail (T), hind foot (HF), weight (W)], and sex and sexual activity were determined. Cranial and dentition examinations were conducted for better identification of both red-toothed shrew species. Examined specimens were deposited at the Department of Biology, Tirana University.

The taxonomic identification of our material was based on morphology (Wilson and Reeder 2005). Although such an approach provides reliable results for the great majority of the taxa, in some cases (e.g., the genus *Talpa* and *Microtus arvalis-levis* group) our results should be confirmed through karyological and DNA studies. Therefore, it is likely that in some areas, such as those in the northern part of Albania, the number of reported species may be underestimated.

**Figure 2.**
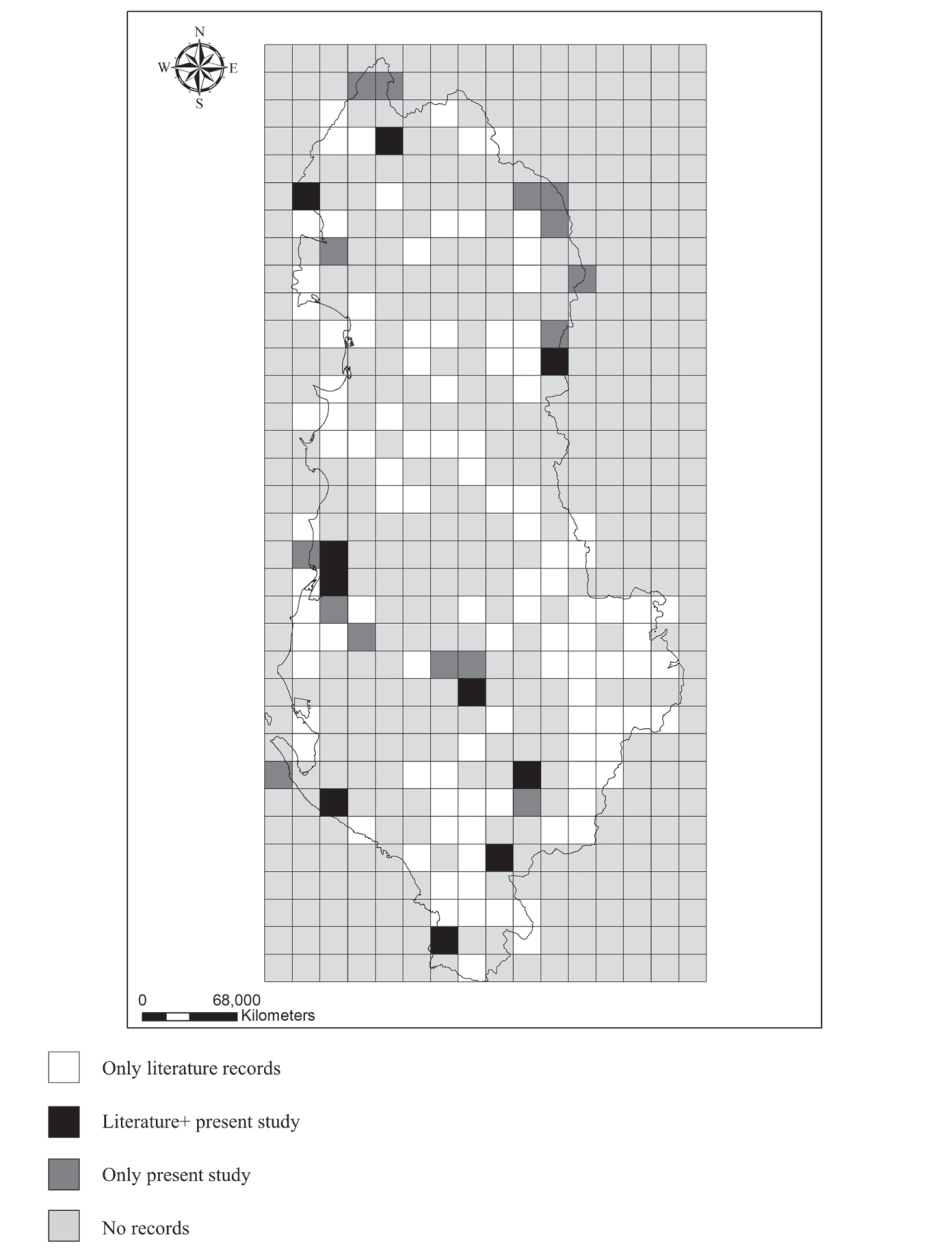
Map of the sources of occurrence records for small terrestrial mammals of Albania (10 × 10 km grid cells).

The current knowledge on small mammal species distribution in Albania, based on published data (Bego et al. 2008, Rogozi et al. 2012, Paspali et al. 2013a, b, Bego et al. 2014, Ciechanowski and Sachanowic 2014, Stolarik and Jablonski 2017, Stolarik et al. 2017) and new records provided in this paper is presented in the Supplementary Information. The distribution maps of the STM species on 10x10 km grid cells were built based on distribution data of published sources and new records collected by this study (Fig. 2). All previous and recent records on STMs are included in the BioNNA database (http://www.bionna.al/) recently established in Albania (Pacifici et al. 2018). Based on previous and recent records a comprehensive checklist of small mammals of Albania was produced as Supplementary Information of this paper. Chorotype and global distribution range of the species are according to IUCN (2017). Distribution records of the species are in WGS84, decimal degrees. ArcGis was used for producing the altitudinal distribution of the records and the distribution maps of the species. Coordinates have been converted to UTM projection to produce the maps. R was used to derive the altitudinal distribution of the species.

Taxonomy and nomenclature follow Wilson and Reeder (2005) and IUCN (2017). Relevant publications (Yalden 1977, Niethammer and Krapp 1978, 1982, 1990, Erfurt 2003) were used to determine the specimen at species level. To distinguish species belonging to the genus *Mus*, reference was made to relevant papers of Macholán (1996) and Kryštufek and Macholán (1998). Key references for small mammals in the neighbouring countries of Albania were Petrov (1992) for former Yugoslavia, Kryštufek and Vohralík (1992) for Montenegro, Kryštufek and Petkovski (2003) for Macedonia and Ondrias (1966), Niethammer (1986), Vohralík and Sofianidou (1987) and Sofianidou and Vohralík (1991) for Greece.

### Study area

Albania is located on the eastern coasts of the Adriatic and Ionian seas. In spite of its small surface area (28,748 km^2^), the country is topographically highly diverse. The Albanian mountains (the highest peak is Mt. Korab, 2753 m a.s.l.), which are physiographically part of Dinarides and Hellenides mountain ranges, rise very steeply from the coastal strip and cover 70 % of the country. As a consequence of Alpine orogenesis, these mountains are characterised by complex folding and faulting, although outcrops of more ancient volcanic and metamorphic rocks are common throughout. The northern mountains, in particular, are singled out by the dominance of carbonate rocks. Thick layers of Late Tertiary or Quaternary sediment cover the 20–30 km wide strip of the coastal lowlands.

Although Albania falls within the temperate zone, topographic heterogeneity and the combination of Mediterranean and continental influences ensure great climatic and vegetation diversity (Reed et al. 2004). The coastal region has a Mediterranean climate with dry, hot summers and mild, wet winters. Further east the mountains act as a barrier to the penetration of warm air masses. The climate of the mountainous regions is Alpine, with strong shifts in precipitation and temperature depending on height and exposure. Differences in temperature between the coast and inland regions are by far most marked in winter, whilst that in precipitation are stronger in summer. Predominant vegetation along the coast is evergreen maquis, which is replaced by oak woodland further inland and finally by beech forests and grasslands at higher altitudes. Forests, which are frequently degraded, cover c. 40 % of the whole surface, while meadows/pastures and arable land cover, respectively, 15 % and 26 % of Albania. Mean human density is 115 inhabitants per km^2^, but the mountainous regions are scarcely populated (Kabo 1990, Bego and Koni 1999, Zeneli et al. 2014).

The main protected areas of Albania are national parks, nature parks, and strict nature reserves. There are 799 protected areas in Albania covering a surface of 460.060.9 ha, or 16 % of the whole national territory. These include two Nature Reserves, 14 National parks, one Marine park, 750 Natural monuments, 22 Habitat/Species Management Areas, nine Protected landscapes, four managed resources areas, one biosphere reserve, three World Heritage sites, and four Ramsar wetlands. The most notable protected areas in Albania are the national parks, having a total area of 210,501.4 ha, or roughly 6.9% of the territory (Zeneli et al. 2014).

Recently, the NaturAL project has initiated the process of establishing the Natura 2000 network and strengthening the national capacity in biodiversity conservation. One of its aims is to improve the knowledge of and experience in biodiversity monitoring in protected areas, by investing in equipment and training the protected areas staff on methods and techniques for studying habitats, and plant and animal species. The NaturAL project is focused on five primary (Liqeni i Shkodrës/Skadar lake, Korab-Koritnik, Divjakë-Karavasta, Tomorr, Bredhi i Hotovës) and 5 secondary (Bunë-Velipojë, Dajti, Shebenik-Jabllanicë, Nartë-Vjosë, Llogara-Karaburun) protected areas, therefore the field campaigns (live trapping, searching for owl pellets and observations of live or dead animals and other species presence signs) were conducted in six priority protected areas and one proposed protected area (Lëpushë-Vermosh), while field observations on small terrestrial mammals were extended to Butrint national park and Pashtrik proposed protected area, situated north of Korab-Koritnik (Fig. 1).

**Figure 3.**
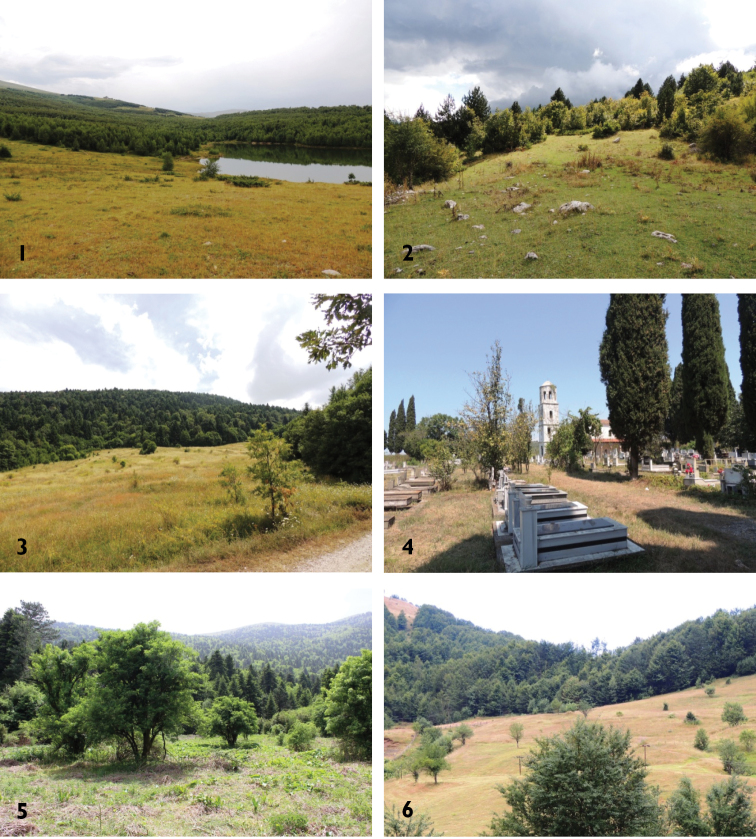
Pictures from the main habitats where searching conducted: **1** Korab-Koritnik, grassland and mixed forest of *Betula pendula* and *Abies alba* **2** Tomorr, open terrain surrounded by mixed forest and *Fagus sylvatica* and *Pinus leucodermis* **3** Bredhi i Hotovës, grassland and mixed forest of *Abies borisi-regis* and *Quercus sp. div.* **4** Divjakë-Karavasta, Shën Kolli monastery, where owl pellets were collected (hedges, arable land and woodland in proximity) **5** Llogara, open terrains and mixed forest dominated by *Abies borisi-regis* and *Carpinus orientalis* on limestone rocks **6** Lëpushë, grassland and open terrains surrounded by beech forest (*Fagus sylvatica*).

Main habitats inside some of the visited protected areas are presented in the Figure 3. Pictures in the Fig. 3 show main sites where live-trapping campaigns were conducted, as well as one of the sites where owl pellets were collected.

## Results

A total of at least 23 small mammal species have been recorded in all surveyed protected areas (Table 1). The highest number of sampled species was reported in Korab-Koritnik (11–14 species), followed by Divjakë-Karavasta (9 species), Lëpushë-Vermosh (7–9 species), Bredhi i Hotovës (7 species), and Llogara-Karaburun (7 species). In Butrint park area no small mammal trapping campaign was organised, but this is the only site where the coypu (*Myocastor coypus*) is established in Albania.

**Table 1.** Records of small terrestrial mammals in the selected protected areas sampled during summer-autumn campaigns in 2016–2017.

Species	Korab-Koritnik-	Tomorr	Bredhi i Hotovës	Divjakë-Karavasta	Liqeni i Shkodrës	Llogara-Karaburun	Lëpushë-Vermosh	Butrint	Pashtrik
*E. roumanicus*		+	+			+			+
*S. araneus*	+								
*S. minutus*	+								
*N. anomalus*							+		
*C. leucodon*				+		+			
*C. suaveolens*			+	+	+	+			
*S. etruscus*				+		+			
*T. stankovici/ caeca/europaea*	+ + +		+	+	+		+ + +		+++
*S. vulgaris*	+	+	+						
*G. glis*			+				+		
*D. nitedula*		+							
*M. avellanarius*				+					
*A. epimelas*						+			
*A. flavicollis*	+		+			+	+		
*A. sylvaticus*	+	+	+	+	+	+	+		
*Mus musculus*				+	+		+		
*Mus macedonicus*	+			+					
*M. arvalis/levis*	+ +								
*M. thomasi*				+					
*M. felteni*	+								
*M. subterraneus*	+								
*Myodes glareolus*	+						+		
*Myocastor coypus*								+	
**Total species**	**11–14**	**4**	**7**	**9**	**4**	**7**	**7–9**	**1**	**2–4**

The uncertain number of species in Korab-Koritnik (11–14), Lëpushë-Vermosh (7–9), and Pashtrik (2–4) is explained by the fact that in this part of Albania we expect a possible sympatry of *Talpa caeca*, *T. stankovici*, and *T. europaea*, and a sympatry of *Microtus arvalis* and *M. levis (mystacinus).*

A full list of the known STM species of Albania (31 species) with their number of records and global distribution range is presented in Table 2. Literature records and new (unpublished) records are presented separately, indicating both the number of localities of species being recorded and number of specimens captured, observed, or retrieved from the owl pellets.

**Table 2.** List of STM species of Albania with number of records and global distribution range.

Nr.	Species name	Number of records				Global Distribution range (according to IUCN 2017)
		Literature records		Unpublished records		
		Number of localities	Numberof specimens	Number of localities	Number of specimens	
1	*Erinaceus roumanicus*	23	23	4	4	Eastern Europe and Asia
2	*Sorex araneus*	–	–	3	15	Palaearctic
3	*Sorex minutus*	–	–	2	6	Palaearctic
4	*Neomys anomalus*	9	99	1	1	Europe and Asia Minor
5	*Crocidura leucodon*	18	827	3	4	Europe and western Asia
6	*Crocidura suaveolens*	38	780	5	19	Palaearctic
7	*Suncus etruscus*	18	423	3	3	Southern Europe and North Africa, through parts of the Near East and Arabian Peninsula, Central Asia, South Asia and mainland Southeast Asia
8	*Talpa caeca*	7	7	5	5	South Europe (Mediterranean)
9	*Talpa stankovici*	7	8	12	12	Balkans endemic
10	*Sciurus vulgaris*	28	28	3	3	Palaearctic
11	*Glis glis*	20	21	3	3	Europe and western Asia
12	*Dryomys nitedula*	2	2	1	1	Palaearctic
13	*Muscardinus avellanarius*	23	63	1	1	Europe and northern Asia Minor (Turkey)
14	*Apodemus sylvaticus*	60	333	9	91	Europe and North Africa
15	*Apodemus flavicollis*	91	214	8	122	Europe, western Asia and Asia Minor
16	*Apodemus epimelas*	13	30	1	7	Endemic to the western and southern Balkans
17	*Mus musculus*	23	43	3	5	Originally a Palaearctic species, but today a Cosmopolite species
18	*Mus macedonicus*	28	1175	3	4	South Balkans, Asia Minor, the Caucasus (Transcaucasia), and the Middle East
19	*Mus spicilegus*	8	102	–	–	Endemic to Europe
20	*Micromys minutus*	7	12	–	–	Palaearctic and Indomalayan regions
21	*Rattus rattus*	31	66	–	–	Originally an Indomalayan species, but it occurs worldwide as an introduced species
22	*Rattus norvegicus*	9	12	–	–	Native to south–east Siberia, north–east China and parts of Japan, but it occurs worldwide as an introduced species
23	*Myodes glareolus*	2	2	2	21	Palaearctic
24	*Microtus levis*	5	20	3	19	East and south–east Europe eastwards across Russia and Asia Minor
25	*Microtus felteni*	11	34	1	1	Balkans endemic
26	*Microtus thomasi*	34	1831	2	4	Endemic to the south–eastern Balkans
27	*Microtus subterraneus*	1	1	2	5	Europe and Asia Minor
28	*Chionomys nivalis*	1	1	–	–	South–western Europe through south–eastern Europe to the Caucasus, Turkey, Israel, Lebanon, Syria, and Iran
29	*Dinaromys bogdanovi*	1	1	–	–	Balkans endemic
30	Spalax (Nanospalax) leucodon	1	1	–	–	Balkan Peninsula and NW Turkey
31	*Myocastor coypus*	–	–	2	5	Native to South America, but introduced to North America, Europe, Africa, and Asia
	**Total**	**203**	**6159**	**33**	**361**	

Our study presents the records for two red-toothed shrew species that are reported for the first time in Albania (*Sorex araneus* and *S. minutus*), and new records for the coypu (*Myocastor coypus*), an introduced species that is already established in Butrint area, southern Albania. Seven STM species were not recorded during our field investigations (*Ch. nivalis*, *D. bogdanovi*, *S. leucodon*, *R. rattus*, *R. norvegicus*, *M. minutus*, and *M. spicilegus*) due to the location of priority protected areas, location and altitude of the sites where live-trapping took place (maximum altitude 1450 m a.s.l.), as well as the limited sampling efforts (in total 33 localities, of which 13 trapping sites, 3 sampling sites for owl pellets and 17 observations for signs of presence of STMs (Table 2).

Although most of the STM species occurring in Albania have a wide global distribution range, being either Palaearctic or Cosmopolite, six species endemic to Balkans (*Talpa stankovici*, *Apodemus epimelas*, *Microtus felteni*, *Microtus thomasi*, *Dinaromys bogdanovi*, and *Spalax leucodon*) and two other species endemic to Europe (*Talpa caeca* and *Mus spicilegus*) have an important part of their global distribution range inside the territory of Albania. However, for most of these endemic species we still have limited data on their distribution and conservation status, except for Thomas’ vole (*Microtus thomasi*), that is found in high density in main lowland agriculture fields and considered as pest species (Bego et al. 2008, Paspali et al. 2013b).

**Figure 4.**
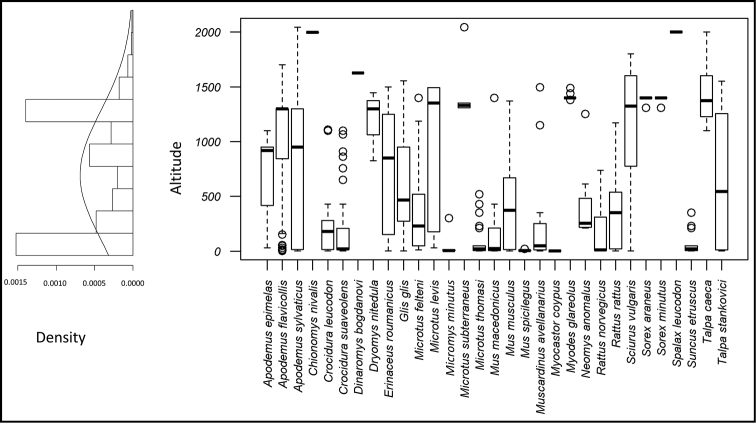
Altitudinal distribution of STM species and frequency of occurrence records by altitude in Albania.

By integrating literature occurrence records with those obtained by this study an altitudinal distribution of STM species and frequency of occurrence records by altitude in Albania was produced and shown in the Figure 4. The density plot indicates that most of the records have been found between 0–200 m a.s.l. and 1200–1400 m a.s.l., that both correspond to altitudes of more frequent sampling efforts (respectively, records retrieved from owl pellets in lower altitudes and records from trapping in higher altitudes). For more information see Suppl. material 2.

The annotated list of 23 STM species reported in this study with synopsis of distributional records (previous and new records) and notes on habitats of species occurrence are described in the following part of the paper.

***Erinaceus roumanicus* Barrett-Hamilton, 1900**

**Previous records. Shkodër**: Velipojë (sea level, June 1992); Thethi (1350 m, June 2001). **Lezhë**: Shengjin (repeated observations). **Durrës**: Rrotull (200 m); Manëz (150 m); Kullë (100 m, July-August 1992). **Tiranë**: Mt. Dajti (1100 m); Vorë (200 m, repeated observations). **Lushnjë**: Divjaka Pine Forest (sea level, repeated observations). **Vlorë**: Nartë (sea level, September 1999), Karaburun-Llogara (10–1200 m, September 1999). **Sarandë**: Butrint (0–150 m, May 1998). **Delvinë**: Syri i kaltër (250 m, May 1998). **Librazhd**: Dardhë (1250 m); Stravaj (1500 m); Rajcë (1350 m); Qarrishtë (1450 m, August 1994). **Pogradec**: Velçan (1050 m, September 1994). **Dibër**: Selishtë (1300 m); Korab (1400 m, June 1999). **Korçë**: Prespa e Madhe, Gollomboç (850 m, April 1995); Dardhë (1450 m, August 1996; May 2002; April 2006); Cangonj (950 m, August 1996; May 2002; April 2006). (Bego et al.2008)

**New records. Krumë**: Has, 2 July 2016 (42.189°N, 20.398°E , 527 m); one individual killed on the road; hedges and arable land in the surrounding. **Tomorr**: Peshtan, 24 Sept 2016 (40.647°N, 20.046°E, 143 m), one individual killed on the road (hedges, shrubs and arable lands nearby). **Bredhi i Hotovës**: 29 Apr 2016, one individual killed on the road (40.028°N, 20.328°E, 280 m). Shrubs and hedges and arable land in the surrounding. **Llogara-Karaburun**: Dukat, 21 Aug 2017 (40.249°N, 19.542°E, 350 m), one individual killed on the road. Shrubland and heaths around.

Four new records are added to the previous ones reported by Bego et al. (2008). All recent records consist of dead animals found on the roads. These records are shown on the map (Fig. Suppl. material 3: S-01). All records (previous and new ones) are spread over the entire Albania, from the sea level to the upper forest line (1500 m). In agreement with hedgehog preferences for lowlands and hills between 300 and 800 m a.s.l. (Mitchell-Jones et al. 1999) in Europe, all our recent records were below 800 m a.s.l (see Fig. 4). As a result of the expanding transport network, the number of road casualties for this species is increasing. Earlier papers refer to this species as *Erinaceus europaeus* (Puzanov and Mitrushi 1955, Koçi 1961) or *E. concolor* (Bego 1997).

***Sorex araneus* (Linnaeus, 1758)**

**New records. Korab-Koritnik**: 1) Novosejë, 25 July 2016; one dead specimen in mixed broadleaved and coniferous forest (41.941°N, 20.576°E): SA-01, HB 62.8 mm, T 42 mm, HF 13.01 mm, W 8.5 g, male, sexual active; 2) Novosejë, 27–28 July 2016 (41.946°N, 20.574°E); 7 ♀ and 5 ♂, trapped in mixed broadleaved and coniferous forests and wet meadows adjacent. Two dead specimens taken for collection (SA-02, HB 61.86 mm, T 42.23 mm, HF 13.07 mm, W 8.0 g, male, sexual active; SA-03, HB 69.86 mm, T 40.29 mm, HF 12.97 mm, W 11.7 g, female, sexual active); 3) Balaj, 29–30 July 2016 (41.960°N, 20.586°E); one ♂ and one ♀ trapped in mixed broadleaved forest and grassland (Suppl. material 3: S02).

**Figure 5.**
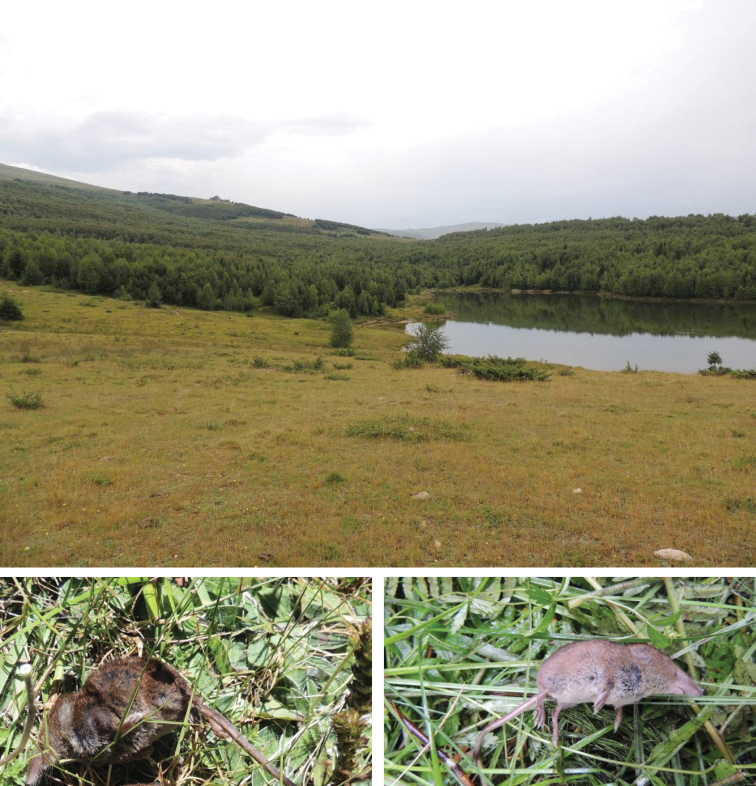
View over the site (**A**) where the two new species of red-toothed shrews [*Sorex araneus* (**B**) and *S. minutus* (**C**)] were recorded. Novosejë, Korab-Koritnik (July 2016).

The common shrew is recorded in Albania for the first time, although its presence was anticipated by Bego et al. (2008). The habitat where most of the specimens were captured represents a mixed broadleaved and fir forest bordering wet meadows (see Fig. 5). The common shrew has a wide distribution in the Palaearctic, and is recorded from sea level to 2,500 m (Anděra 1999), but its distribution in Albania is expected to be limited in forest and pastures of high mountains of northern and north-eastern parts of the country, under the influence of continental climate (Fig. 4).

***Sorex minutus* (Linnaeus, 1766)**

**New records. Korab-Koritnik**: Kukës: Novosejë, 4 ♀ and 2 ♂ trapped in proximity of a water reservoir, ca. 1400 m a.s.l., during 28–29 July 2016, in two localities (41.946°N, 20.573°E; 41.960°N, 20.586°E). Both localities were composed of mixed broadleaved and coniferous forest with adjacent wet meadows. Voucher specimens: SM-01, HB 43.18 mm, T 38.0 mm, HF 10.33 mm, W 3.7 g, male, sexually not active; SM-02, HB 50.69 mm, T 36.75 mm, HF 10.44 mm, W 5.7 g, female, sexually active.

The species was found in sympatry with the common shrew, but in smaller numbers than the latter (Suppl. material 3: S03). It was trapped along the woodland edges confined with swamps and wet meadows in mountain mixed coniferous and broadleaved forests, typical habitats for the species (Hutterer 1990, 1999). Two dead individuals inside the trap were taken for collection. Likewise the common shrew, the presence of pigmy shrew was anticipated by Bego et al. (2008), and this species is added for the first time in the list of the known Albanian terrestrial mammals (see Figure 5).

***Neomys anomalus* (Cabrera, 1907)**

**Previous records**. **Gjirokastër**: Sofratikë, Libohovë, Lazarat, Arshi Lengo, Serat e Mashkullorës, Ura e Kardhiqit: in total 100 specimens from the pellets during 2006–2011 (Bego et al. 2008, Paspali et al. 2013a, b)

**Dibër**: one dead individual along the Drini i Zi river, close to the bridge in proximity of Maqellarë (18 April 2013) (Paspali et al. 2013b)

**New record**. **Lëpushë-Vermosh**: Lëpushë, 29 July 2017 (42.534°N; 19.725°E); one ♀, 12g, found dead inside the Sherman trap (taken for collection), in riparian vegetation along the stream.

The Mediterranean water shrew was reported for the first time in Albania in 2008, with records from the Drinos valley (Bego et al. 2008). Since then it has been regularly found in the owl pellets collected in several localities along the Drinos valley in the southern part of the country (Paspali et al. 2013a, b). On 18 April 2013, Bego found a dead specimen along the Drini i Zi river, close to the bridge in proximity of Maqellarë (Dibër) (Suppl. material 3: S04). The recent record from Lëpusha stream dated 29 July 2017 confirms that this species in the Balkans is more widespread than *N. fodiens* (e.g. Petrov 1992); consequently, its distribution in Albania is expected to be wide, regardless of the small number of records to date (Fig. 4).

***Crocidura leucodon* (Hermann, 1780)**

**Previous records. Durrës**: Rrotull (150 m, August 1992, 1♀). **Tiranë**: Mt. Dajti (1100 m, 1994, 1 ♂). **Lushnjë**: Karavasta, Shën Thanasi Monastery (sea level); Xeng, Shën Kolli Monastery (5 m, between 2001 and 2008, approx. 507 specimens from pellets); Bishqethëm, Shën Mari Monastery (0–5 m, January and April 2008, 60 specimens from pellets). **Fier**: Darzezë, Shën e Premte (sea level, 2002, 38 specimens from pellets); Vajkan, Shën Kolli Monastery (0–20 m, January and April 2008, 2 specimens from pellets). **Ersekë**: Butkë (1108 m, 2007, 1♀). **Gjirokastër**: Bodrishtë (430 m); Lazarat (350 m); Castle (520 m); Libohovë (230 m); Antigone (210 m); **Delvinë**: Mesopotam (50 m); Kalasë (40 m); Vurgu (0–10 m). In total, 370 specimens from pellets from the districts of Gjirokastër and Delvinë during 2004–2011 (Bego et al. 2008, Paspali et al. 2013a, b).

**New records. Llogara-Karaburun**: 25 Jan 2016; Cave of Duk Gjonit, Karaburun (40.290°N; 19.380°E); 2 specimens from the owl pellets; **Divjakë-Karavasta**: 1) Shën Thanasi, 9 Aug 2016; one specimen from the owl pellets (40.877°N, 19.495°E), arable land and hedges; 2) Xeng, Shën Koll, 9 Aug 2016; one specimen from the owl pellets (40.977°N, 19.547°E), arable land and shrubs.

The bicoloured shrew was reported for the first time in Albania in 2008 (Bego et al. 2008). Localities in Albania range from sea level up to 1100 m (Fig. 4). Although the records from the northern part of Albania are missing (Suppl. material 3: S05), it is believed that the bicoloured shrew is widespread in Albania. According to the literature, the bicoloured shrew is common around Skadar Lake in Montenegro (Petrov 1992).

***Crocidura suaveolens* (Pallas, 1811)**

**Previous records. Durrës**: Rrotull (150 m, August 1992). **Tiranë**: Mt. Dajti (1100 m, 1992); **Lushnjë**: Divjaka Pine Forest (sea level, May 1995, 1♀ found dead on a sand dune); Xeng, Shën Kolli Monastery, and Karavasta, Shën Thanasi monastery (2001–2008, 166 specimens from pellets); Bishqethëm, Shën Mari Monastery (0–5 m, January and April 2008, 68 specimens from pellets). **Fier**: Apolloni (April 1998, 2 specimens from pellets); Darzezë, Shën e Premte (sea level, 2002, 21 specimens from pellets); Vajkan, Shën Kolli Monastery (0–20 m, January and April 2008, 7 specimens from pellets). **Vlorë**: Dhërmi (150 m, May 1995, 1 specimen from pellets). **Pukë**: Kryezi (June 2006, 1♂). **Librazhd**: Togëz (233 m, August 2006, 1 ♀ and 1 ♂). **Devoll**: Vranisht (909 m, September 2006, 1 ♀). **Skrapar**: Bogovë (207 m, October 2006, 1 ♀). **Berat**: Drobonik (180 m, October 2006, 1♂). **Tepelenë**: Uji i Ftohtë (176 m, November 2006, 1♂). **Kavajë**: Greth i Vogël (March 2007, 1♀); Spille (sea level, March 2007, 1♀). **Malësia e Madhe**: Kçar i Poshtëm (April 2007, 1♂). **Korçë**: Goricë e Vogël (862 m, September 2007, 1♂). **Ersekë**: Bezhan (1065 m, October 2007, 1♂). **Pogradec**: Gështenjas (756 m, October 2007, 1♀). **Gjirokastër**: Antigone (210 m); Lazarat (350 m); Bodrishtë (430 m); Libohovë (230 m); Sofratikë, Arshi Lengo, Saraqinishtë, Serat e Mashkullorës, Ura e Kardhiqit, Luftinjë, Sukë. Ballaban. **Delvinë**: Dhrovjan, Mesopotam, Shijan. In total 538 specimens from owl pellets in southern Albania during 2004–2011 (Bego et al. 2008, Paspali et al. 2013a, b)

**New records. Llogara-Karaburun**: Cave of Duk Gjonit, Karaburun, 25 Jan 2016 (40.290°N; 19.380°E), one specimen from the owl pellets; shrubs and woodland. **Bredhi i Hotovës**: Thermal water spring, Bënjë, 29 April 2016 (40.243°N; 20.431°E); one specimen found dead on the riverbank, with scattered riparian vegetation. **Divjakë-Karavasta**:1) Shën Thanasi, 9 Aug 2016 (40.877°N; 19.495°E); 3 specimens from owl pellets; arable land and hedges; 2) Xeng, Shën Koll, 9 Aug 2016 (40.977°N; 19.547°E); 2 specimens from owl pellets; arable land and shrubs. **Liqeni i Shkodrës**: Kamicë, 12–15 October 2016 (42.220°N; 19.370°E); 8♀ and 4♂ trapped, measured and 10 released (2 ♀ found dead inside the trap were taken for collection).

The lesser white-toothed shrew is the most widespread shrew in Albania and was found from the sea level up to 1200 m (Fig. 4). Interactions between the two *Crocidura* species are poorly understood. *C. suaveolens* was found on a larger number of localities than *C. leucodon*, however for both species, ca. 50% of records are from lowlands (< 400 m a.s.l.) (Suppl. material 3: S06)

***Suncus etruscus* (Savi, 1822)**

**Previous records. Durrës**: Durrës (from owl pellets; Kahman and Altner, 1956). **Delvinë**: Mesopotam (50 m, 1995, 1996, 2007, 5 specimens). **Lushnjë**: Xeng, Shën Kolli Monastery (5 m) and Karavasta, Shën Thanasi Monastery (sea level; 2000–2008, 275 specimens); Bishqethëm, Shën Mari Monastery (0–5 m, January and April 2008, 29 specimens). **Fier**: Darzezë, Shën e Premte Monastery (sea level, 2002, 35 specimens); Vajkan, Shën Kolli Monastery (0–20 m, January and April 2008, 9 specimens). **Gjirokastër**: Bodrishtë, Sofratikë , Libohovë (230 m), Lazarat (350m), Arshi Lengo, Serat e Mashkullorës, Ura e Kardhiqit, Sukë; in total 64 specimens from owl pellets during 2008–2011 (Bego et al. 2008, Paspali et al. 2013a, b)

**New records. Llogara-Karaburun**: Cave of Duk Gjonit, Karaburun, 25 Jan 2016 (40.290°N; 19.380°E), one specimen from the owl pellets; shrubs and woodland. **Divjakë-Karavasta**: 1) Shën Thanasi, 9 Aug 2016 (40.877°N; 19.495°E); one specimen from the owl pellets; arable land and hedges; 2) Xeng, Shën Koll, 9 Aug 2016 (40.977°N; 19.547°E); one specimen from the owl pellets; arable land and shrubs.

All the previous and recent records on Etruscan shrews (n = 425) were from *T. alba* and other owls’ pellets (Table 2 and Suppl. material 2). Albanian records are all from coastal lowlands, below 400 m a.s.l (Fig. 4 and Suppl. material 3: S07). Given its occurrence in the coast of Montenegro (Petrov 1992), it is doubtlessly present also in northern Albania. In the Balkans this tiny shrew is similarly restricted to the coastal regions below 600 m a.s.l. (Vohralík and Sofianidou 2000) and is common in owl pellets from the north-eastern Adriatic coast (e.g. Lipej and Kryštufek 1991).

***Talpa caeca* Savi, 1822**

**Previous records. Tropojë**: Tropoja (=Tropojë; Kryštufek, 1994); Çerem (1800–2000 m). **Tiranë**: Bizë (1700 m); Mt. Dajti (1100 m). **Librazhd**: Stravaj (1300 m); Dardhë (1700 m); **Skrapar**: Tomorr (Mt. Tomorri, 1700–1900 m). (Bego et al. 2008).

**New records. Krumë**: 1) Pashtrik, Tej Drinit të Bardhë, 2 July 2016 (42.163°N, 20.466°E, 1254 m); small clearings/grasslands in old growth stands of beech forest; mole hills on the ground. 2) Pashtrik, Çaban, 2 July 2016 (42.196°N, 20.475°E, 1203 m); open terrains, grasslands; high activity of moles on the ground. **Lëpushë-Vermosh**: Lëpushë, on 27 and 28 July 2017, in two locations (42.534°N, 19.722°E; 42.534°N, 19.724°E) on relatively wet meadows/grasslands, mole activity on the ground observed. **Korab-Koritnik**: Novosejë, 9 June 2016 (41.947°N, 20.574°E, 1478m); wet meadows, mole hill observed.

***Talpa stankovici* V. Martino & E. Martino, 1931**

**Previous records. Durrës**: Kullë (30 m, repeated observations). **Tiranë**: Vaqarr (150 m, repeated observations); Vorë (200 m, March 2000, 1 ♂). **Lushnjë**: Xeng, Shën Kolli Monastery (5m, 2002 and 2007, 2 specimens from pellets); Divjaka Pine Forest (sea level, January 2008, 1 ♂). **Ersekë**: Boshanj (890 m, October 2007, 1♂). (Bego et al. 2008)

**New records**. **Lëpushë-Vermosh**: Lëpushë, on 27 and 28 July 2017, in two locations (42.534°N, 19.722°E; and 42.534°N, 19.724°E) on relatively wet meadows/grasslands, mole activity on the ground observed. **Krumë**: 1) Pashtrik, Tej Drinit të Bardhë, 2 July 2016 (42.163°N, 20.466°E, 1254m); small clearings/grasslands in old growth stands of beech forest; mole hills on the ground. 2) Pashtrik, Çaban, 2 July 2016 (42.196°N, 20.475°E, 1203 m); open terrains, grasslands; high activity of moles on the ground. **Korab-Koritnik**: 1) Novosejë, 9 June 2016 (41.947°N, 20.574°E, 1478 m); wet meadows, mole hill observed. 2) Novosejë, 25 July 2016 (41.9397°N, 20.571°E, 1550 m), one specimen found dead on the ground. **Bredhi i Hotovës**: 6 Aug 2016 (40.342°N, 20.381°E, 1250 m), grassland; mole activity on the ground observed. **Divjakë-Karavasta**: Divjaka Pine forest. 11 June 2016, in three localities (41.007°N, 19.496°E; 41.008°N, 19.496°E; 41.006°N, 19.496°N), mole activity on the ground observed; Divjaka Pine Forest. 10 Aug 2016, in two localities (41.008°N, 19.496°E; 41.006°N, 19.496°E) mole hills observed.

Three species of moles (*T. europaea, T. caeca*, and *T. stankovici*) are partly sympatric in the countries surrounding Albania (Mitchell-Jones et al. 1999). Although they are well defined by their diploid chromosomal numbers (Soldatović and Dunđerski 1972), cranial differences are slight and vary geographically. Materials from Albania were earlier reported as *T. caeca* (Bego 1997, 2001), but their re-examination revealed also the presence of *T. stankovici* (Kryštufek 1994). The Blind mole is known in Albania from a small number of localities at high altitude (1100–2000 m) (Fig. 4). Characteristic mole hills were also observed in other parts of the country. Particularly, those from the northern alpine pastures (Albanian Alps) very likely belonged to *T. caeca*. However, the presence of *T. europaea* in the Albanian Alps is not excluded (Suppl. material 3: S08).

Records from the coastal area of central Albania are the first evidence connecting the contiguous range of *T. stankovici* in Macedonia and Greece to an isolated occurrence from Ulcinj, Montenegro (Kryštufek 1994). Given its wide altitudinal occurrence in Albania (5–1550 m, Fig. 4) and Macedonia (up to 2200 m; Petrov, 1992), *T. stankovici* is putatively widespread to the south of the River Drin (Bego et al. 2008) (see Suppl. material 3: S09).

***Sciurus vulgaris* Linnaeus, 1758**

**Previous records. Lezhë**: Kune (sea level, repeated observations). **Lushnjë**: Divjaka Pine Forest (sea level). Skrapar: Mt. Tomorri (1600 m). **Tropojë**: Çerem (1700 m, August 1995); Bajram Curri (600 m, August 1995). **Tiranë**: Mt. Dajti (1200 m); Feken (1300 m); Bizë (1600 m), Baldushk (300 m). **Mirditë**: Fan (450 m). **Dibër**: Lurë (1000–1600 m). **Durrës**: Rrotull-Draç-Rodon (100–200 m). **Vlorë**: Llogara (1100 m). **Librazhd**: Dardhë (1400 m); Stravaj (1350 m); Rajcë (1200–1800 m); Qarrishtë (1300–1700 m). **Korçë**: Goricë e madhe (1050m); Moravë (1600 m); Cangonj (950 m); Vithkuq (1400 m). **Ersekë**: Gërmenj-Shelegurë (1200–1800 m). **Përmet**: Bredhi i Hotovës (1300 m). **Gjirokastër**: Bredhi i Sotirës (1300–1800 m); Zheji (1500 m). **Delvinë**: Syri i kaltër (350 m). (Bego et al. 2008)

**New records. Tomorr**: Ujanik, 16 June 2016 (40.630°N, 20.191°E, 1479 m), feeding signs in Coniferous forest, dominated by *Pinus leucodermis*. **Bredhi i Hotovës**: 4 Aug 2016 (40.343 N; 20.379 E, 1150 m), one individual observed close to the rangers’ hut; mixed coniferous and broadleaved forest. **Korab-Koritnik**: Sllatinë, 2 Aug 2017 (41.795°N, 20.460°E, 1750m), feeding signs in Coniferous forest, dominated by *Abies alba* and *Pinus nigra*.

The Eurasian red squirrel is widespread in forested regions throughout Albania, from the sea level to the tree line (1600–1800 m, Fig. 4). Most of our records are based on observations of animals and their characteristic signs (footprints, feeding signs) or vocalisations (Suppl. material 3: S10). Dark colouration was dominant. In 1960–1970 this animal was reported as a game species (Puzanov and Mitrushi 1955, Koçi 1961).

***Glis glis* (Linnaeus, 1766)**

**Previous records. Tiranë**: Mt. Dajti (1100 m, October 1992, 1♀); Qafë Mollë (800 m, August 1996, 1♀). **Lushnjë**: Divjaka Pine Forest (sea level, May 2003, 1♂). **Gjirokastër**: three specimens found in the pellets of *T. alba* and *A. otus* in two locations: Serat e Mashkullorës (1 ind., 2010) and Dhrovjan (2 inds., 2011) (Bego et al. 2008; Paspali et al. 2013b); **Qafa e Malit** (42.090°N, 20.102°E, ~780 m a.s.l.), intensively harvested old beech forest on a steep slope, 11.08.2003, calls heard; **Suç** (41.571°N, 20.054°E, 205 m a.s.l.), a concrete military tunnel with flooded floor, built in vertical rocky outcrops, overgrown by dense shrubs, ivy *Hedera helix* and fig *Ficus carica*, 25.09.2005, one individual observed, calls heard; **Krrabë** (41.208°N, 19.959°E 280 m a.s.l.), a stand of old Platanus trees along a stream, 27.09.2005, calls heard; **Goricë** (41.218°N, 19.959°E, 280 m a.s.l.), a narrow strip of riparian woodland along a river, 18.08.2006, calls heard; **Çiflik** (39.686°N, 20.127°E), an entrance of concrete military tunnel in a slope overgrown by shrubs, 02.05.2010, one individual observed; **Rahovic** (42.426°N, 19.528°E), the valley of the Cemi River, limestone walls with shrubs and sparse trees, near the entrance to the karstic cave, 21.06.2011, calls heard; **Perlat** (41.725°N, 19.983°E, 267 m a.s.l.), a concrete military tunnel in steep, serpentine slope, overgrown with dense shrub (few mature trees nearby), 26.06.2011, few individuals observed climbing on the ceiling; **Lura National Park** (41.715°N, 20.199°E, 1430 m a.s.l.), old beech forest with numerous, hollow trees, 28.06.2011, calls heard, several individuals observed; **Qafa e Shtyllës** (41.376°N, 20.081°E, 1554 m a.s.l.), limestone outcrops overgrown by beech forest, 01.07.2011, calls heard; **Nderlysë** (42.355°N, 19.779°E, 462 m a.s.l.), a concrete bridge over the Shala River, surrounded by a mosaic of low smoke tree *Cotinus coggygria* shrubland and pastures, 10.09.2012, one individual observed; **Prekal** (42.178°N, 19.720°E, 215 m a.s.l.), bare limestone outcrops around a large, karstic cave, 11.09.2012, calls heard; **Kir** (42.212°N, 19.705°E, 298 m a.s.l.), the canyon of the Kiri River, steep slopes with rocky walls and dense shrubland, 11.09.2012, calls heard; **Burrel** (41.593°N, 20.001°E, 287 m a.s.l.), a concrete bunker in a sandstone outcrop, surrounded by mosaic of shrubland and pastures, 13.09.2012, one individual observed; **Nojë** (41.532°N, 19.807°E, 527 m a.s.l.), a concrete tunnel in a treeless, rocky, limestone slope, 14.09.2012, one individual observed (Ciechanowski and Sachanowicz 2014).

**New records. Lëpushë-Vermosh**: Lëpushë, on 28 and 29 July 2017, one female, in lactation was found inside the Sherman trap, in two locations close to each other (42.533°N, 19.720°E); both sites represent a beech forest, harvested some 20–30 years ago. **Shkrel**: Dedaj, 1 Sept 2017, one individual inside a bunker observed (42.293°N, 19.536°E). **Bredhi i Hotovës**: 6 Aug 2016, at the rangers’ hut (40.343°N, 20.379°E), droppings, feeding signs and noise of the fat dormice were recorded. Mixed broadleaved and coniferous forest around.

Following data recorded by Bego et al. (2008), in 2013 Paspali et al. reported three specimens of *Glis glis* from *Tyto alba* pellets in two locations from Drinos valley. Ciechanowski and Sachanowicz (2014) reported 14 new locations distributed throughout Albania, mostly in its northern half (Table 2). Both previous and new records confirm that the fat dormouse is widespread in the forested regions of Albania (cf. Mitchell-Jones et al. 1999), including shrubland or almost barren landscapes with sparse bushes and vertical rocky walls or very steep slopes, man-made habitats (bunkers, tunnels, old mines) or natural (karstic caves) (Fig. 4). *Glis glis* uses a variety of natural and man-made habitats, abandoned military structures, that help in maintaining populations of the species under the heavy ongoing deforestation of Albania (Suppl. material 3: S11). In the 1960s it was considered as a game species (Puzanov and Mitrushi 1955).

***Dryomys nitedula* (Pallas, 1779)**

**Previous records. Librazhd**: Stravaj (1300 m, August 1994, 1 ♀). **Korçë**: Goricë e Vogël (825 m, September 2007, 1 ♀). (Bego et al. 2008)

**New record. Tomorr**: Ujanik, 24 Sept 2016 (40.650°N, 20.190°E, 1446 m); one female trapped in a place of mixed and old growth beech and black pine forest (Suppl. material 3: fig. S12).

Previous and new records of this species are linked with old growing beech forest (Stravaj, Tomorr) and oak woodland (Goricë e Vogël). Albeit rare, the forest dormouse is widespread in the Balkans (Kryštufek and Vohralik 1994); consequently it is likely to be more widespread in the broadleaved forests of Albania (Fig. 4).

***Muscardinus avellanarius* (Linnaeus, 1758)**

**Previous records. Tiranë**: Vorë (250 m, 2004, 1♂). **Lushnjë**: Xeng, Shën Kolli Monastery (2004 and 2007, 2 specimens from pellets); Karavasta, Shën Thanasi Monastery (2006 and 2007, 2 specimens from pellets); Bishqethëm, Shën Mari Monastery (January 2008, 1 specimen from pellets). **Fier**: Darzezë, Shën e Premte Monastery (sea level, 2002, 1 specimen from pellets). **Korçë**: Dardhë (1494 m, October 2007, 1♀). **Delvinë**: Mesopotam (50 m, 15 specimens from pellets); Kalasë (40 m, 2 specimens from pellets) during 2006–2012. **Gjirokastër**: Arshi Lengo, Saraqinishtë. Antigone (210 m), Libohovë (230 m), and Lazarat (350 m), Serat e Mashkullorës, Luftinjë, Ura e Kardhiqit, Sukë; in total 36 specimens from owl pellets during 2006–2012 (Bego et al. 2008, Paspali et al. 2013a, b).

**New records. Divjakë-Karavasta**: Xeng, Shën Koll, 9 Aug 2016 (40.977°N; 19.547°E); one specimen from owl pellets.

Although the common dormouse had already been collected in June 1914 (NMW, specimen from Vermosh, 1150 m), its occurrence in Albania had not been published until 2008 (Bego et al. 2008). Most of the previous and recent records of *M. avellanarius* were retrieved from *Tyto alba* pellets (60 specimens) (Table 2, Suppl. material 2). So far, only two specimens, one in mixed woodland forest on the hills near Vorë (Tiranë), and one in a mixed forest near Dardhë were collected through snap trapping (Bego et al. 2008). *Muscardinus avellanarius* seems to be common in Albania and its altitudinal range extends from the sea level up to 1494 m (Fig. 4), whilst in neighbouring countries (Montenegro, Macedonia), the common dormouse has been found only in mountain forests (Kryštufek and Petkovski 2003). Therefore, we suggest that the common dormouse is probably widespread in Albania (Suppl. material 3: S13).

***Apodemus sylvaticus* (Linnaeus, 1758)**

**Previous records. Durrës**: Rrushkull (sea level, 1992, 2♂ and 1♀). **Tiranë**: Mt. Dajti (1100 m, 1992, 5♂ and 4♀). **Shkodër**: Bogë (1993, altitude 950 m, 1♀); Velipojë (sea level, 1995, 1♂). **Tropojë**: Bajram Curri (450 m and 650 m, 1993, 3♂). **Librazhd**: Dardhë (1700 m, August 1994, 1♂); Stravaj (1400 m, August 1994, 1♂); Qendër (240 m, 2006, 1♂). **Lushnjë**: Xeng, Shën Kolli Monastery (5 m), and Karavasta, Shën Thanasi Monastery (sea level, 2001–2008, c. 75 specimens from pellets); Divjaka Pine Forest (sea level, 1995, 5♂ and 1♀); Bishqethëm, Shën Mari Monastery (0–5 m, January and April 2008, 17 specimens from pellets). **Fier**: Darzezë, Shën e Premte (2002, 3 specimens from pellets). **Skrapar**: Ujanik (1400 m, July 1995, 3♂ and 1♀). **Has**: Myç (386 m, 2006, 1♂). **Pukë**: Kryezi (650 m, 2006, 1♂ and 1♀). **Lezhë**: Gjoshë (74 m, 2006, 1♀); Kune (sea level, 2007, 1♂). **Malësia e Madhe**: Tamarë (293 m, 2006, 1♂); Balçaj (10 m) and Kçar i Poshtëm (15 m, 2007, 5♂ and 3♀). **Dibër**: Brezhdan (580 m, 1♀), Grevë (735 m, 1♀ and 1♂). **Mirditë**: Tarazh (123 m, 2006, 1♀). **Kavajë**: Spille (sea level, 2007, 4♂ and 2♀). **Laç**: Fushë-Kuqe (sea level, 2007, 1♀ and 1♂). **Korçë**: Maliq (Vangjeli, 1987); Plovisht (791 m, 2007, 1♀); Lajthizë (1063 m, 2007, 1♀); Dardhë (1438–1538 m, 2007, 2♀); Gjergjevicë (1009 m, 2007, 1♂). **Ersekë**: Gjonçë (1285 m, 2007, 1♂); Butkë (1108 m, 2007, 4♀); Bezhan (1064 m, 2007, 1♀); Mollas (912 m, 2007, 2♀). **Delvinë**: Pal, Shijan and Mesopotam (50m, 1996, 2004–2012, 31 specimens from pellets). **Gjirokastër**: Humelicë (203 m, 2006, 1♂); Arshi Lengo, Antigone (210 m), Castle (520 m), Lazarat (350 m), Libohovë (230 m), Bodrishtë (430 m), Saraqinishtë, Serat e Mashkullorës, Ura e Kardhiqit (200m), Luftinjë, Suk, Ballaban; in total 118 specimens from pellets during 2006–2012 (Bego et al. 2008, Paspali et al. 2013a, b). **Tomorri mountain**: 23 Sept 2015 (40.621°N, 20.177°E, 2045 m a.s.l.); one individual, female, sexually active captured through live trapping (Stolarik et al. 2017).

**New records**. **Lëpushë-Vermosh**: Lëpushë, 26–29 July 2017; 1♀ and 2♂ sexually active trapped (42.534°N, 19.723°E); beech forest and grassland. **Korab-Koritnik**: Novosejë, 27–29 July 2016; 1♀ and 3♂ trapped (41.945°N, 20.571°E), Mixed broadleaved and coniferous forest. **Liqeni i Shkodrës**: Kamicë, 12–15 Oct 2016; 5♀ and 17♂ trapped (42.222°E, 19.367°E); riparian vegetation and grassland along the lakeshore. **Tomorr**: Ujanik, 23–24 Sept 2016; 2♀ trapped, sexually active (40.649°N; 20.190°E, 1570 m), shrubs and grassland. **Bredhi i Hotovës**: 04–07 Aug 2016; 26 specimens (8♀ and 18♂) trapped (40.340°N; 20.380°E, 1200 m), mixed broadleaved and coniferous forest adjacent to grassland. **Llogara-Karaburun**: 15–17 May 2017; 6♀ and 23♂ trapped (40.213°N; 19.573°E, 950 m); grassland adjacent to mixed woodland. **Divjakë-Karavasta**: 1). Pisha e Divjakës; 9 Aug 2016; one male, sexually active trapped (40.994°N; 19.486°E), coniferous forest; 2) Shën Thanas, 9 Aug 2016; two specimens from the owl pellets (40.877°N; 19.495°E), arable land and hedges; 3) Xeng, Shën Koll, 9 Aug 2016; two specimens from the owl pellets (40.977°N; 19.547°E), shrubs and arable land.

In the Balkans, the taxonomy of field mice from the *Sylvaemus* group (sensu Musser et al. 1996) has not yet been completely agreed upon. A subspecies of this group, *Apodemus sylvaticus stankovici*, described for the Macedonian side of Mt. Korab has been considered to represent a form of *A. flavicollis* (Kryštufek and Stojanovski 1996) or a true species, *A. stankovici* (Petrov 1994). More recently, Bugarski-Stanojević et al. (2008) questioned the presence of *A. sylvaticus* in the Balkans, although the identity of specimens from Greece was ascertained by mitochondrial DNA cytochrome *b* sequences (Michaux and Filippucci 2005). Our identification is based on morphological characteristics that allow the distinction of *A. sylvaticus* and *A. flavicollis* in the western Balkans (Kryštufek and Stojanovski 1996). *Apodemus sylvaticus* is widespread in Albania from the sea level up to 2045 m a.s.l (Stolarik et al. 2017) (Fig. 4 and Suppl. material 3: S14). In many parts of its distribution range in Albania it is found in sympatry with *A. flavicollis* and *A. epimelas* (Bego et al. 2008).

***Apodemus flavicollis* (Melchior, 1834)**

**Previous records. Durrës**: Rrotull (200 m, 1992, 3♀). **Tiranë**: Mt. Dajti (1100 m, 1992, 9♂ and 4♀; 1072 m, 2006, 1♀); Feken (1300 m, 1993, 3♂). **Librazhd**: Dardhë (1700 m, August 1994, 4♂ and 3♀); Qarrishtë (1224 m, 2006, 1♀ and 1♂); Kosharishtë (1356 m, 2006, 1♂); Togëz (233–266, 2006, 2♂); Qendër (248 m, 2006, 3♀ and 1♂); Hotolisht (2007, 2♂ and 1♀). **Skrapar**: Ujanik (1400 m, 1995, 2♂). **Delvinë**: Syri i Kaltër (150 m, 1996, 3♀ and 2♂); Kronjë (2007, 2♀ and 1♂); Mesopotam (50 m, 1996 and 2007, 4 specimens from pellets). **Vlorë**: Dhërmi (150 m, 1995, 1 specimen from pellets); Llogara (822 m, 2006 and 2007, 2♂; 1027 m, 2007, 2♀ and 1♂). **Pukë**: Kryezi (650 m, 2006, 2♂ and 1♀). **Tropojë**: Qafë-Morinë (2006, 1♂). **Dibër**: Brezhdan (580 m, 2006 and 2007, 3♀ and 3♂); Pilaf (606 m, 2006 and 2007, 3♀ and 1♂); Llasen (712 m, 2006 and 2007, 4♀ and 1♂); Ravnë (725 m, 2006, 1♂); Grevë (735 m, 2006, 2♂); Kastriot (650 m, 2007, 1♀). **Përmet**: Mërceq (392 m, 2006, 1♀). **Ersekë**: Gërmenj (973–985 m, 2006, 2♂ and 1♀); Sotiraj (1027 m, 2006, 4♀ and 2♂); Radanj (1212 m, 1♀ and 1♂); Prodanj (1025 m, 2007, 1♂); Kagjinas (1025 m, 2007, 1♀); Leskovik (1003 m, 2007, 1♂); Mollas (912–1009 m, 2007, 3♂ and 2♀); Bezhan (1064 m, 2007, 2♂); Milec (1082 m, 2007, 1♀ and 2♂). **Mat**: Komsi (395 m, 2006, 1♂); German (391 m, 2006, 1♂); Bushkash (152 m, 2006, 1♂); Ulzë (154–160 m, 2006, 2♀ and 1♂). **Krujë**: Cudh-Zalli (729 m, 2006, 1♀); Nojë (697 m, 2006, 1♀). **Korçë**: Voskopojë (1315 m, 2006, 5♀); Voskopojë (1266 m, 2007, 2♂ and 1♀); Gjonomadh (1185 m, 2006, 1♀); Lozhan (716 m, 2007, 2♂ and 1♀); Popçisht (844 m, 2006, 2♂ and 1♀); Senishtë, (762 m, 2006, 1♀); Diellas (1144 m, 2007, 1♂); Goricë e Vogël (862 m, 2007, 1♀); Leshnije (1089 m, 2007, 1♂); Boboshticë, (1352 m, 2007, 1♀); Dardhë (1494 m, 2007, 2♀); Gjergjevicë (1009–1181 m, 2007, 3♀ and 1♂). **Devoll**: Bickë (947 m, 2006, 1♀); Vranisht (909 m, 2006, 1♂); Arrëz (1033–1113 m, 2007, 2♀ and 2♂); Qytezë (1029 m, 2007, 4♀); Çetë (989 m, 2007, 1♂). **Gramsh**: Çekrezë (219 m, 2006, 1♀ and 2♂); Nartë (481 m, 2006, 1♂); Skënderbegas (486 m, 2006, 1♂). **Tepelenë**: Turan (246 m, 2006, 1♀); Salari (584 m, 2006, 1♂). **Lushnjë**: Xeng, Shën Kolli Monastery, and Karavasta, Shën Thanasi Monastery (2001–2008, 6 specimens from pellets); Divjaka Pine Forest (sea level, 2007, 1♀); Bishqethëm, Shën Mari Monastery (January and April 2008, 4 specimens from pellets). **Malësia e Madhe**: Kçar i Poshtëm (15 m, 2007, 1♂); Balçaj (10 m, 2007, 1♀). **Kukës**: Kolesian (750 m, 2007, 2♂); Bicaj (501 m, 2007, 1♂). **Pogradec**: Vërdovë (1151 m, 2007, 2♀); Kalivaç (1131 m, 2007, 1♀); Gështenjas (756 m, 2007, 1♀); Plloçë (813 m, 2007, 1♂); Stropckë (813 m, 2007, 1♀); Dardhas (1188 m, 2007, 1♀); Osnat (1355 m, 2007, 1♀). **Gjirokastër**: Lazarat (350 m, 2006, 1 specimen from pellets); Llongo (2007, 3♀); Koshovicë (2007, 4♀ and 1♂); Sotirë (2007, 1♀ and 2♂); Bodrishtë, Sofratikë, Lazarat, Arshi Lengo, Serat e Mashkullorës, Ura e Kardhiqit, Sukë (20 specimens from owl pellets during 2006–2011). **Delvinë**: Mesopotam (4 specimens from owl pellets during 2006–2011) (Bego et al. 2008, Paspali et al. 2013a, b)

**New records. Lëpushë-Vermosh**: Lëpushë, 27–29 July 2017; 7♀ and 4♂ sexually active trapped (42.53°N, 19.72°E); beech forest. **Korab-Koritnik**: 1) Novosejë, in three different sites (41.946°N, 20.571°E; 41.960°N, 20.586°E; 41.967°N, 20.573°E), during 27–30 July 2016; 6♀ and 9♂ trapped; mixed broadleaved and coniferous forest and *Betula pendula* woodland; 2) Rabdisht, 4 August 2017; 2♂ sexually active, trapped (41.670°N; 20.492°E, 1250 m); beech forest, coppice. **Bredhi i Hotovës**: 04–07 August 2016; 74 specimens (29♀ and 45♂) trapped (40.340°N; 20.380°E, 1200 m), mixed broadleaved and coniferous forest (dominated by *Abies borisi-regis*). **Llogara-Karaburun**: 1) Cave of Duk Gjonit, Karaburun, 25 Jan 2016, one individual retrieved from the owl pellet (40.290°N; 19.380°E), woodland and shrubs; 2) Llogara, 15–17 May 2017; 3♀ and 16♂ trapped (40.213°N; 19.573°E, 950 m), mixed coniferous and broadleaved forest, dominated by *Pinus nigra, Abies-borisi regis* and *Carpinus orientalis*, with *Buxus sempervirens* as understorey.

The yellow-necked field mouse is the most common and widespread small mammal in Albania, except for coastal lowlands, where its habitats have been heavily degraded by agriculture and *A. sylvaticus* predominates (Bego et al. 2008, Paspali et al. 2013a, b). The typical habitat of *A. flavicollis* is forest and woodland: oak forests at 150–860 m a.s.l., beech forests at 1100–1700 m a.s.l., and mixed forests, pine stands and fir forests at intermediate altitudes (730–1350 m) (Fig. 4). Degraded forests, particularly of oak, are as well inhabited as preserved mature stands. Specimens were collected also in hedgerows, orchards, and Alpine meadows just above the tree-line. Most of records (more then 80%) were from areas above 500 m a.s.l.

***Apodemus epimelas* (Nehring, 1902)**

**Previous records. Tiranë**: Mt. Dajti (600 and 1100 m, 1992, 3♀ and 10♂). **Tropojë**: B.Curri (1993, degraded woodland, altitude 350 m, 1♂); **Korçë**: Gollomboç (1995, degraded woodland, altitude 900 m, 1♂); **Vlorë**: Llogara (1996, Pine forest, with very well developed understory, altitude 950 m, 1♀); Llogara (2006, altitude 732 – 919 m, 2♀ and 1♂); Dukat (2006, altitude 415 m, 1♀); Llogara (2007, altitude 822 and 919 m, 1♀ and 1♂); **Delvinë**: Mesopotam (50 m, 1996, degraded woodland, 1 specimen from pellets); Kalasë (30 m, 2006, degraded woodland, 3 specimens from pellets); **Tepelenë**: Zharrë (2006, degraded woodland, 1♀) (Bego et al. 2008, Paspali et al. 2013a, b).

**New records. Llogara-Karaburun**: 16–17 May, 2017; 3♀ and 4♂ trapped (40.213°N; 19.574°E), clearings on rocky limestone area surrounded by mixed coniferous and broadleaved forest, dominated by *Pinus nigra, Abies borisi-regis* and *Carpinus orientalis*, with *Buxus sempervirens* as understorey. It has been found in sympatry with two other *Apodemus* species, *A. flavicollis* and *A. sylvaticus*, but in lower numbers.

Most western broad-toothed field mice were found in degraded woodland on rocky grounds. This is surprising because open rocky areas are its main habitat further north, in former Yugoslavia (Petrov 1992). Records are quite scarce for Albania; nevertheless we suggest this mouse to be widespread on karstic substrates. In Albania this mouse is present between 50 and 1100 m a.s.l. (Fig. 4), but it has been recorded up to 1600 m a.s.l. in neighbouring regions (Petrov 1992).

***Mus musculus* Linnaeus, 1758**

**Previous records. Lushnjë**: Xeng, Shën Kolli Monastery (5m), and Karavasta, Shën Thanasi Monastery (sea level, 2000–2008, 10 specimens from pellets); Bishqethëm, Shën Mari Monastery (2008, 9 specimens from pellets). **Pukë**: Kryezi (650 m, 2006, 1♀). **Kukës**: Nangë (530 m, 2007, 1♂). **Gjirokastër**: Lazarat (479 m, 2007, 2♂); Dunavat (374 m, 2007, 1♀). **Tiranë**: Tirana (September 1960; Macholán et al., 2003); Mt. Dajti (1072 m, 2007, 1♀); Feken (1188 m, 2007, 1♀); Qafë-Mollë (686 m, 2007, 1♀). **Skrapar**: Radësh (2007, 1♂). **Vlorë**: Qeparo (May 1958; Macholán et al., 2003). **Korçë**: Moglicë (844 m, 2007, 1♀; altitude 479 m, 1♂); Popçisht (906 m, 2007, 1♀ and 1♂). **Ersekë**: Qafëzezë (837 m, 2007, 1♂). **Pogradec**: Golik (462 m, 2007, 1♂); Proptisht (527 m, 2007, 1♂); Zamçë (768 m, 2007, 1♀); Vërri (572 m, 2007, 2♀). **Sarandë**: Saranda (May 1958; Macholán et al. 2003). (Bego et al. 2008, Paspali et al. 2013a, b)

**New records. Lëpushë-Vermosh**: Lëpushë, 28 July 2017 (42.533°N, 19.721°E, 1380 m); grassland/ meadow; one female, 17.5 g in lactation trapped. **Liqeni i Shkodrës**: Kamicë, 12 Oct 2016 (42.222°N, 19.369°E), grassland and hedges of riparian vegetation; one male trapped, sexually active; 14 Oct 2016 (42.222°N, 19.369°E), grassland and hedges of riparian vegetation; one female trapped, sexually active. **Divjakë-Karavasta**: Xeng, Shën Koll, 9 Aug 2016 (40.977°N, 19.547°E), 2 specimens from owl pellets.

*Mus musculus* has already been reported in Albania by Vangjeli (1987), Bego (1997), and Bego et al. (2008). Based on previous and recent records (Suppl. material 3: S17), *M. musculus* is probably widespread in rural and sub-rural areas of Albania, as a sinanthropic species, whilst free-living populations mostly belong to *M. macedonicus* (Macholán et al. 2003). Accordingly, in extensive owl-pellet material from the coastal sites of Xeng and Karavasta, collected between 2000 and 2008, we identified only 10 specimens of *M. musculus* (Bego et al. 2008). New record from Lëpusha (July, 2017) raises the distribution range for the commensal house mouse in Albania up to ca. 1400 m a.s.l. (see Fig. 4).

***Mus macedonicus* Petrov & Ruži, 1983**

**Previous records. Lushnjë**: Divjaka pine forest (1995, one male; 2007, one female); Xeng (at Shën Kolli monastery, 10 m a.s.l.) and Karavasta (at Shën Thanasi monastery, 3 m a.s.l.), 350 specimens from pellets during 2000–2008; Bishqethëm (0–5 m, at Shën Mari monastery, January and April 2008, 133 specimens from pellets). **Delvinë**: Mesopotam (50 m, 1996, 2006 and 2007; 45 specimens from pellets); Vurg (0–10 m, 2006, 6 specimens from pellets); Kalasë (40 m, 2006, 4 specimens from pellets). **Fier**: Apolloni (Pojan, altitude 0–30 m, 1998, 3 specimens from pellets); Vajkan, Shën Kolli Monastery (0–20 m, January and April 2008, 19 specimens from pellets). **Gjirokastër**: Bodrishtë (430m), Antigone (210 m), Libohovë (350 m), and Lazarat (350 m), 539 specimens from pellets during 2004–2012 (Bego et al. 2008, Paspali et al. 2013a, b).

**New records. Korab-Koritnik**: 1) Novosejë, close to water reservoir, 28 and 29 July 2016 (41.945°N, 20.571°E), one ♂ and one ♀ trapped on grassland/meadows adjacent to mixed broadleaved and coniferous forest, ca. 1400 m a.s.l. **Divjakë-Karavasta**: Xeng, Shën Koll, 9 Aug 2016 (40.977°N, 19.547°E), 1 specimen from owl pellets; Shën Thanas, 9 Aug 2016 (40.877°N, 19.495°E), 2 specimens from owl pellets.

*Mus macedonicus* is the most common out-door *Mus* species in Albania (Table 2). Most our records are from the coastal lowlands of central and southern Albania (Fig. 4). In the districts of Lushnjë and Fier *M. macedonicus* is evidently sympatric with the morphologically and ecologically similar *M. spicilegus*. Based on owl pellet content the former is by far more abundant (483 vs. 88 specimens in the district of Lushnjë) (Bego et al. 2008). New record from Novosejë (Korab-Koritnik Park) in July, 2016 raises the upper limit of distribution range for the Macedonian mouse in Albania up to ca. 1400 m a.s.l. (see Suppl. material 3: S18).

***Myodes glareolus* (Schreber, 1780)**

**Previous records. Korçë**: Dardhë (1438 m, 2007, 1♀); Voskopojë (1488 m, 2007, 1♂) (Bego et al. 2008).

**New records. Korab-Koritnik**: Novosejë, 26–29 July 2016, some 19 specimens (13♂ and 6♀) were trapped on mixed broadleaved and coniferous forest (41.940°N, 20.570°E, 1460 m). **Lëpushë-Vermosh**: Lëpushë, 27–28 July 2017 (42.532°N; 19.720°E) 1♂, 24.5 g, sexually active and 1♀, 34 g. in lactation were trapped in the same location; beech forest.

*Myodes glareolus* was recorded for the first time in Albania in 2008 by Bego et al. All previous and recent records of this species consisted of high altitude, dense, mixed forest of beach, fir, and pine, with well-developed understory (Fig. 4). With recent records from the northern Albania (Suppl. material 3: S23), the bank vole is expected to be widespread in the mountainous forests of northern and eastern Albania, which putatively represent the southern border of its range (cf. Mitchell-Jones et al. 1999).

***Microtus levis* Miller, 1908**

**Previous records. Delvinë**: Mesopotam (50 m, 1996, 3 specimens from pellets); Kalasë (40 m, 2006, 1 specimen from pellets). **Fier**: Apolloni, Pojan (1998, 14 specimens from pellets). **Gjirokastër**: Gjirokastra (2004, 1 specimen from pellets). (Bego et al. 2008).

**New records. Korab-Koritnik**: 1) Novosejë, close to water reservoir, 28–29 July 2016 (41.945°N, 20.571°E), 5 ♂ and 4 ♀ trapped on grassland/meadows adjacent to mixed broadleaved and coniferous forest; 2) Balaj, 29 July 2016 (41.959°N, 20.586°E), one ♂ trapped on grassland surrounded by mixed broadleaved woodland; 3) Novosejë, close to the new hotel (41.966°N, 20.573°E), 30 July 2016 , 5 ♂ and 4 ♀ trapped inside a woodland of *Betula pendula*.

Considering the ranges of the two sibling *Microtus* species in eastern Macedonia (Petrov 1992) and adjacent Greece (Sofianidou and Vohralík 1991), the materials collected in the southern Albania belong to *M. levis*, while those of northern Albania rather belong to *M. arvalis*. Recent chromosomal diversity in the genus *Microtus* at its southern distributional margin in Iran (Mahmoudi et al. 2014) suggested a new name for *M. levis* as *Microtus mystacinus*, and a possible sympatry of the latter with *Microtus arvalis* in the northern Albania (Suppl. material 3: S24). Karyotype evidence, however, is required to ascertain unambiguously their taxonomic identity. Vangjeli (1987) was the first to report *M. levis* (as *M. epiroticus*) for Albania, specifically for the Korça agricultural area.

***Microtus felteni* Malec & Storch, 1963**

**Previous records. Delvinë**: Mesopotam (50 m, 1996, 2006 and 2007), Kalasë (40 m, 2006), and Vurg (0–10 m, 2006), 22 specimens from pellets. **Gjirokastër**: Castle (520 m), Antigone (210 m), Lazarat (350 m), Bodrishtë (430 m), and Libohovë (230 m), 9 specimens from pellets during 2006–2007) **Pogradec**: Dardhas (1188 m, October 2007, 1♀). **Vlor**ë: Llogara (1050 m, May 1958, 2 specimens; Anděra 1991). (Bego et al. 2008).

**New records. Korab-Koritnik**: Novosejë, 27 July 2016 (41.945°N, 20.571°E, 1485m), one specimen (male) trapped in a small clearing of a mixed broadleaved and coniferous forest, 100 m uphill from the water reservoir (Suppl. material 3: S25).

The Balkan pine vole is a rare and little known Balkan endemic. It has been observed in only 13 localities (Kryštufek and Petkovski 2003), but its potential range might cover an area of ca. 40,000 km^2^ in Albania, Serbia, Kosovo, Macedonia, and Greece (Shenbrot and Krassnov 2005). The first record of the species for Albania was reported by Anděra (1991). Our collection related to this species includes at least 31 specimens found in pellets, a pregnant female (with six embryos) captured in a small clearing inside mixed deciduous woodland in Pogradeci district (Bego et al. 2008), and recent record in Novosejë (27 July 2016), a male, sexually active, trapped again in a small clearing inside a mixed deciduous and coniferous woodland.

***Microtus thomasi* (Barrett-Hamilton, 1903)**

**Previous records. Delvinë**: Mesopotam (50 m, 1996, 2006 and 2007, 111 specimens from pellets); Kalasë (40 m, 2006, 51 specimens from pellets); Vurg (0–10 m, 2006, 5 specimens from owl pellets); Konispol (25 specimens trapped). **Fier**: Apolloni, Pojan (0–30 m, 1998, 42 specimens from pellets); Darzezë, Shën e Premte Monastery (close to sea level, 2002, 26 specimens from pellets); Vajkan, Shën Koll Monastery (0–20 m, 2008, 292 specimens from pellets). **Lushnjë**: Xeng, Shën Kolli Monastery, and Karavasta, Shën Thanasi Monastery (2001–2008, 188 specimens from pellets); Bishqethëm, Shën Mari Monastery (0–5 m, January and April 2008, 85 specimens from pellets). **Vlorë**: Dukat (40 specimens trapped). **Përmet**: Tre Urat (10 specimens trapped). **Gjirokastër**: Gjirokastra Castle (520 m), Antigone (210 m), Arshi Lengo, Sofratikë, Krinë, Saraqinishtë, Serat e Mashkullorës, Ura e Kardhiqit, Luftinjë, Sukë, Ballaban; in total 1099 specimens from owl pellets during 2004–2012 (Bego et al. 2008, Paspali et al. 2013a, b).

**New records**. **Divjakë-Karavasta**: Xeng, Shën Koll (40.977°N, 19.547°E), 3 specimens from owl pellets (9 Aug 2016). **Fier**: Vajkan (40.772°N, 19.624°E), high activity of voles on the ground, alfalfa crop fields (9 March 2017; Suppl. material 3: S26).

In Albania, Thomas’ pine vole is the most common vole in the diet of *Tyto alba* and our collection includes 1835 specimens, of which 1760 retrieved from owl pellets and 75 specimens from live trapping. Although all our records are from the coastal lowlands, we assume that Thomas’ pine vole is probably more widespread at low and medium altitudes, between 0–600 m (cf. Shenbrot and Krassnov 2005). In some parts of Myzeqe Field it is causing damages to perennial crops and is considered as a pest.

***Microtus subterraneus* (de Selys-Longchamps, 1836)**

**Literature records. Tomorri mountain**: 23 Sept 2015 (40.621°N, 20.177°E, 2045 m above sea level); one individual, female, sexually active captured through live trapping (Stolarik et al. 2017).

**New records. Korab-Koritnik**: 1) Novosejë (Balaj), 29 July 2016 (41.960°N, 20.586°E), three ♂ trapped on meadow/grassland surrounded by mixed broadleaved woodland; 2) Novosejë, close to the new hotel (41.967°N, 20.573°E), 30 July 2016, two ♂ trapped in *Betula pendula* forest (one individual taken for collection: MS-01, HB 103.7 mm, T 40.48 mm, HF 15.29 mm, W 27.3 g, female, sexually active).

*Microtus subterraneus* occurs primarily in Europe, where it occupies central regions from the Atlantic coast of France to European Russia, and the Balkan peninsula (Shenbrot and Krasnov 2005). There are records of this species from sea level to 2,300 m (Kryštufek 1999). *Microtus subterraneus* is known in Kosovo, Macedonia, and Montenegro (Kryštufek and Petkovski 2003). This species was reported for the first time in Albania by Stolarik et al. (2017) in the Tomorri Mountain, where an individual of *M. subterraneus* was trapped in a grassland situated at the altitude of 2045 m a.s.l. on 23 Sept 2015. The new records of this species from Novoseja-Shishtaveci area, part of the Korab-Koritnik park, indicates that this vole species is probably more widespread in northern and eastern Albania.

***Myocastor coypus* (Molina, 1782)**

**Figure 6.**
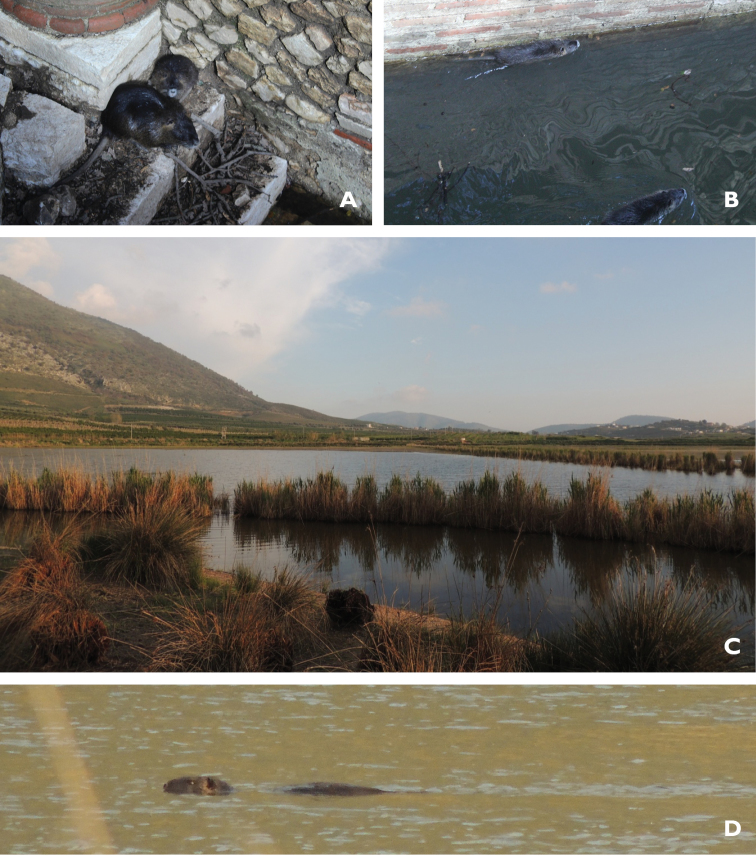
Pictures of *Myocastor coypus* from Butrint National park: **A** Adult and subadult at the bank side of a small pool inside the ancient town of Butrint **B** Two other individuals swimming in the same pool **C** View of the southern edge of Bufi lake where an adult of coypu swimming at shallow water swamp, adjacent to south edge of Bufi lake was observed (**D**).

**New records. Butrint**: 1) Butrint ancient city (39.745°N, 20.020°E); two adults and two juveniles observed on 02 March 2013; 2) Bufi lake (39.742°N, 20.065°E); one individual swimming, at the southern edge of shallow waters of Bufi lake, April 2014 (see Fig. 6).

This species is native to South America, and has been introduced to North America, Europe, Africa, and Asia. It is patchily distributed throughout its historic range in association with mesic habitats and water bodies. It is increasing in number, and is considered as a pest in parts of its range and has been the subject of eradication measures (Bertolino et al. 2005). This species has been introduced and is present in the surrounding countries (Montenegro, Kosovo, Macedonia, and Greece; Sofianidou and Vohralík 1991, Kryštufek and Petkovski 2003). In Albania it has been introduced in the early 70’s of the last century in the Patoku hunting reserve, but since then there has not been any evidence of its presence in the area. In recent years, the species has been reported in Butrint National Park and Ramsar site, where the species is well established (Suppl. material 3: S31).

## Discussion

The high species diversity and endemism for mammals in the Balkans is indicated for the mountainous regions of the southern Dinarides and the Shara-Pindos Mountains, which encompass Albania (Kryštufek and Griffiths 2002, Kryštufek 2004). Albania shares the distribution range of six Balkan endemic STM species (*Talpa stankovici, Apodemus epimelas, Microtus felteni, M. thomasi, Dinaromys bogdanovi* and *Spalax leucodon*); therefore, Albania is expected to play an important role in the conservation of these endemics (Bego et al. 2008, Kryštufek et al. 2009).

The new and previous records reported and analysed in this paper confirmed the species diversity of this part of the Balkans: 31 STM species are already reported in Albania, but the presence of another five species is anticipated (*Sorex alpinus*, *Neomys fodiens*, *Talpa europaea*, *Microtus arvalis*, *Arvicola amphibius*), due to the existence of these species in the countries surrounding Albania (Petrov 1992, Kryštufek and Vohralík 1992, Kryštufek and Petkovski 2003, Niethammer 1986, Vohralík and Sofianidou 1987, Sofianidou and Vohralík 1991).

A notable progress in studying STM species of Albania was made over the recent years (see Table 2 and Suppl. material 2). Thus, Bego et al. (2008) reported nine species (*Neomys anomalus, Crocidura leucodon, Talpa stankovici, Dryomys nitedula, Muscardinus avellanarius, Micromys minutus, Mus macedonicus, Myodes glareolus* and *Microtus thomasi*) new for the country, raising the number of known STM species to 24 (Table 2 and Suppl. material 2). Paspali et al. (2013a, b) presented abundant records for 14 small mammal species from 23 localities in the southern region of Albania, mainly through analysis of owl pellets. In 2014, Bego et al. reported the first record of *Spalax leucodon* in Dhëmbel Mt (Përmet), while recently three species of voles (*Dinaromys bogdanovi, Microtus subterraneus* and *Chionomys nivalis*) were recorded for the first time in Albania by Storalik et al. (2017) and Storalik and Jablonski (2017), respectively *D. bogdanovi* in proximity of Thethi National Park (42.390°N, 19.726°E; 1626 m), and *M. subterraneus* and *C. nivalis* in Tomorri Mt (40.627°N, 20.173°E, 1998 m). In Tomorri Mt. the upper limit of distribution range of *Apodemus sylvaticus* in Albania was extended from 1700 m to 2045 m a.s.l. (Storalik et al. 2017) (Fig. 4).

The list of shrews in Albania is amended with two red-toothed shrew species (*Sorex araneus* and *S. minutus*) whose first records of their presence in the country come from Novosejë-Shishtavec area, Kukës district, part of Korab-Koritnik Nature Park. Considering habitat requirements of these species and their distribution records in the neighbouring countries, we believe that both species are likely present in other parts of northern Albania (Anděra1999, Hutterer 1990, 1999, Petrov 1992, Kryštufek and Vohralík 1992, Kryštufek and Petkovski 2003). In this paper we also report additional records of *M. subterraneus* and *M. felteni* in north-eastern Albania (Novosejë-Shishtavec, Kukës district, as part of Korab-Koritnik Nature Park).

All four recently reported rodent species (*D. bogdanovi, M. subterraneus, C. nivalis*, and *S. leucodon*) were recorded in the mountainous habitats of northern, north-eastern and south-eastern Albania. In spite of the very low number of specimens captured to date, their distribution range in Albania is expected to be wider (Ondrias 1966, Kryštufek 1999, Kryštufek et al. 2008, Kryštufek and Bužan 2008, Kryštufek and Amori 2017, Petrov 1992, Shenbrot and Krasnov 2005, Vohralík and Sofianidou 1987, Sofianidou and Vohralík 1991). Therefore, future field investigatioon should focus on these regions, so that to verify and confirm their distribution and conservation status in Albania.

Our sampling campaigns and field investigation in priority Protected Areas of Albania during 2016 and 2017 enriched our knowledge for several other small terrestrial mammals that are still poorly known, such as *Suncus etruscus*, *Neomys anomalus, Dryomys nitedula*, *Muscardinus avellanarius, Apodemus epimelas, Microtus subterraneus, Microtus felteni*, and *Myodes glareolus*. In spite of the progress, the knowledge on distribution range of dormice, especially for *D. nitedula* and *M. avellanarius*, remains poor in Albania, due to lack of proper methods and equipment to study them; the records for these two dormouse species come from live-trapping and owl pellets, while dormouse nest boxes, the most effective method, has not been practiced yet. However, the use of the recently purchased dormouse nest boxes by the trained local staff of Protected Areas under the NaturAL Project will bring more data on the dormice species in Albania in a near future.

The presence of the coypu, *Myocastor coypus*, an introduced rodent species to Europe but native to South America (Bertolino et al. 2005, IUCN 2017), is for the first time confirmed in the southern Albania, both in the Butrint ancient city and Bufi lake, part of Butrint National park area. The species has been repeatedly reported over the last four years (2013–2017) by rangers of Butrint National Park, including the evidence of reproduction, and this is a good indication that *M. coypus* is well established in the Butrint Park area. The possible interactions and impacts of this introduced species on native fauna of the Butrint area should be studied and monitored.

The sampling efforts done over recent years in Albania have not yet produced the evidence of presence for the water vole (*Arvicola amphibius*), alpine shrew (*Sorex alpinus*), and water shrew (*Neomys fodiens*) in the country. The water vole is recorded around two lakes shared between Albania and Montenegro (Lake Shkodra) and Macedonia (Lake Ohrid; Petrov 1992), but it was not found in the Albanian part of Shkodra (Skadar) Lake. Habitat degradation and high seasonal fluctuations of water level in Shkodra Lake might be the reasons for the absence of the species in the Albanian part of the lake. In any case, *T. europaea* and *M. arvalis* are presumably present in the mountains north of the River Drin (Yigit et al. 2016, Amori et al. 2017). However, further sampling efforts should be made to confirm this assumption.

The taxonomic identification of our material was based on morphology. Although such an approach provides reliable results for the great majority of the taxa listed above, in some cases (e.g., the genus *Talpa* and *Microtus arvalis-levis* group) our results need to be strengthened by karyological and DNA studies. Chromosomal variability is perhaps not indicative of cryptic speciation, however it reveals the phylogeographic structuring of a small-range endemism (Zima J. 2004, Mahmoudi et al. 2014). The Balkan Peninsula was one of the major refugia from European glaciation over the last two million years of climatic oscillations. Unsurprisingly, given the high topographic diversity of the region, phylogeographic studies based on molecular markers have revealed many microrefugia within the major refugium (e.g. Kryštufek et al. 2007). The role played by Albania as a refugium for small terrestrial mammals is still mostly unknown and should be further studied.

Finally, it must be underlined that there are major conservation issues associated with Albanian small terrestrial mammals. The number of extinctions strongly correlates with the number of endemisms, and the species which display both restricted ranges and low densities suffer the highest risk of extinction (Nott and Pimm 1997). All mammalian species endemic to the Balkans should receive the greatest attention with respect to conservation and research. Natura 2000 as an important instrument of nature conservation for EU member states can offer little support to mammalian endemic species of the Balkans. The inclusion of sites in the N2000 network is related to the fact that occurring species are included in the EU Habitats Directive, but Balkan endemic species often very important for conservation are still not present in the HD lists simply because they live in non-EU countries. Throughout the Balkans, and Albania in particular, the reality of conservation issues sharply contrasts with species requirements. The recent decline of the Mt. Galičica population of *D. bogdanovi*, a western Balkans endemism, is representative of a wider situation (Kryštufek and Bužan 2008). The low numbers of specimens for endemic and rare species such as *D. bogdanovi*, *M. felteni*, *C. nivalis*, and absence of other species such as *Sorex alpinus, Neomys fodiens, A. amphibius*, might be a strong signal of worsening conditions for these species due to anthropogenic stressors, including habitat degradation and destruction and climate change.

## Conclusion

Knowledge on small mammals of Albania has been significantly improved over the last two decades, due to contributions from local and foreign researchers. The number of known species has increased (at present 31 species, of which nine species of Eulipotyphla and 22 species of Rodents), almost reaching the potential number of STM species in the country (36 species) according to literature review of species distribution in the countries around Albania (Bego et al. 2008). However, in spite of the progress made, the presence and distribution range of five other species (*Talpa europaea, Sorex alpinus*, *Neomys fodiens, Arvicola amphibius, Microtus arvalis*) in Albania remain to be verified in the coming years. Sampling efforts should be focused on the central and northern parts of Albania, where most of these species co-occur. Subalpine and alpine areas of these parts of Albania should be sampled more intensively, with the use of the proper sampling techniques and supplemented with karyological and DNA analysis (Zima 2004, Mahmoudi et al. 2014, Storalik et al. 2017, Storalik and Jablonski 2017) to better distinguish potential cryptic taxa.

## Supplementary materials

Supplementary material 1 Updated check-list of small terrestrial mammals of Albania (2017) Data type: species data This is an open access article distributed under the terms of the Creative Commons Attribution License (CC BY 4.0), which permits unrestricted use, distribution, and reproduction in any medium, provided the original author and source are credited.Ferdinand Bego, Enerit Saçdanaku, Michela Pacifici, Carlo Rondinini

Supplementary material 2 Table S2. List of species with numbers of records by year of publication and/or year of data collection Data type: species data This is an open access article distributed under the terms of the Creative Commons Attribution License (CC BY 4.0), which permits unrestricted use, distribution, and reproduction in any medium, provided the original author and source are credited.Ferdinand Bego, Enerit Saçdanaku, Michela Pacifici, Carlo Rondinini

Supplementary material 3 Link: https://doi.org/10.3897/zookeys.742.22364.suppl3 Data type: occurence Explanation note: Distribution maps of small terrestrial mammals of Albania (Fig. S01. *Erinaceus roumanicus*; Fig. S02. *Sorex araneus*; Fig. S03. *Sorex minutus*; Fig. S04. *Neomys anomalus*; Fig. S05. *Crocidura leucodon*; Fig. S06. *Crocidura suaveolens*; Fig. S07. *Suncus etruscus*; Fig. upplementary materia. *Talpa caeca*; Fig. S09. *Talpa stankovici*; Fig. S10. *Sciurus vulgaris*; Fig. S11. *Glis glis*; Fig. S12. *Dryomys nitedula*; Fig. S13. *Muscardinus avellanarius*; Fig. S14. *Apodemus sylvaticus*; Fig. S15. *Apodemus flavicollis*; Fig. S16. *Apodemus epimelas*; Fig. upplementary materia. *Mus musculus*; Fig. S18. *Mus macedonicus*; Fig. S19. *Mus spicilegus*; Fig. S20. *Micromys minutus*; Fig. S21. *Rattus rattus*; Fig. S22. *Rattus norvegicus*; Fig. S23. *Myodes glareolus*; Fig. S24. *Microtus levis*; Fig. S25. *Microtus felteni*; Fig. S26. *Microtus thomasi*; Fig. S27. *Microtus subterraneus*; Fig. S28. *Chionomys nivalis*; Fig. S29. *Dinaromys bogdanovi*; Fig. S30. Spalax (Nanospalax) leucodon; Fig. S31. *Myocastor coypus*). This is an open access article distributed under the terms of the Creative Commons Attribution License (CC BY 4.0), which permits unrestricted use, distribution, and reproduction in any medium, provided the original author and source are credited.Ferdinand Bego, Enerit Saçdanaku, Michela Pacifici, Carlo Rondinini
