# Supplementary material for: Small terrestrial mammals of Albania: distribution and diversity (Mammalia, Eulipotyphla, Rodentia)
Source: Zookeys. 2018 Mar 12;(742):127–63. doi: 10.3897/zookeys.742.22364 (PMC5904422; doi:10.3897/zookeys.742.22364)
Supplement: Supplementary material 1 — Updated check-list of small terrestrial mammals of Albania (2017) [file zookeys-742-127-s001.docx]

**Supplement 1**

**Updated check-list of small terrestrial mammals of Albania (2017)**

**Order Eulipotyphla**

Fam. Erinaceidae

1. *Erinaceus roumanicus* (Barrett-Hamilton, 1900)

Fam. Soricidae

1. *Sorex araneus* (Linnaeus, 1758)
2. *Sorex minutus* (Linnaeus, 1766)
3. *Neomys anomalus* (Cabrera, 1907)
4. *Crocidura leucodon* (Hermann, 1780)
5. *Crocidura suaveolens* (Pallas, 1811)
6. *Suncus etruscus* (Savi, 1822)

Fam. Talpidae

1. *Talpa caeca* (Savi, 1822)
2. *Talpa stankovici* (V. et E.Martino, 1931)

**Order Rodentia**

Fam. Sciuridae

1. *Sciurus vulgaris* (Linnaeus, 1758)

Fam. Gliridae

1. *Glis glis* (Linnaeus, 1766)
2. *Dryomys nitedula* (Pallas, 1779)
3. *Muscardinus avellanarius* (Linnaeus, 1758)

Fam. Muridae

1. *Apodemus sylvaticus* (Linnaeus, 1758)
2. *Apodemus flavicollis* (Melchior, 1834)
3. *Apodemus epimelas* (Nehring, 1902)
4. *Mus musculus* (Linnaeus, 1758)
5. *Mus macedonicus* (Petrov and Ružić , 1983)
6. *Mus spicilegus* (Petenyi, 1882)
7. *Micromys minutus* (Pallas, 1771)
8. *Rattus rattus* (Linnaeus, 1758)
9. *Rattus norvegicus* (Berkenhout, 1769)

Fam. Cricetidae

1. *Myodes glareolus* (Schreber, 1780)
2. *Microtus levis* (Miller, 1908)
3. *Microtus felteni* (Malec and Storch, 1963)
4. *Microtus thomasi* (Barrett-Hamilton, 1903)
5. *Microtus subterraneus* (de Selys-Longchamps, 1836)
6. *Chionomys nivalis* (Martins, 1842)
7. *Dinaromys bogdanovi* (V. et E. Martino, 1922)

Fam. Spalacidae

1. *Spalax* (*Nannospalax*) *leucodon* (Nordmann, 1840)

Fam. Myocastoridae

1. *Myocastor coypus* (Molina, 1782)
