# Supplementary material for: Small terrestrial mammals of Albania: distribution and diversity (Mammalia, Eulipotyphla, Rodentia)
Source: Zookeys. 2018 Mar 12;(742):127–63. doi: 10.3897/zookeys.742.22364 (PMC5904422; doi:10.3897/zookeys.742.22364)
Supplement: Supplementary material 2 — Table S2. List of species with numbers of records by year of publication and/or year of data collection [file zookeys-742-127-s002.docx]

**Supplement 2**

**Table S2. List of species with numbers of records by year of publication and/or year of data collection**

| Species name | Number of records by year of publication (for published sources) and year of data collection (for unpublished data indicated with *) | | | | | | | | | | | | | | | | | | | | | | | | | | |
| --- | --- | --- | --- | --- | --- | --- | --- | --- | --- | --- | --- | --- | --- | --- | --- | --- | --- | --- | --- | --- | --- | --- | --- | --- | --- | --- | --- |
|  | 1912 | 1955 | 1960 | 1981 | | 1987 | | 1991 | | 1994 | | 1997 | | 2003 | | 2008 | | 2013 | | 2014 | | 2017 | | 2013-16* | | 2017* | |
|  | Localities or specimens | Localities or specimens | Localities or specimens | Localities | Specimens | Localities | Specimens | Localities | Specimens | Localities | Specimens | Localities | Specimens | Localities | Specimens | Localities | Specimens | Localities | Specimens | Localities | Specimens | Localities | Specimens | Localities | Specimens | Localities | Specimens |
| *E. roumanicus* |  | 1 | 1 |  |  |  |  |  |  |  |  |  |  |  |  | 23 | 23 |  |  |  |  |  |  | 3 | 3 | 1 | 1 |
| *S. araneus* |  |  |  |  |  |  |  |  |  |  |  |  |  |  |  |  |  |  |  |  |  |  |  | 3 | 15 |  |  |
| *S. minutus* |  |  |  |  |  |  |  |  |  |  |  |  |  |  |  |  |  |  |  |  |  |  |  | 2 | 6 |  |  |
| *N. anomalus* |  |  |  |  |  |  |  |  |  |  |  |  |  |  |  | 2 | 6 | 7 | 93 |  |  |  |  |  |  | 1 | 1 |
| *C. leucodon* |  |  |  |  |  |  |  |  |  |  |  |  |  |  |  | 8 | 610 | 10 | 217 |  |  |  |  | 3 | 4 |  |  |
| *C. suaveolens* |  | 1 |  |  |  |  |  |  |  |  |  |  |  |  |  | 22 | 281 | 15 | 498 |  |  |  |  | 5 | 19 |  |  |
| *S. etruscus* |  | 1 |  |  |  |  |  |  |  |  |  |  |  |  |  | 8 | 358 | 9 | 64 |  |  |  |  | 3 | 3 |  |  |
| *T. caeca* |  |  |  |  |  |  |  |  |  | 1 | 1 |  |  |  |  | 6 | 6 |  |  |  |  |  |  | 3 | 3 | 2 | 2 |
| *T. stankovici* |  |  |  |  |  |  |  |  |  |  |  |  |  |  |  | 6 | 7 | 1 | 1 |  |  |  |  | 10 | 10 | 2 | 2 |
| *S. vulgaris* |  | 1 | 1 |  |  |  |  |  |  |  |  |  |  |  |  | 26 | 26 |  |  |  |  |  |  | 2 | 2 | 1 | 1 |
| *G. glis* |  | 1 |  |  |  |  |  |  |  |  |  |  |  |  |  | 3 | 3 | 2 | 3 | 14 | 14 |  |  | 1 | 1 | 2 | 2 |
| *D. nitedula* |  |  |  |  |  |  |  |  |  |  |  |  |  |  |  | 2 | 2 |  |  |  |  |  |  | 1 | 1 |  |  |
| *M. avellanarius* |  |  |  |  |  |  |  |  |  |  |  |  |  |  |  | 12 | 28 | 10 | 34 |  |  |  |  | 1 | 1 |  |  |
| *A. sylvaticus* |  |  | 1 |  |  | 1 | 1 |  |  |  |  |  |  |  |  | 44 | 215 | 13 | 115 |  |  | 1 | 1 | 7 | 59 | 2 | 32 |
| *A. flavicollis* |  |  | 1 |  |  | 1 | 1 |  |  |  |  |  |  |  |  | 81 | 195 | 8 | 17 |  |  |  |  | 5 | 90 | 3 | 32 |
| *A. epimelas* | 1 |  | 1 |  |  |  |  |  |  |  |  |  |  |  |  | 10 | 27 | 1 | 1 |  |  |  |  |  |  | 1 | 7 |
| *M. musculus* |  |  |  |  |  | 1 | 1 |  |  |  |  | 1 | 1 | 3 | 3 | 18 | 38 |  |  |  |  |  |  | 2 | 4 | 1 | 1 |
| *M. macedonicus* |  |  |  |  |  |  |  |  |  |  |  |  |  |  |  | 13 | 636 | 15 | 539 |  |  |  |  | 3 | 4 |  |  |
| *M. spicilegus* | 1 |  |  |  |  |  |  |  |  |  |  | 1 | 1 |  |  | 6 | 100 |  |  |  |  |  |  |  |  |  |  |
| *M. minutus* |  |  |  |  |  |  |  |  |  |  |  |  |  |  |  | 6 | 11 | 1 | 1 |  |  |  |  |  |  |  |  |
| *R. rattus* |  |  | 1 | 1 | 3 | 1 | 1 |  |  |  |  |  |  |  |  | 21 | 46 | 7 | 15 |  |  |  |  |  |  |  |  |
| *R. norvegicus* |  |  | 1 |  |  | 1 | 1 |  |  |  |  |  |  |  |  | 7 | 10 |  |  |  |  |  |  |  |  |  |  |
| *M. glareolus* |  |  |  |  |  |  |  |  |  |  |  |  |  |  |  | 2 | 2 |  |  |  |  |  |  | 1 | 19 | 1 | 2 |
| *M. levis* |  |  |  |  |  | 1 | 1 |  |  |  |  |  |  |  |  | 4 | 19 |  |  |  |  |  |  | 3 | 19 |  |  |
| *M. felteni* |  |  |  |  |  |  |  | 1 | 2 |  |  |  |  |  |  | 10 | 32 |  |  |  |  |  |  | 1 | 1 |  |  |
| *M. thomasi* |  |  |  |  |  |  |  |  |  |  |  |  |  |  |  | 14 | 930 | 20 | 901 |  |  |  |  | 1 | 3 | 1 | 1 |
| *M. subterraneus* |  |  |  |  |  |  |  |  |  |  |  |  |  |  |  |  |  |  |  |  |  | 1 | 1 | 2 | 5 |  |  |
| *C. nivalis* |  |  |  |  |  |  |  |  |  |  |  |  |  |  |  |  |  |  |  |  |  | 1 | 1 |  |  |  |  |
| *D. bogdanovi* |  |  |  |  |  |  |  |  |  |  |  |  |  |  |  |  |  |  |  |  |  | 1 | 1 |  |  |  |  |
| *S. leucodon* |  |  |  |  |  |  |  |  |  |  |  |  |  |  |  |  |  |  |  | 1 | 1 |  |  |  |  |  |  |
| *M. coypus* |  |  |  |  |  |  |  |  |  |  |  |  |  |  |  |  |  |  |  |  |  |  |  | 2* | 5* |  |  |
| **Total** | **2** | **5** | **7** | 1 | **3** | 6 | **6** | 1 | **2** | 1 | **1** | 2 | **2** | 3 | **3** | 161 | **3611** | 23 | **2499** | 15 | **15** | 4 | **4** | 26 | **277** | 7 | **84** |
