# Supplementary material for: Small terrestrial mammals of Albania: distribution and diversity (Mammalia, Eulipotyphla, Rodentia)
Source: Zookeys. 2018 Mar 12;(742):127–63. doi: 10.3897/zookeys.742.22364 (PMC5904422; doi:10.3897/zookeys.742.22364)

**Supplement 3**

**Figures S01 – S31.** Distribution maps of small terrestrial mammals of Albania (Fig.S01. *Erinaceus roumanicus*; Fig. S02. *Sorex araneus*; Fig. S03. *Sorex minutus*; Fig. S04. *Neomys anomalus*; Fig. S05. *Crocidura leucodon*; Fig. S06. *Crocidura suaveolens*; Fig.S07. *Suncus etruscus*; Fig. S08. *Talpa caeca*; Fig. S09. *Talpa stankovici*; Fig. S10. *Sciurus vulgaris*; Fig. S11. *Glis glis*; Fig. S12. *Dryomys nitedula*; Fig. S13. *Muscardinus avellanarius*; Fig. S14. *Apodemus sylvaticus*; Fig. S15. *Apodemus flavicollis*; Fig. S16. *Apodemus epimelas*; Fig. S17. *Mus musculus*; Fig. S18. *Mus macedonicus*; Fig. S19. *Mus spicilegus*; Fig. S20. *Micromys minutus*; Fig. S21. *Rattus rattus*; Fig. S22. *Rattus norvegicus*; Fig.S23. *Myodes glareolus*; Fig. S24. *Microtus levis*; Fig. S25. *Microtus felteni*; Fig. S26. *Microtus thomasi*; Fig. S27. *Microtus subterraneus*; Fig. S28. *Chionomys nivalis*; Fig. S29. *Dinaromys bogdanovi*; Fig. S30. *Spalax (Nanospalax) leucodon*; Fig. S31. *Myocastor coypus*)


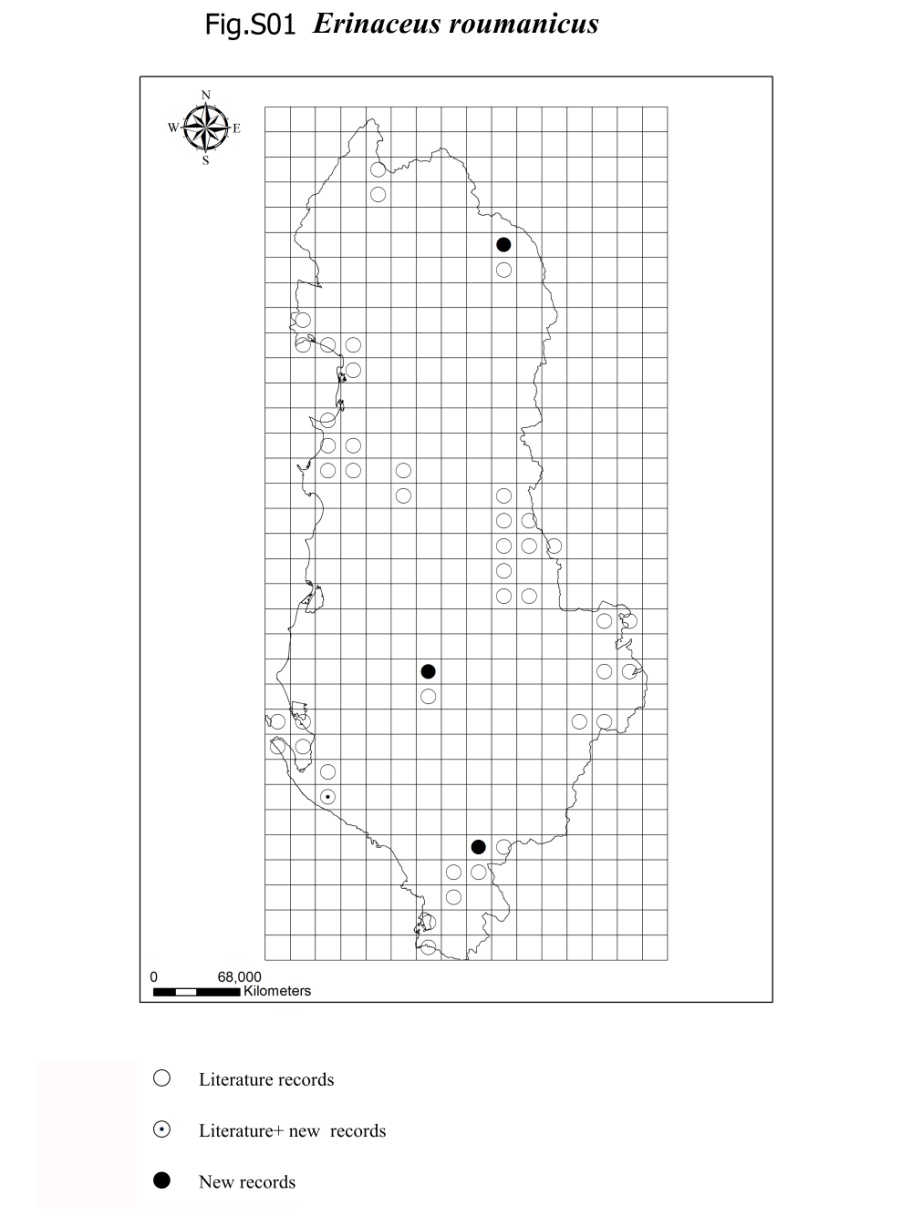

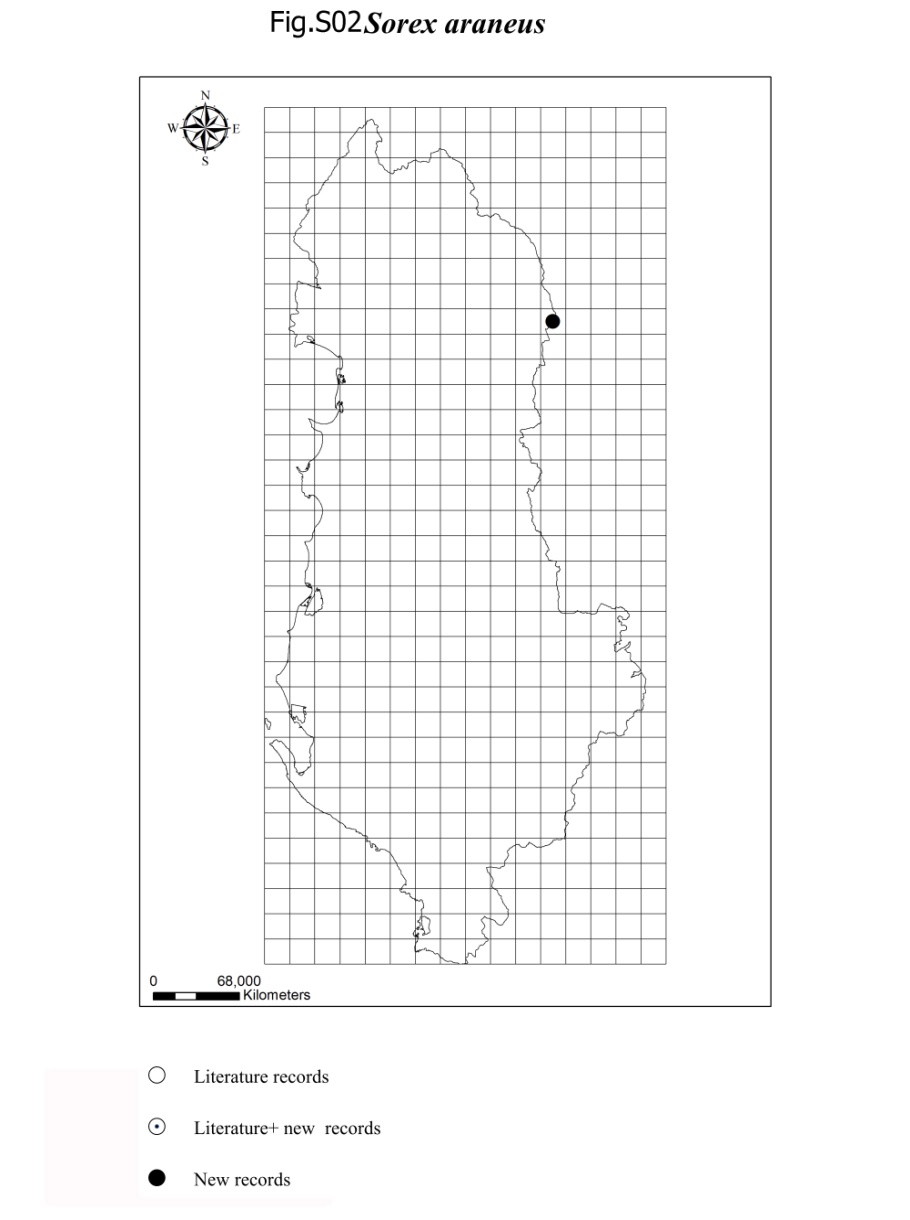

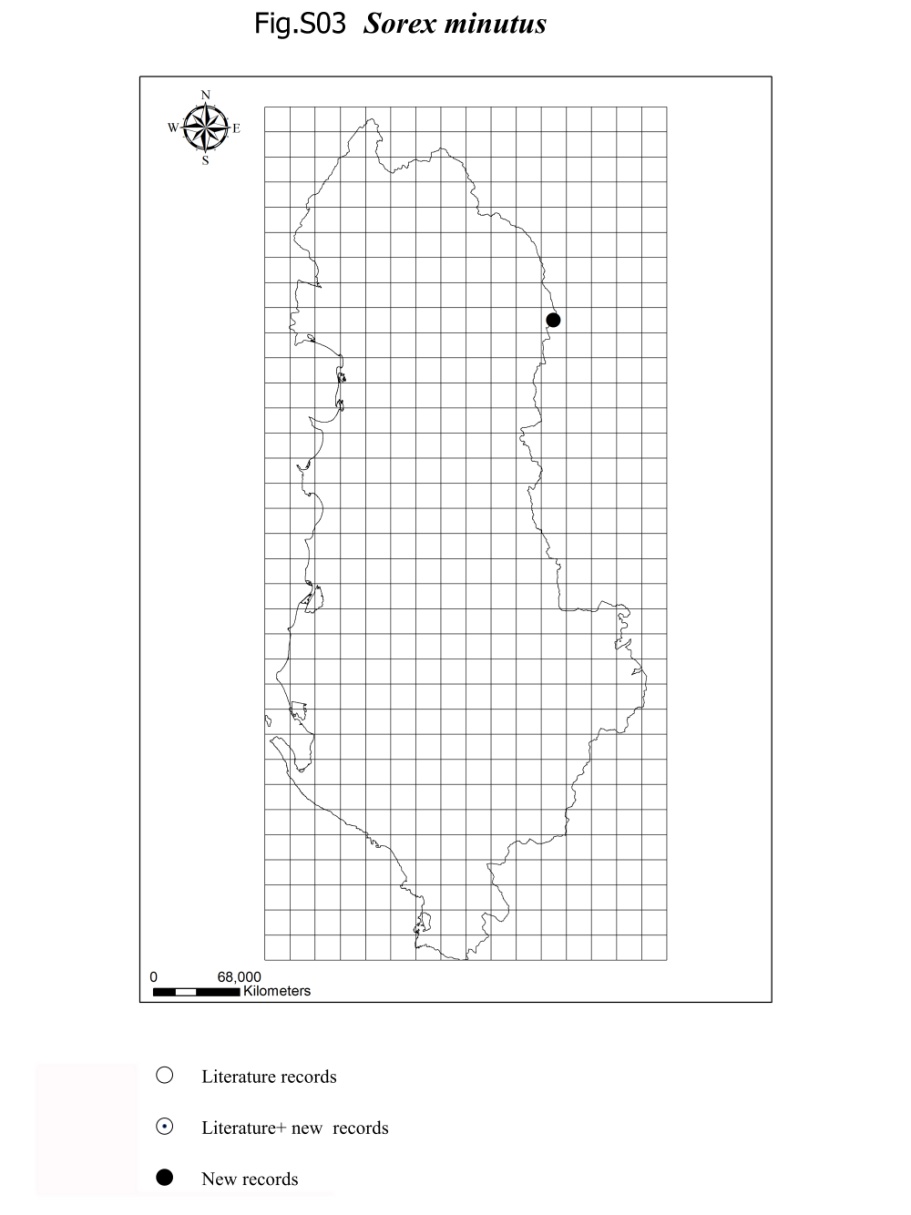


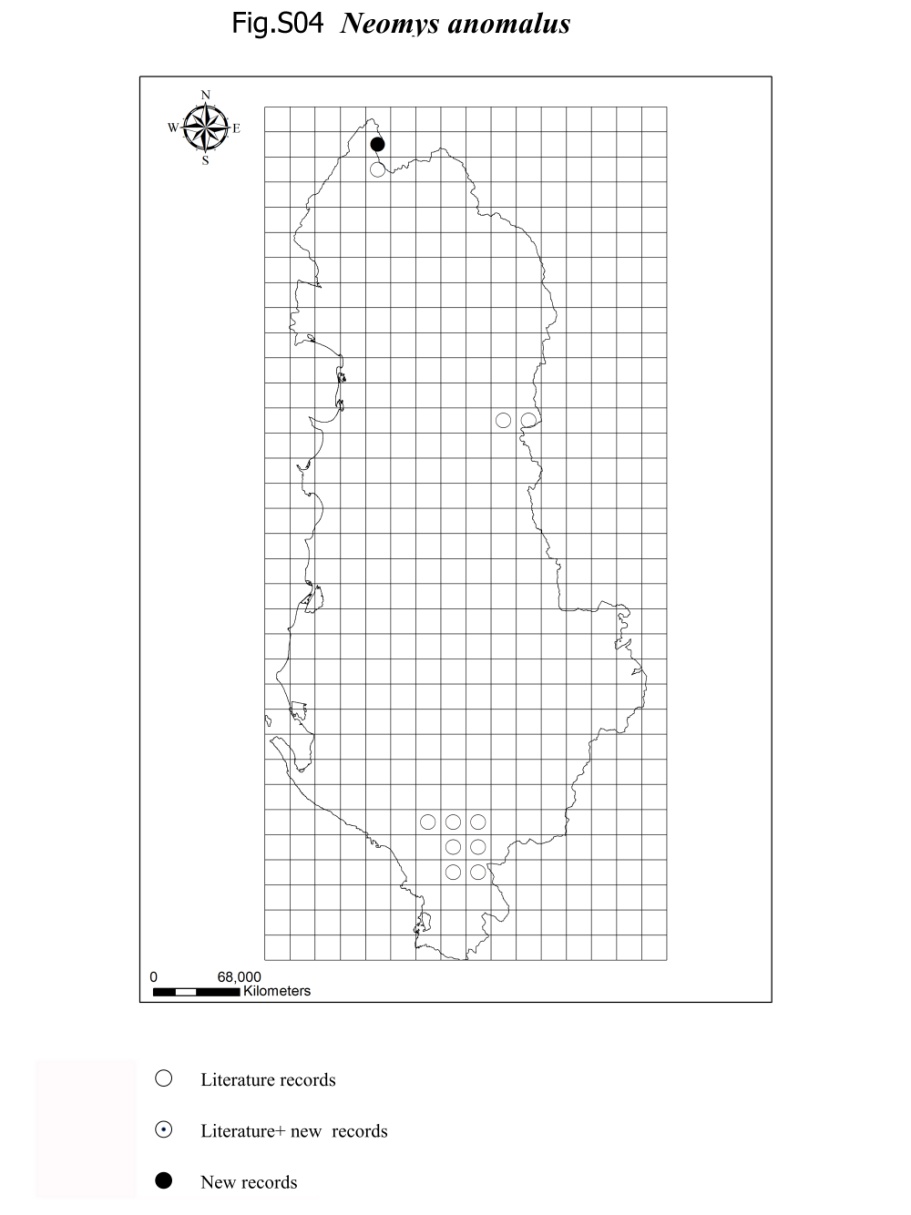

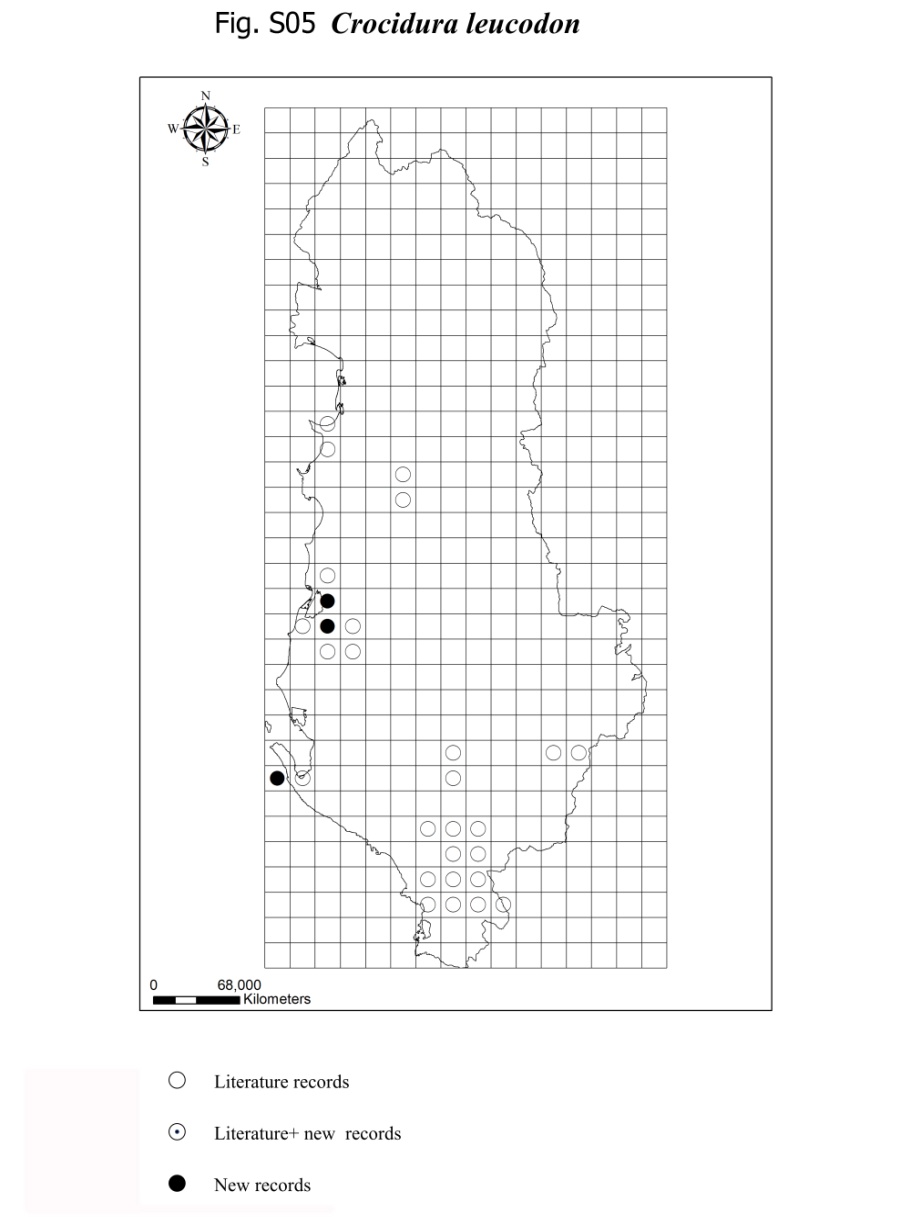

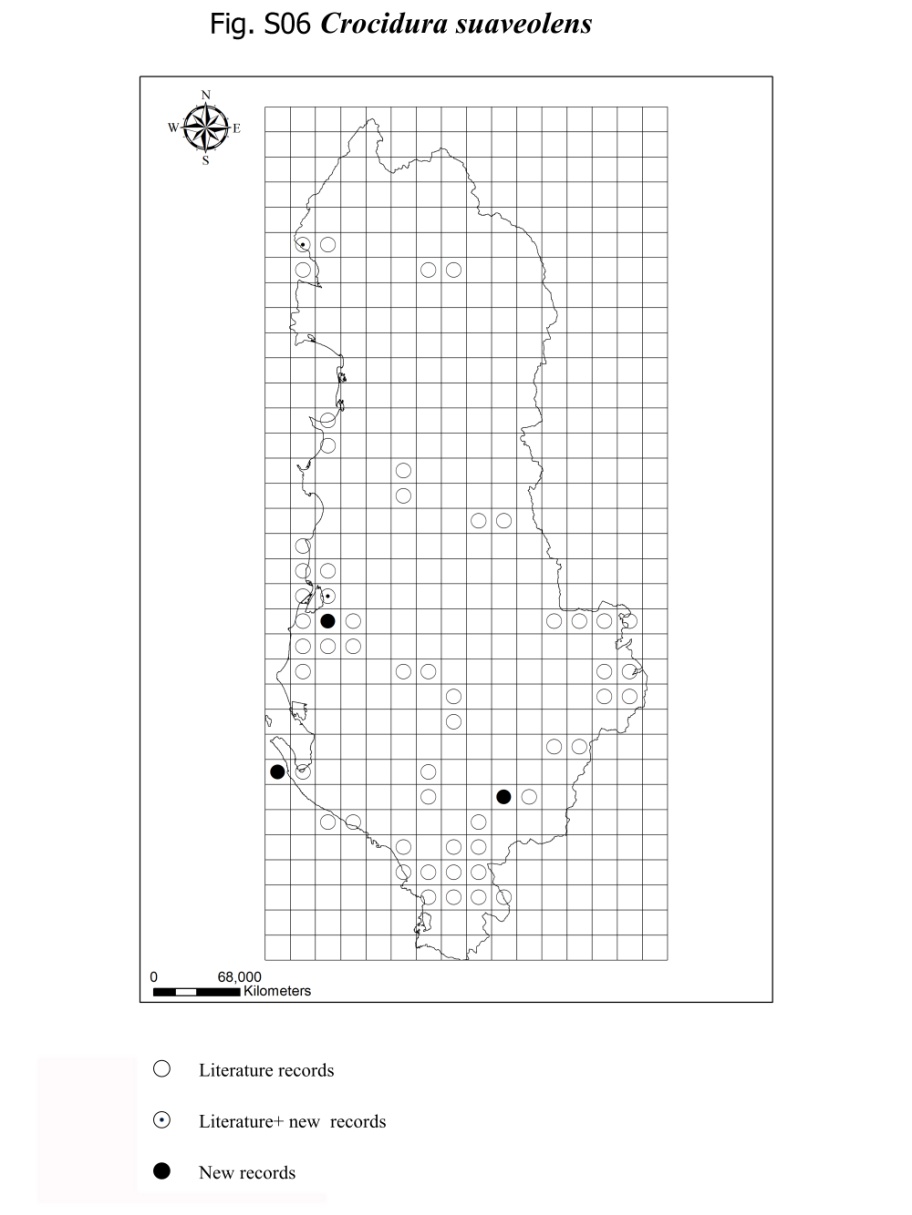


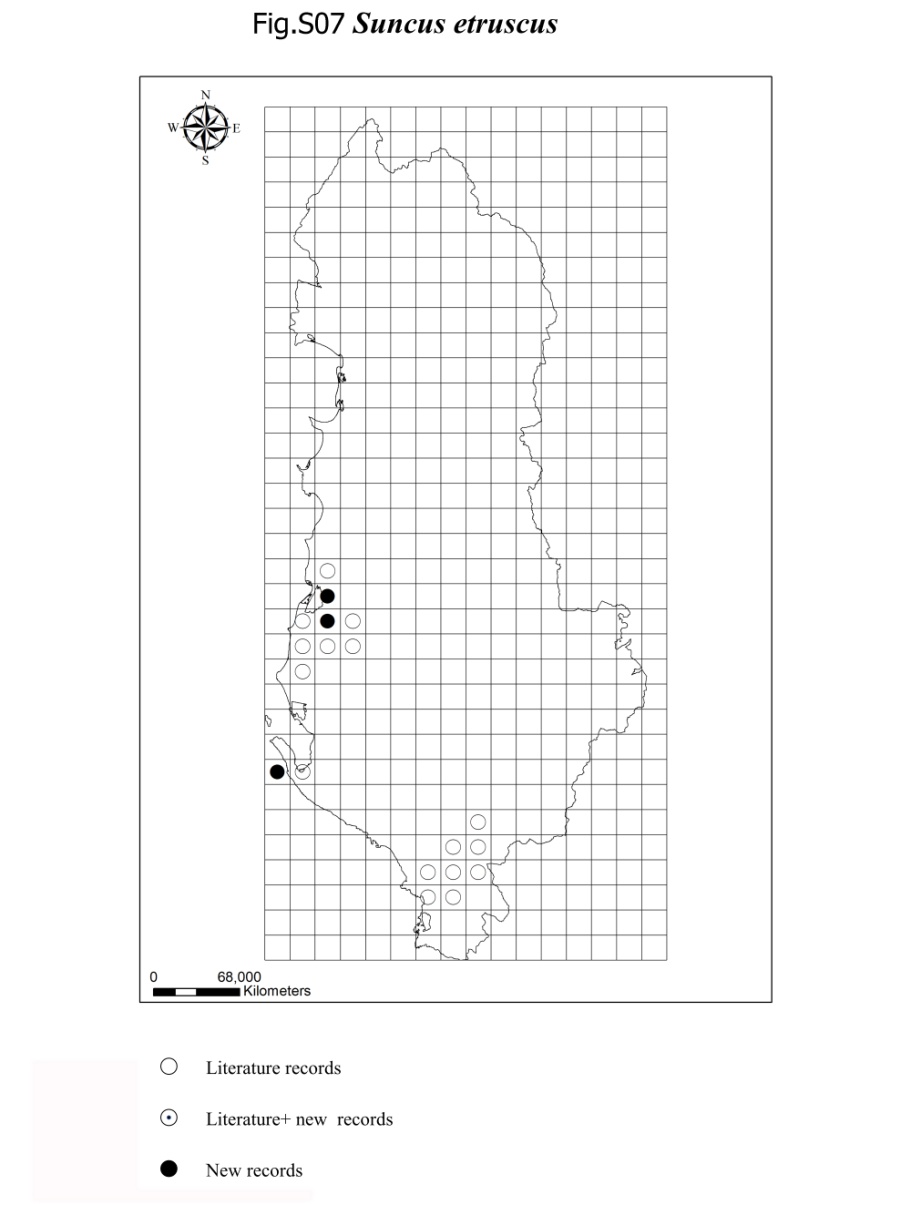

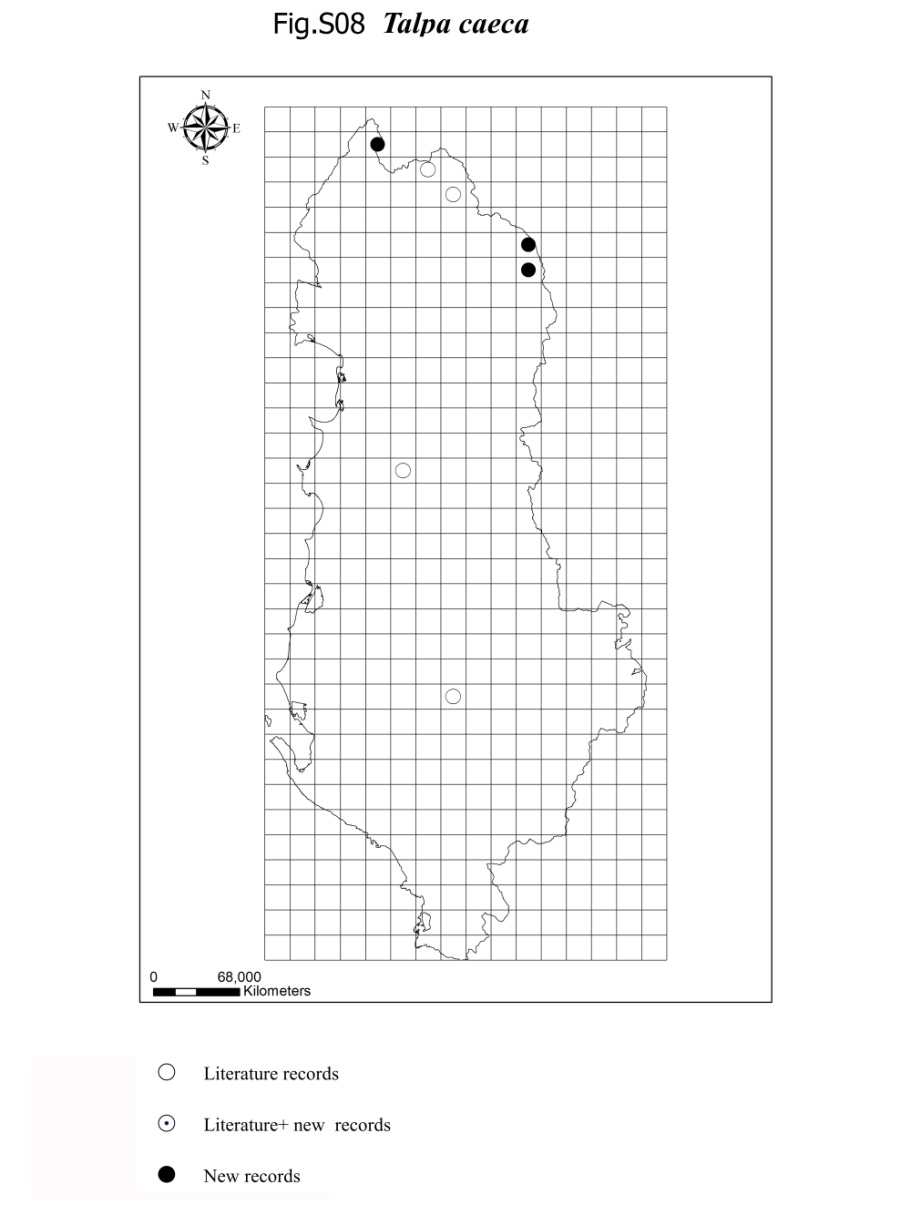

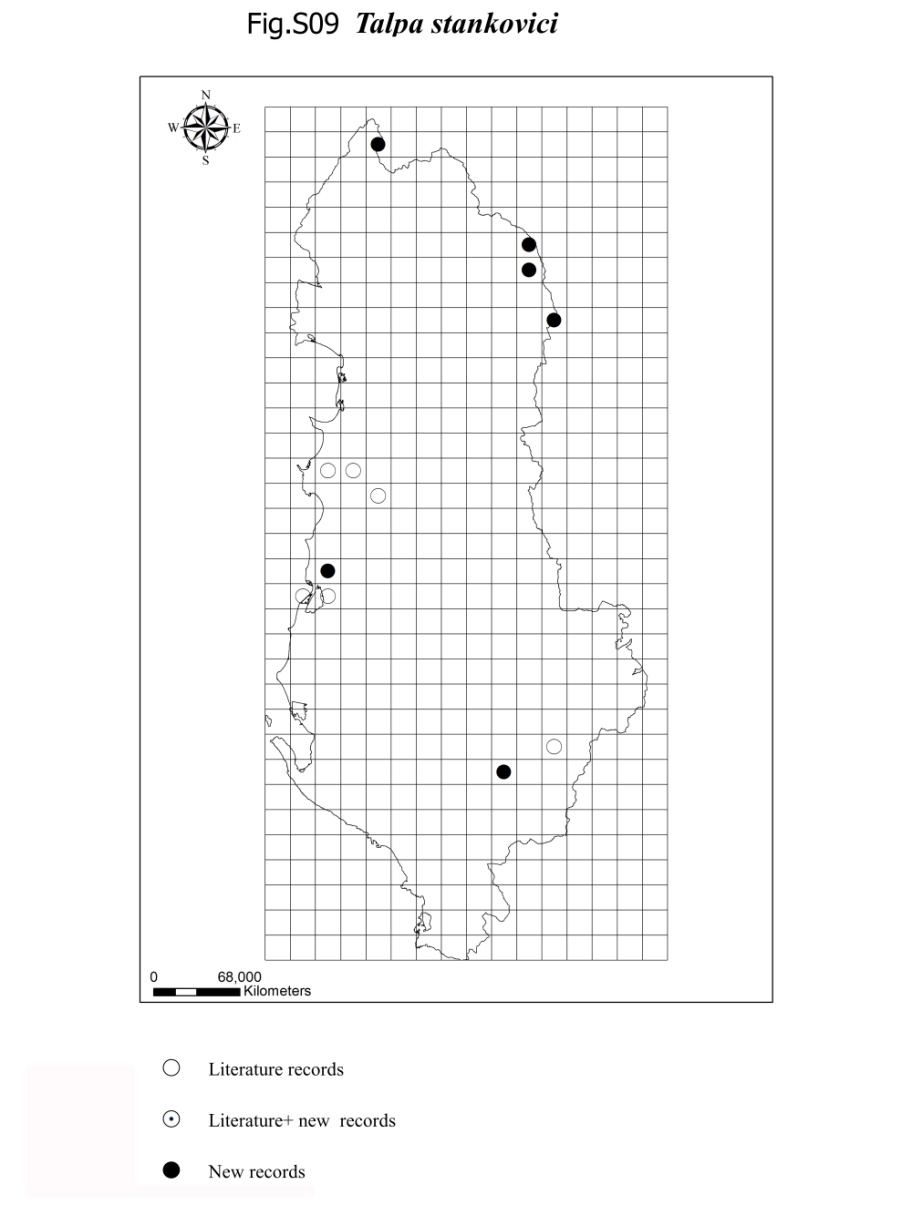


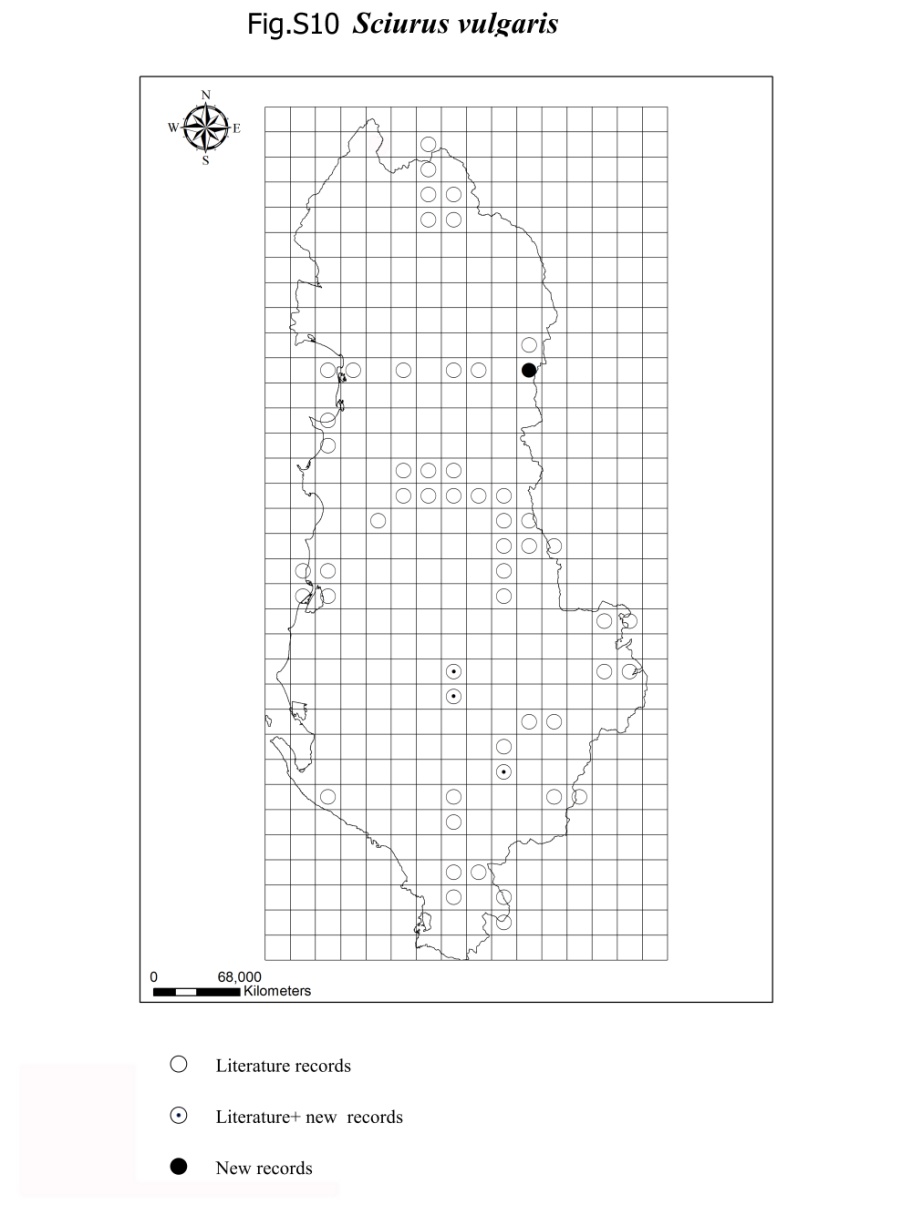

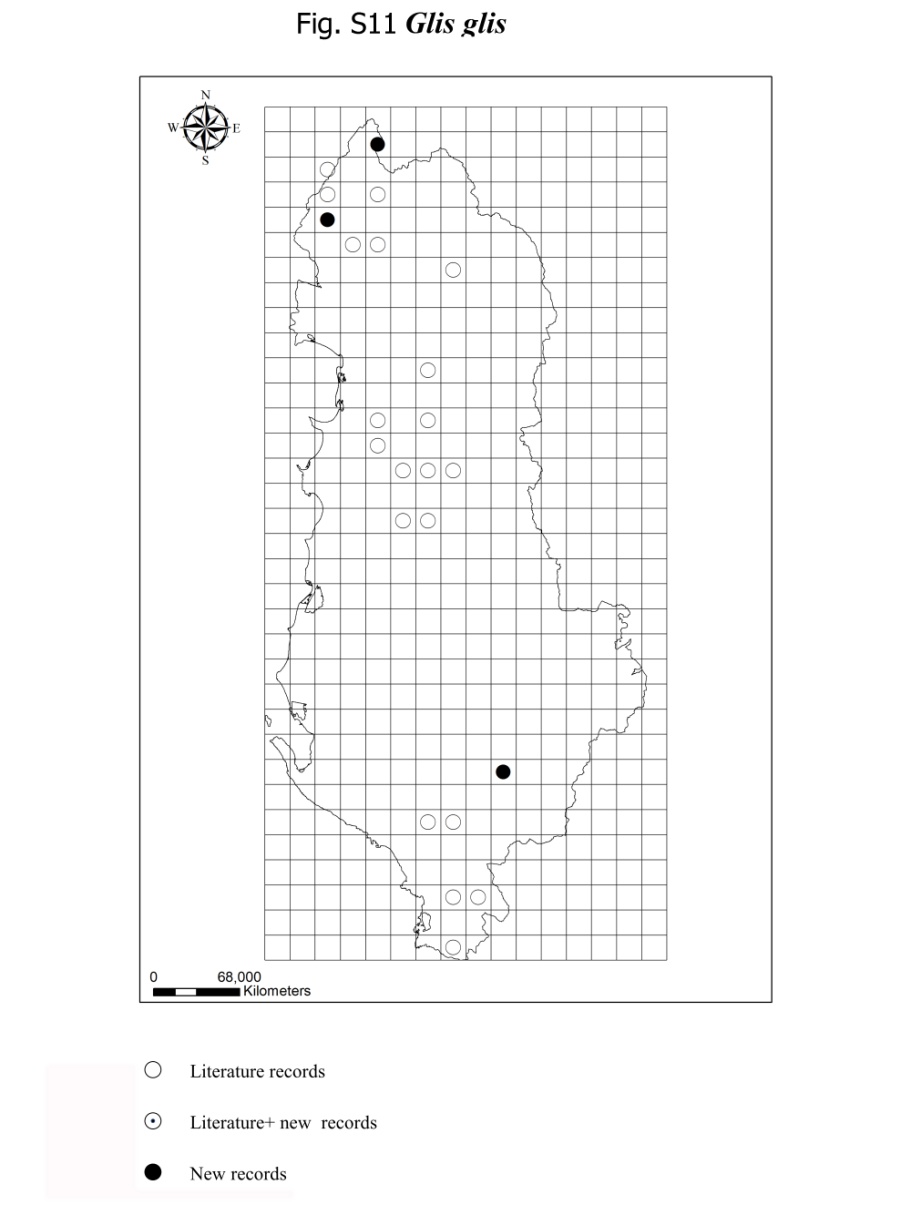

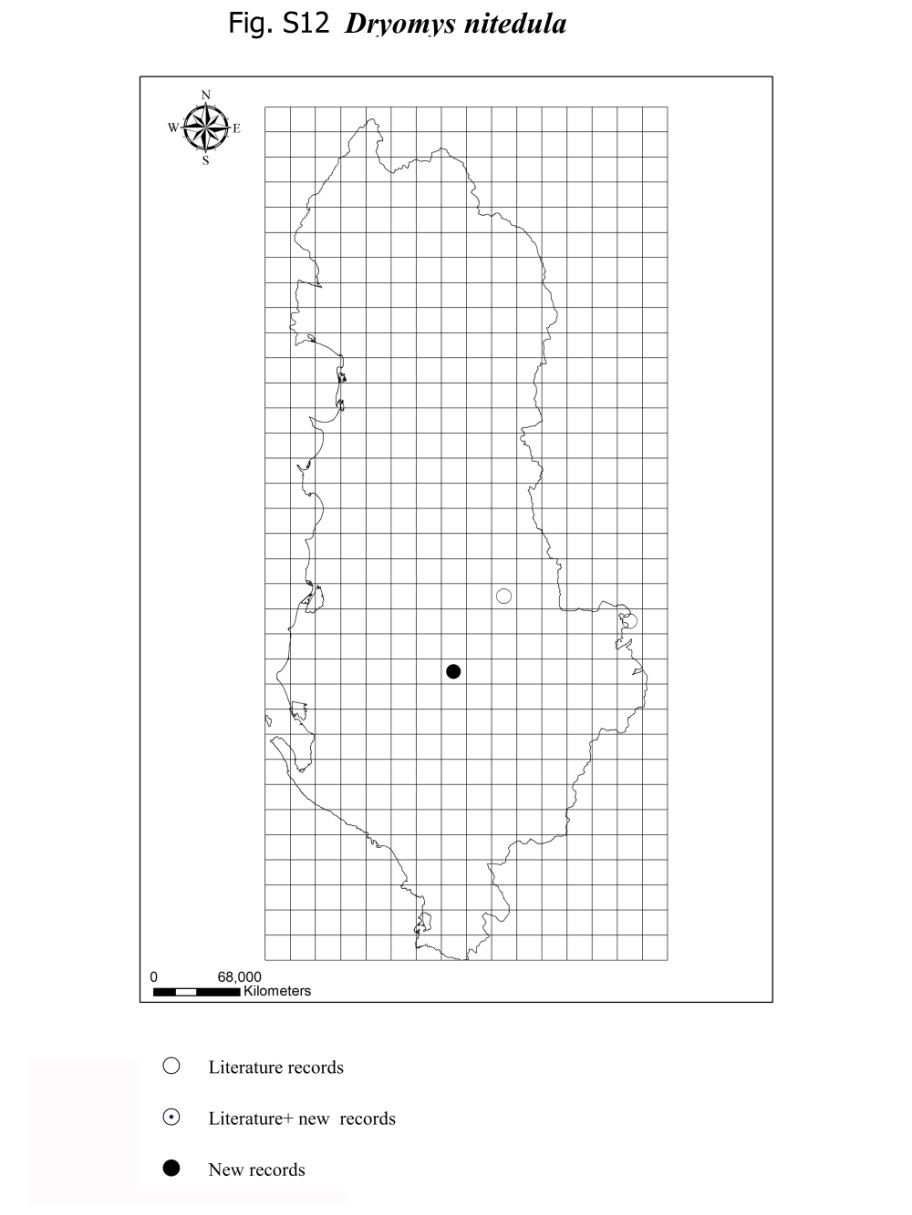


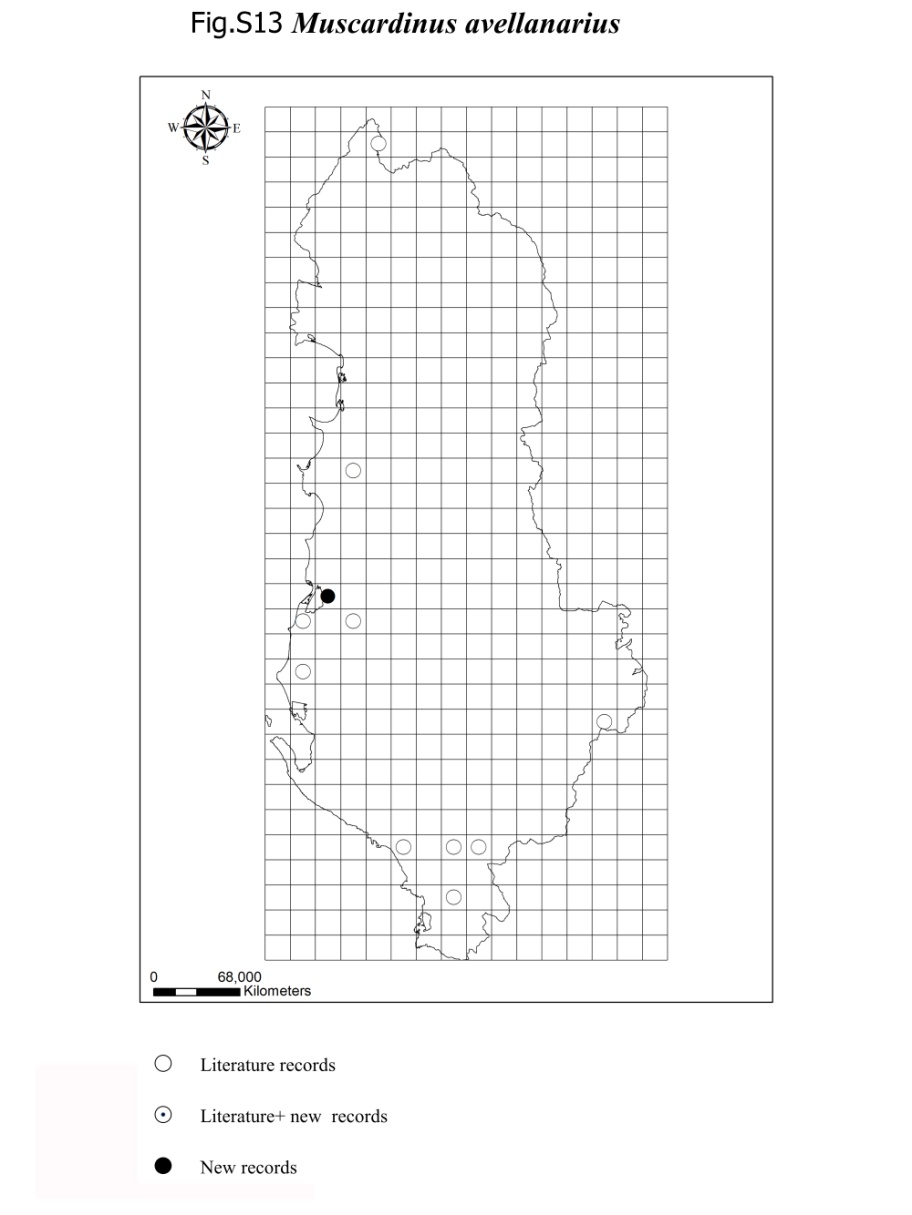

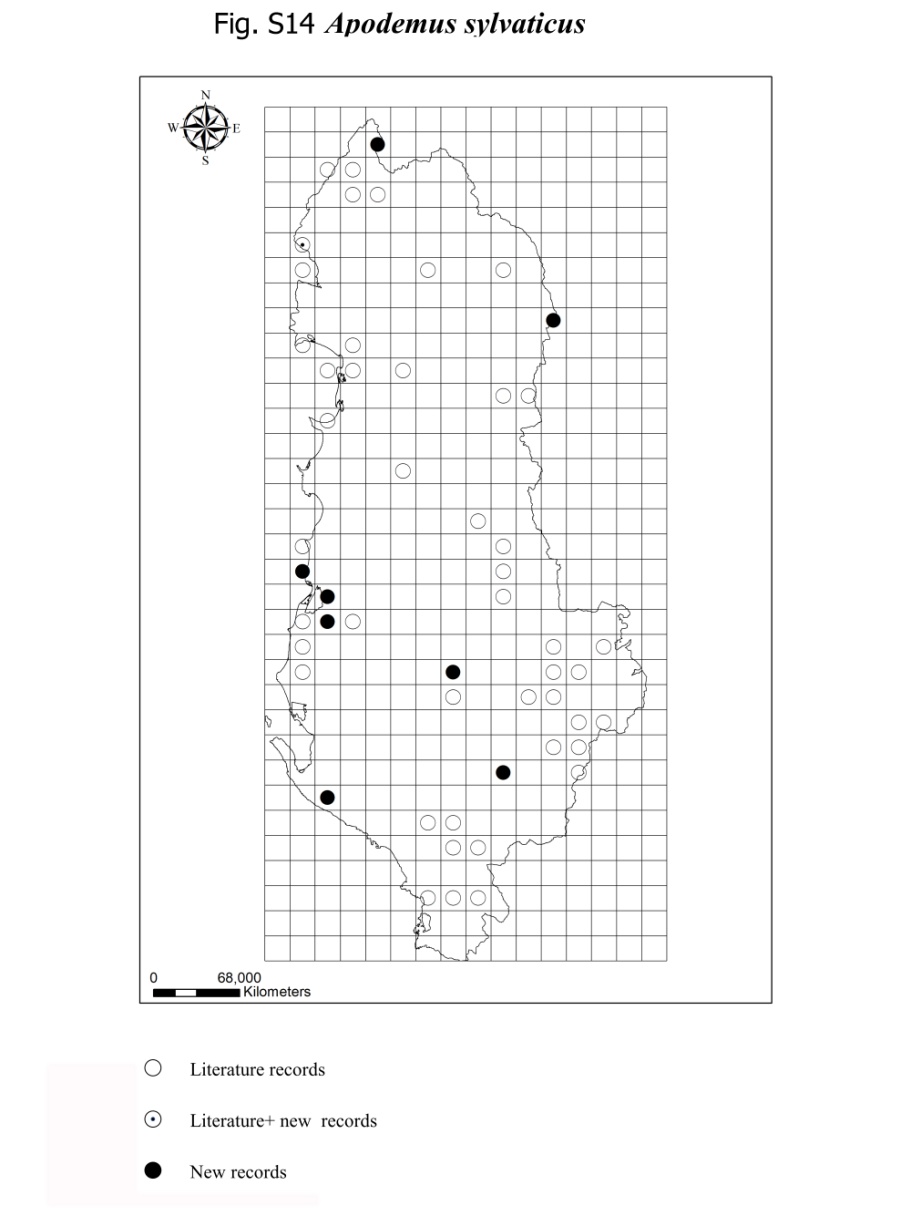

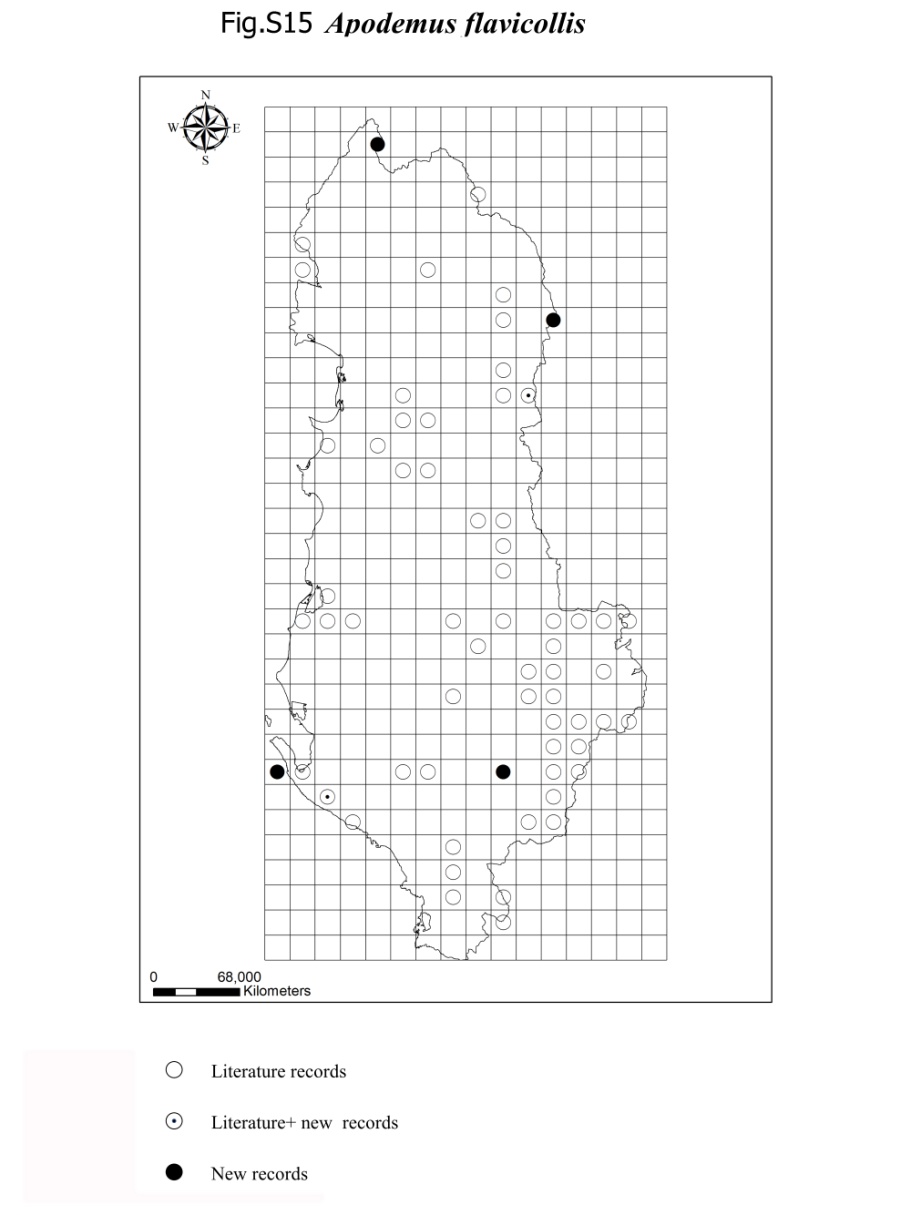


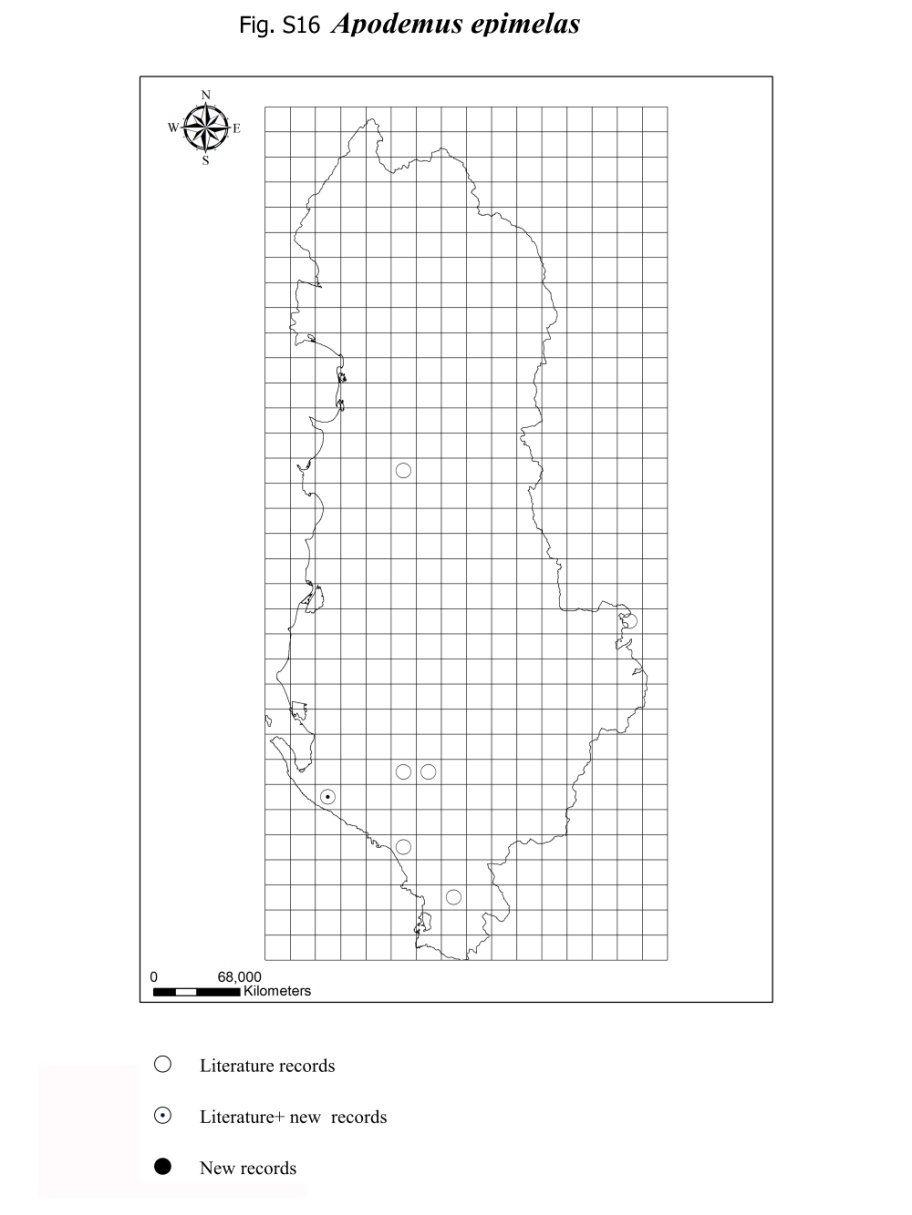

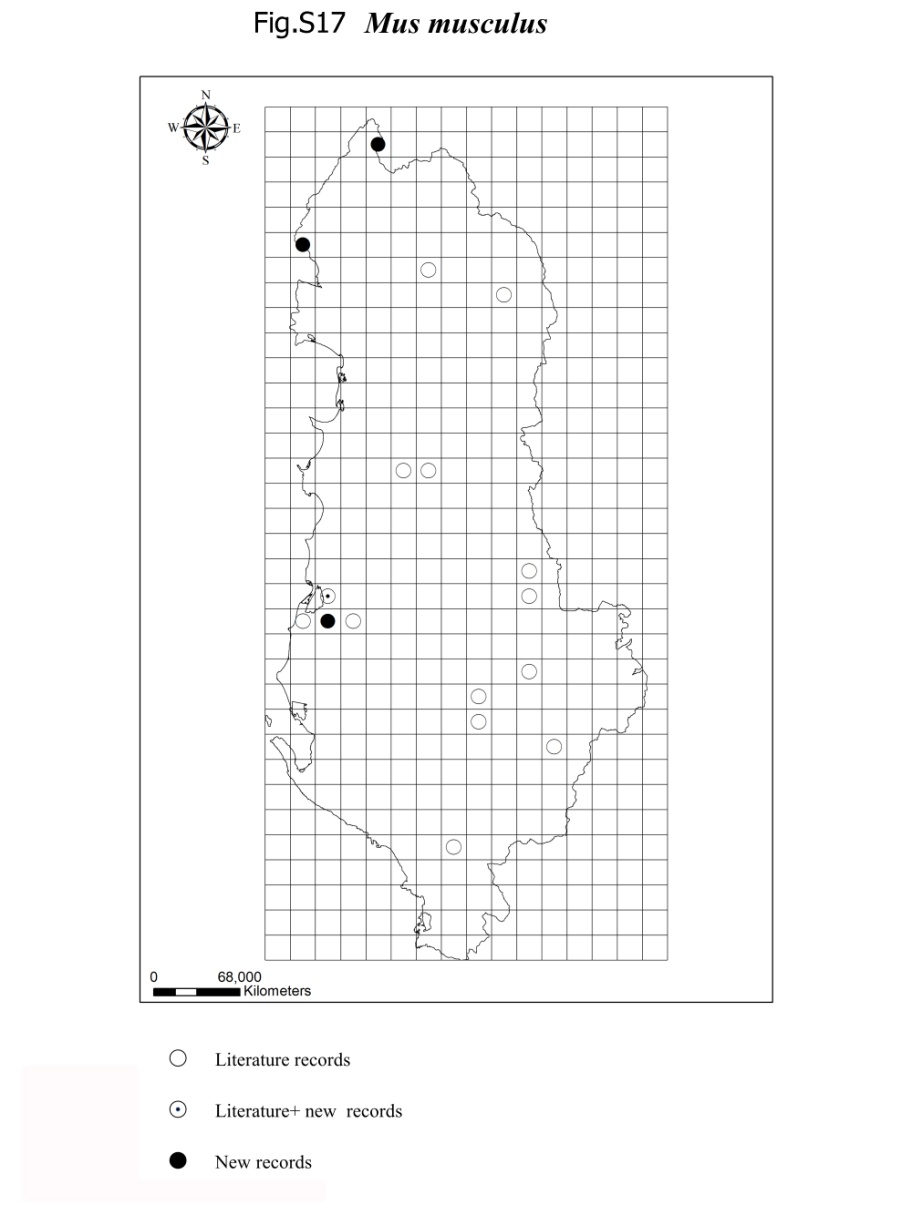

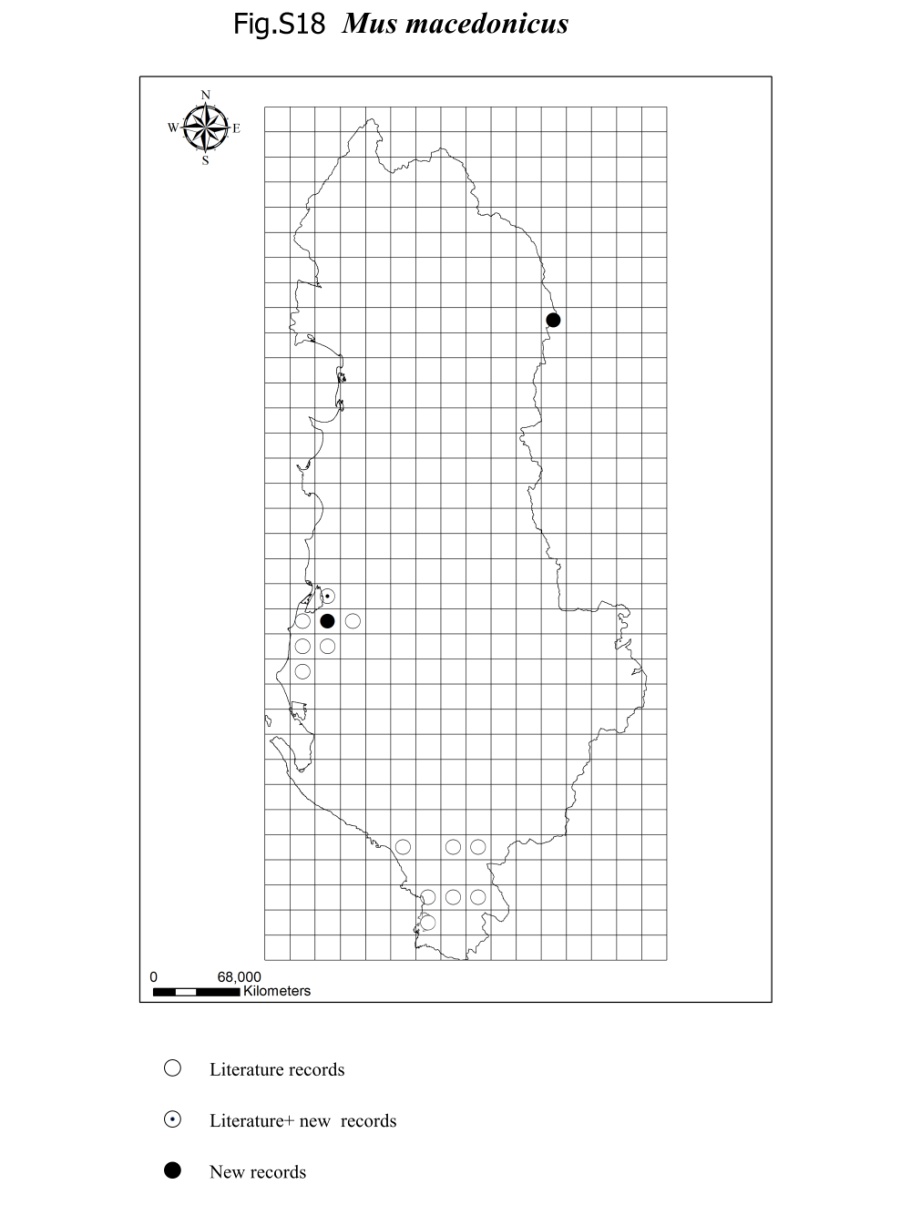


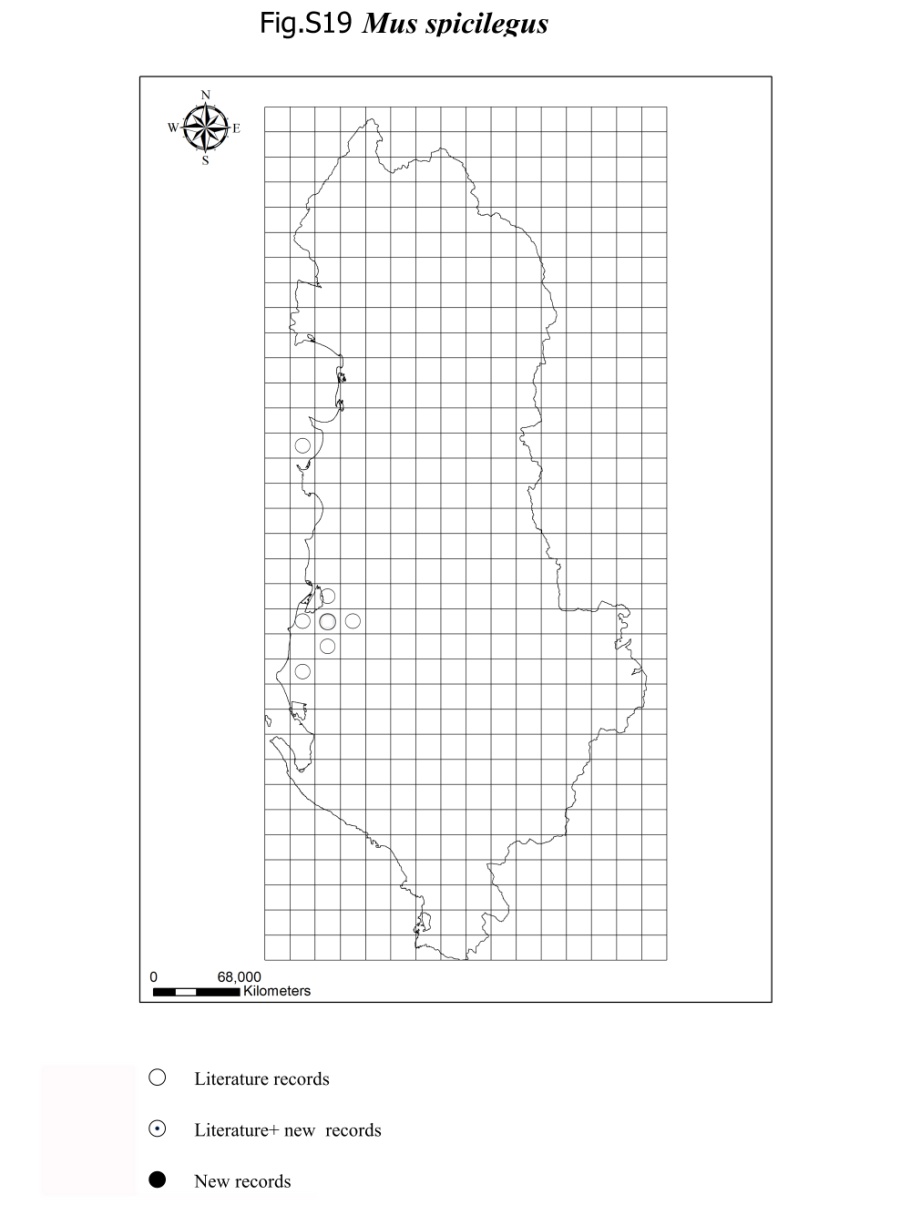

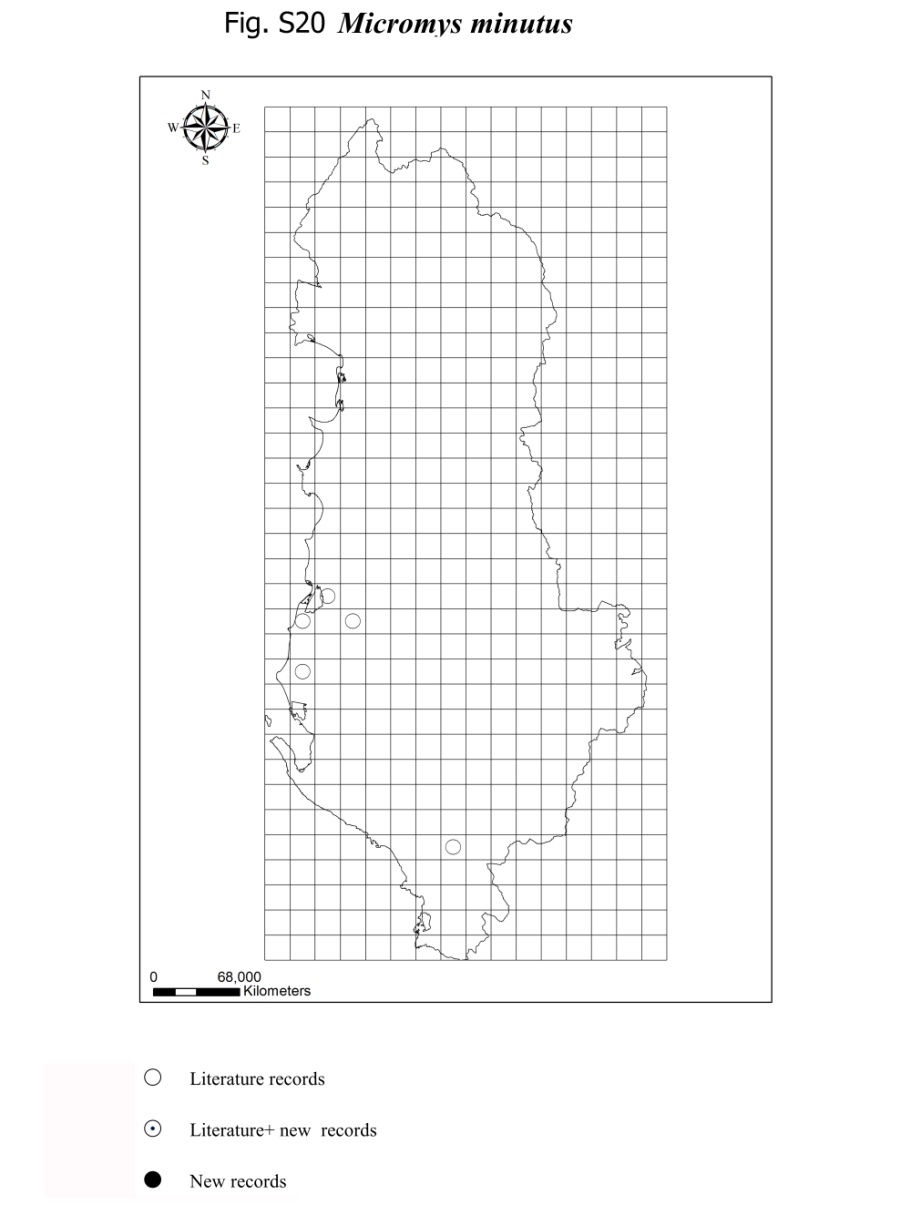

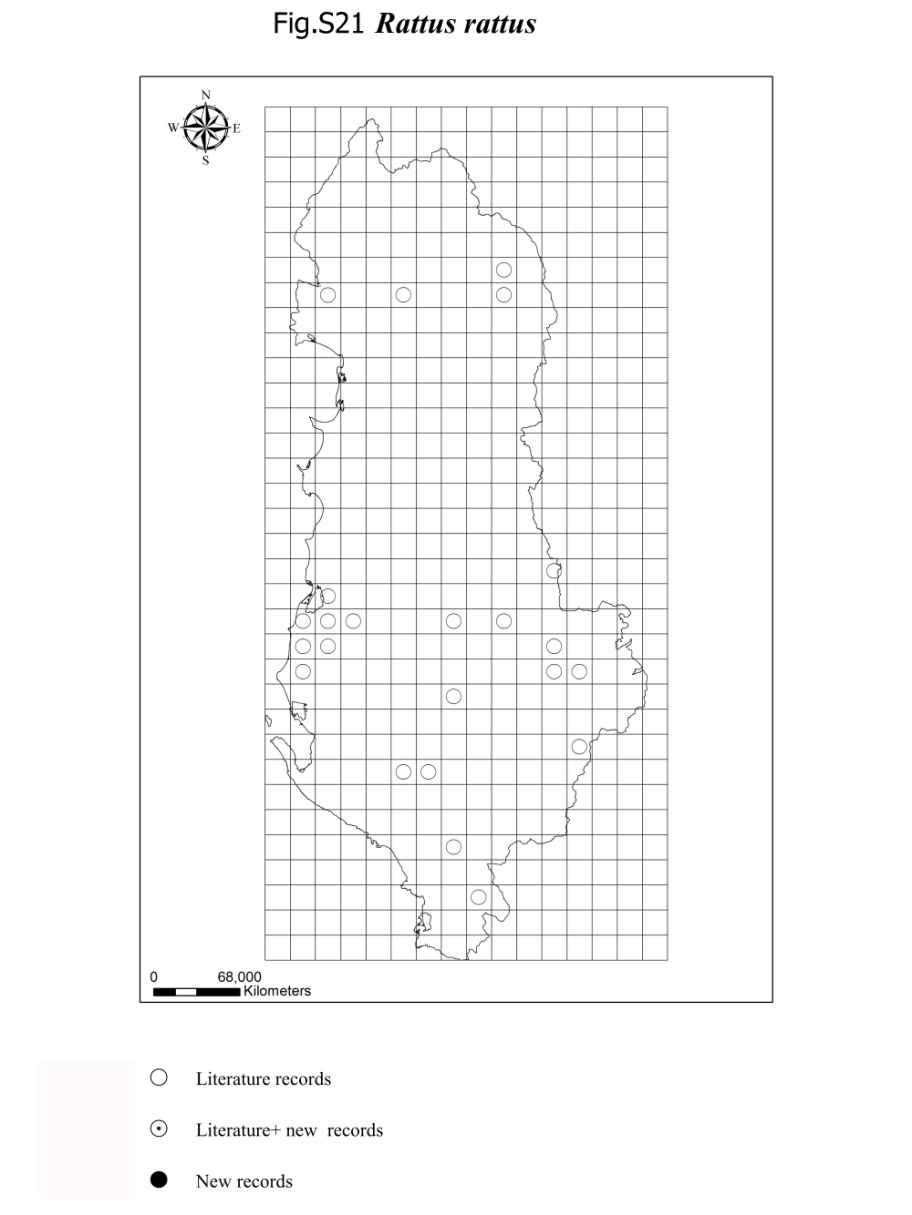


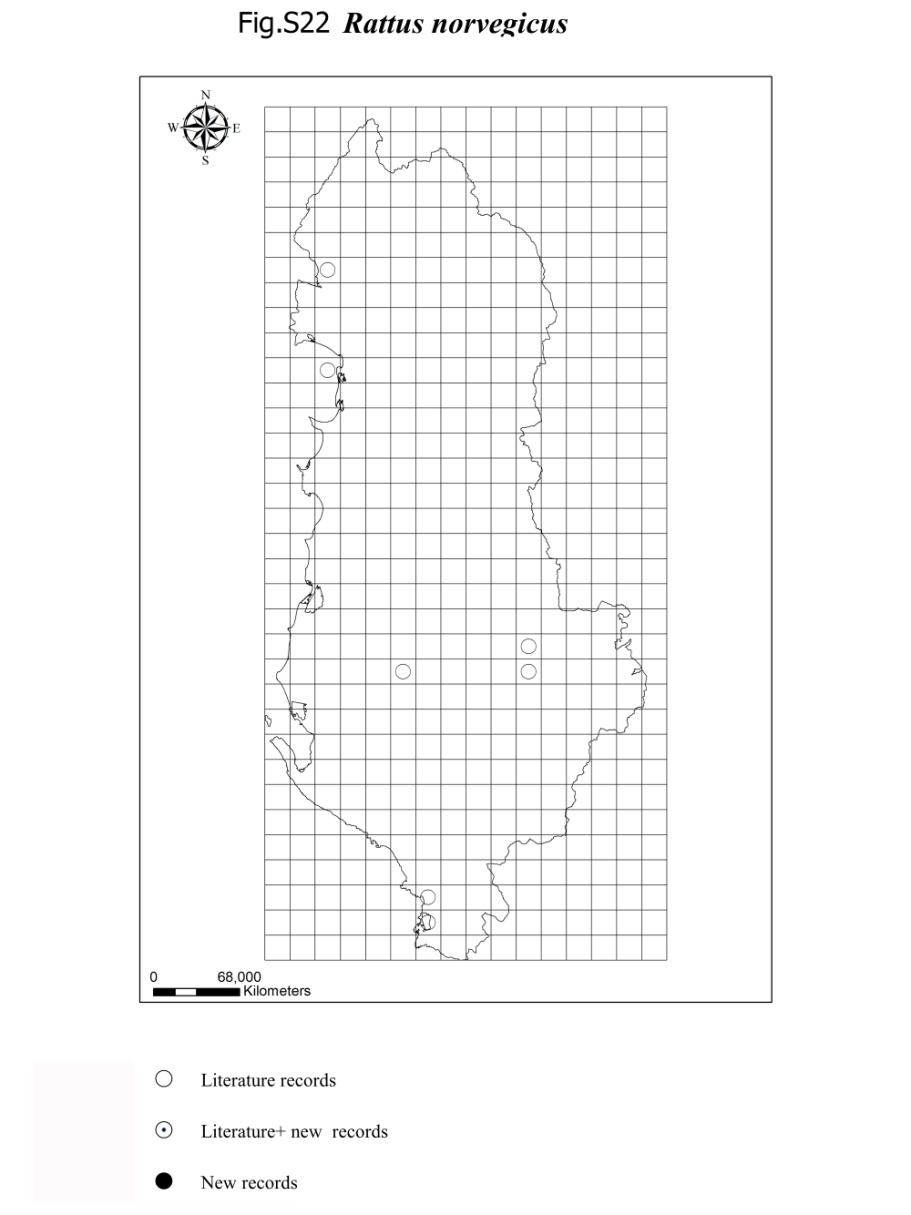

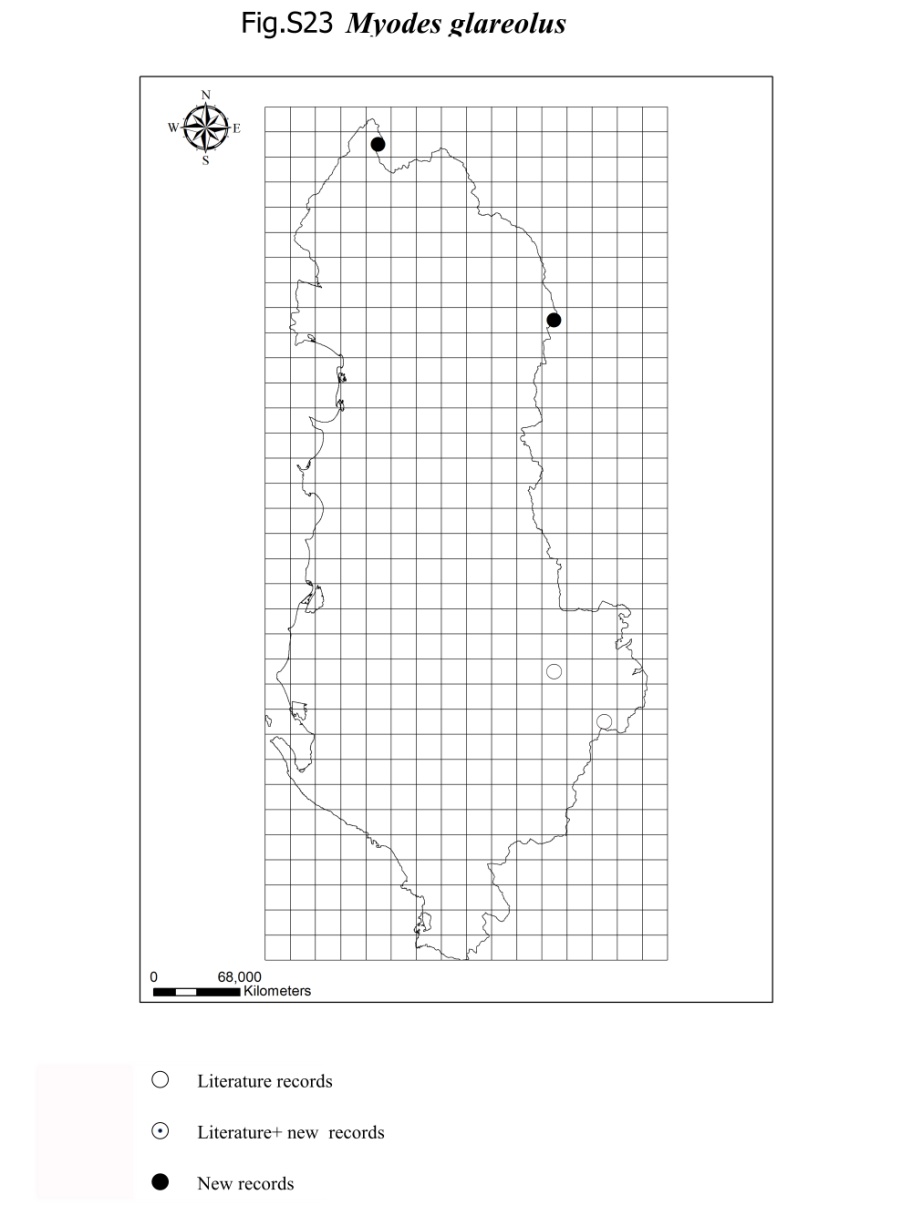

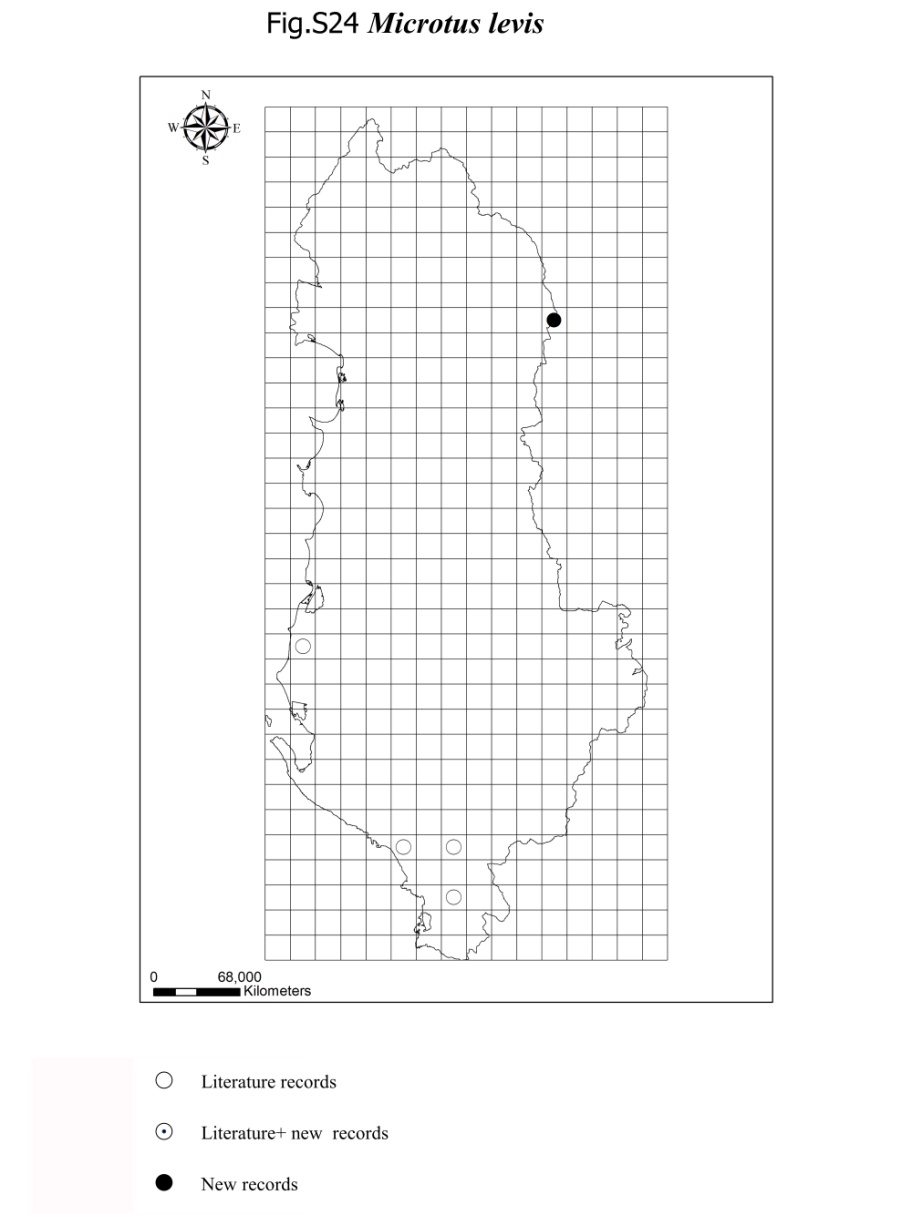


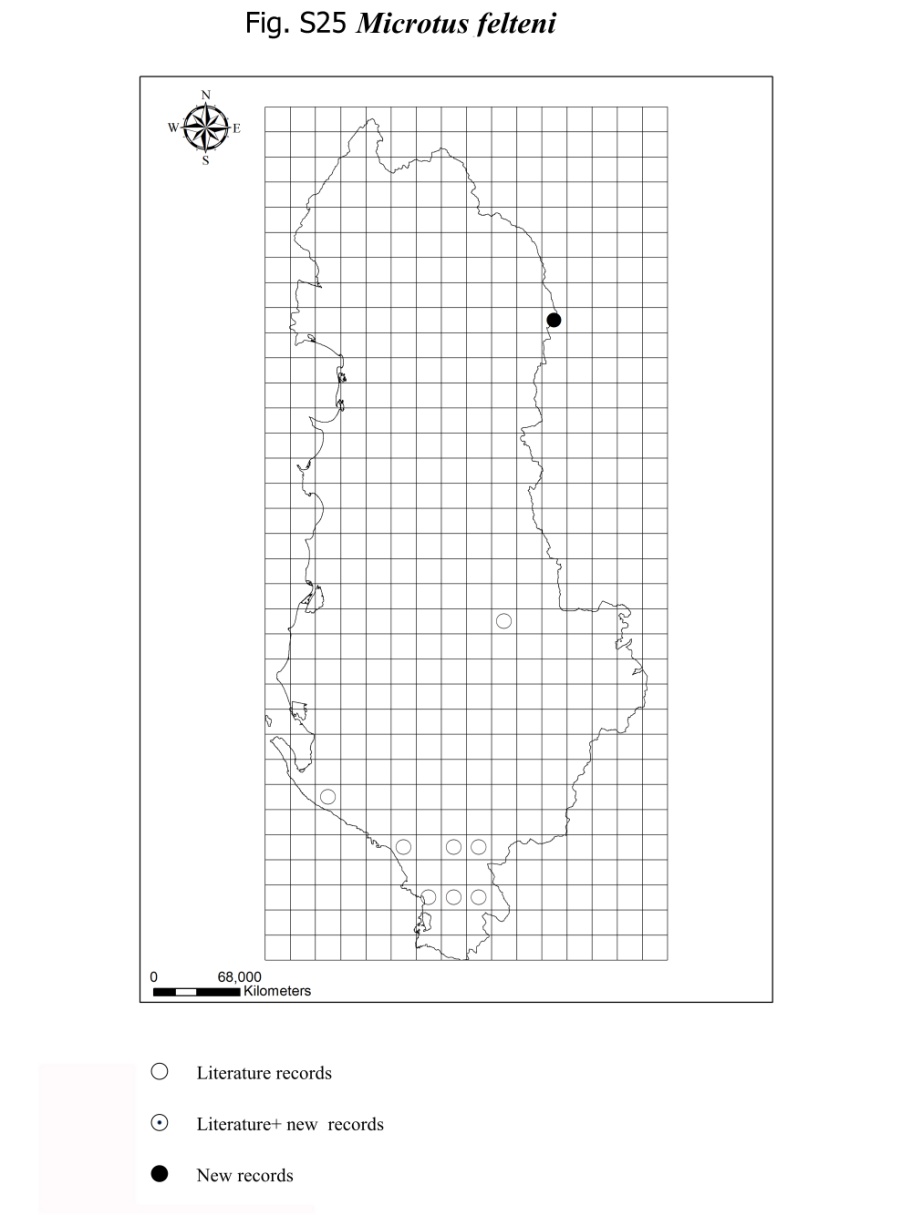

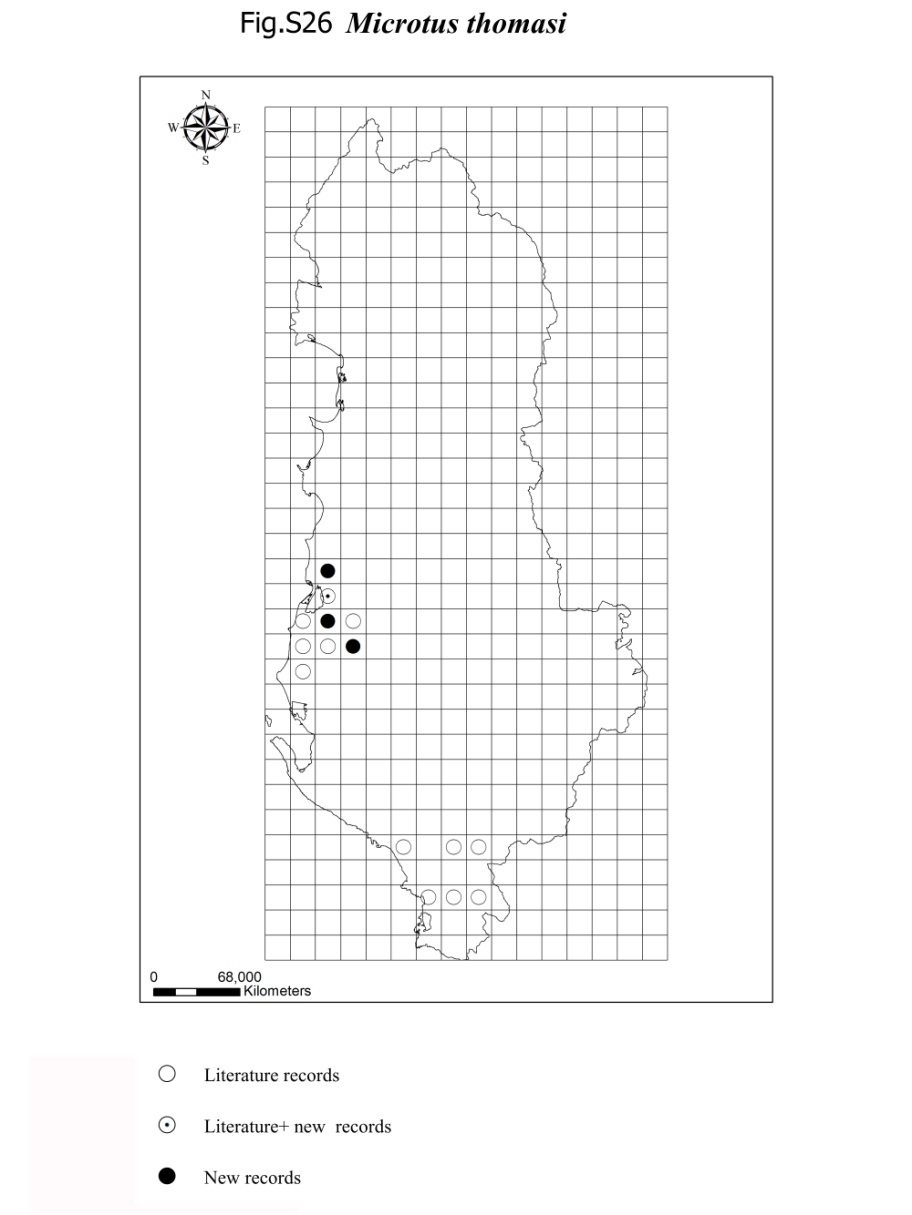

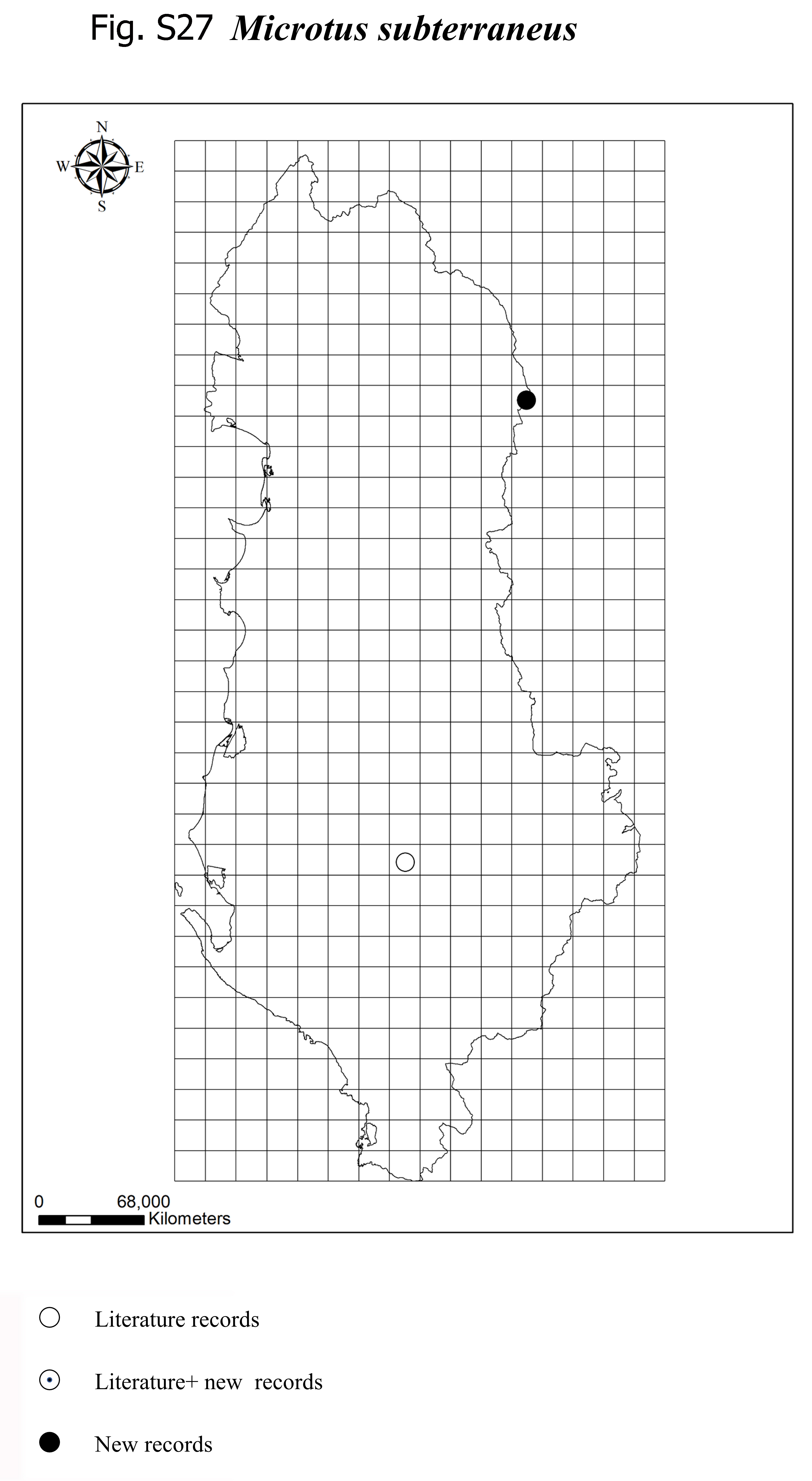


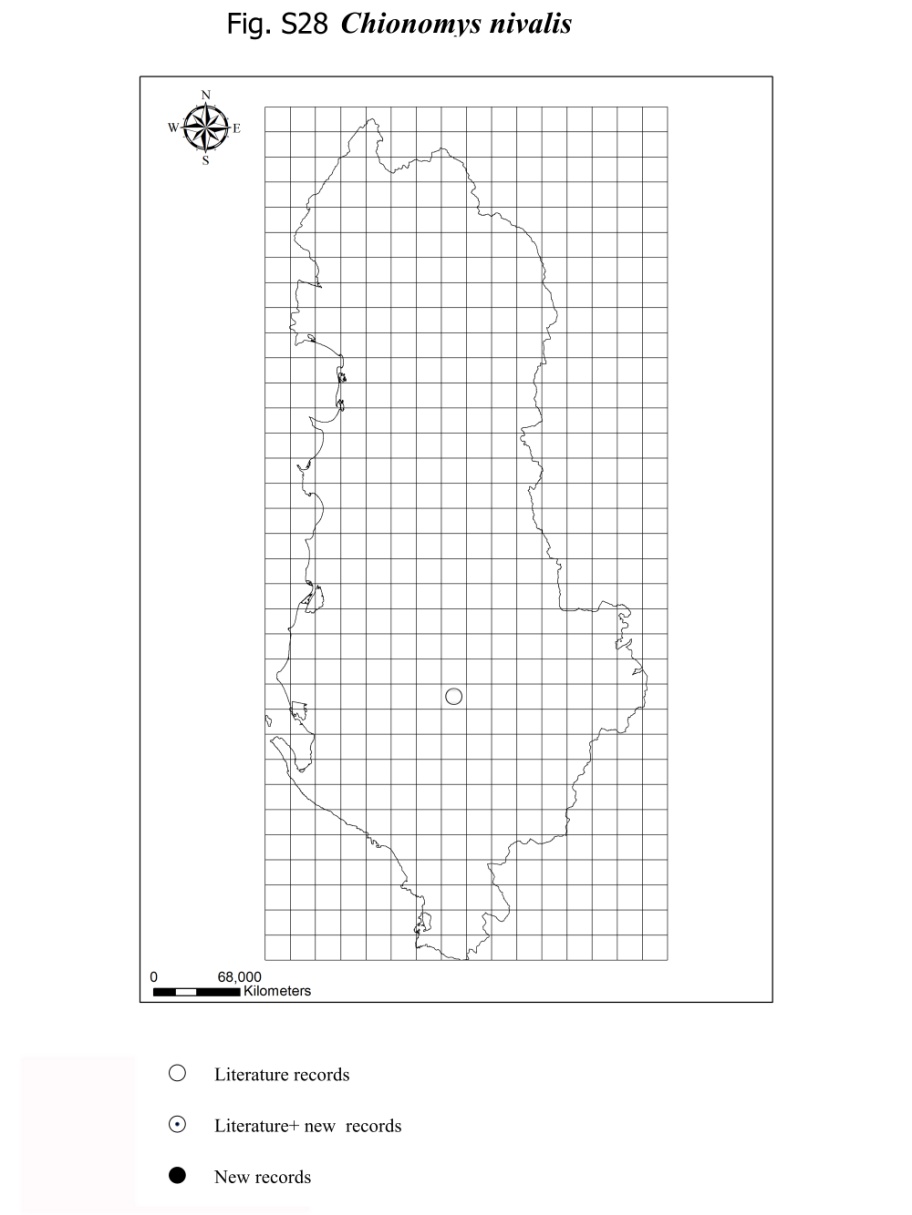

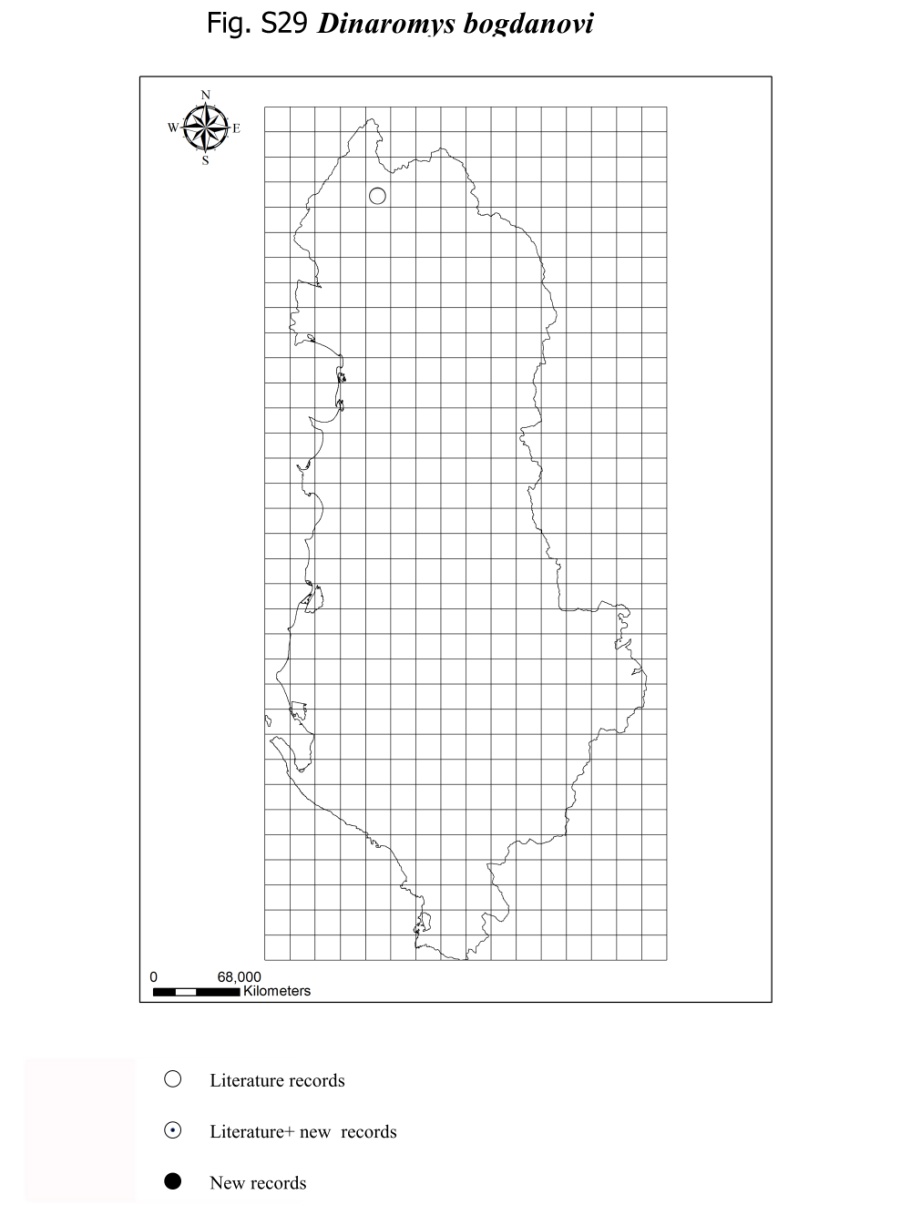

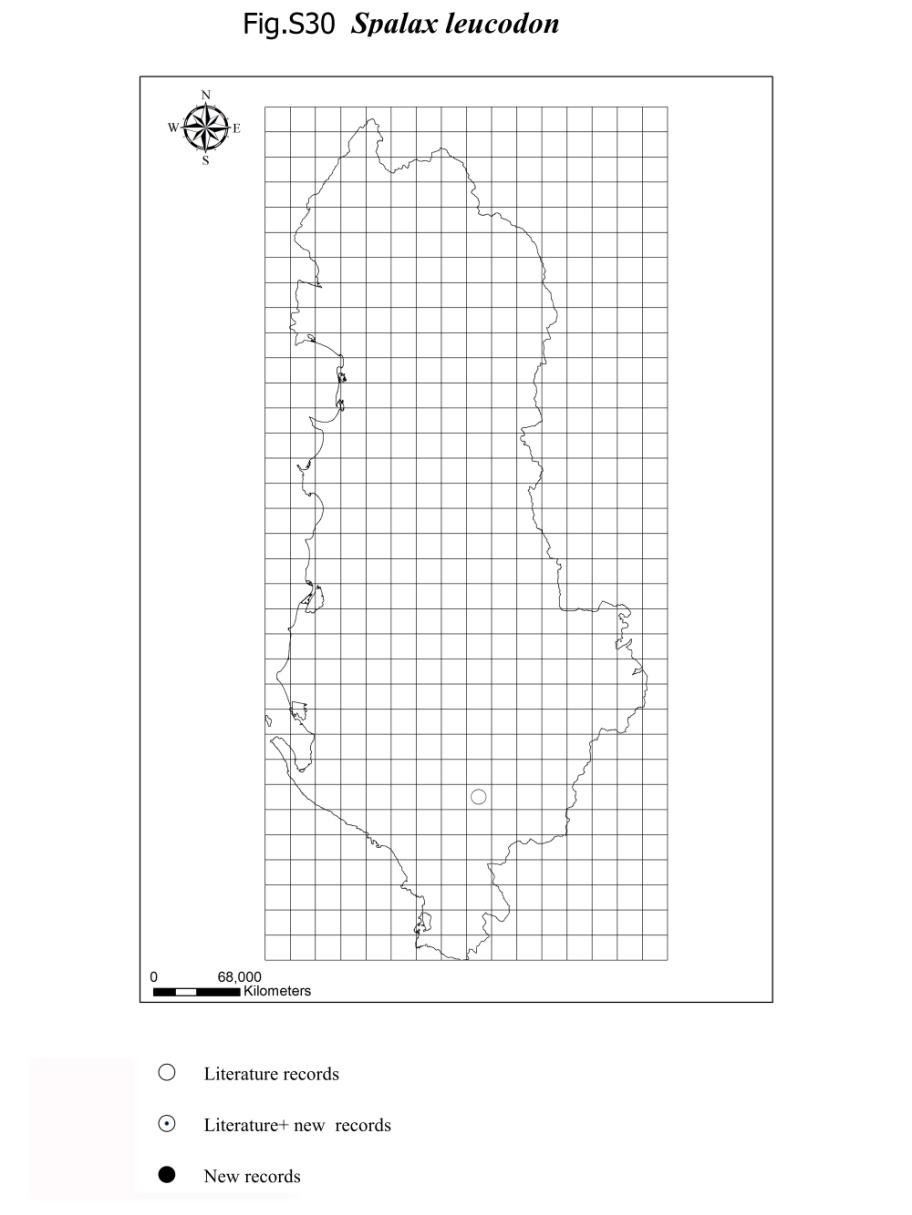


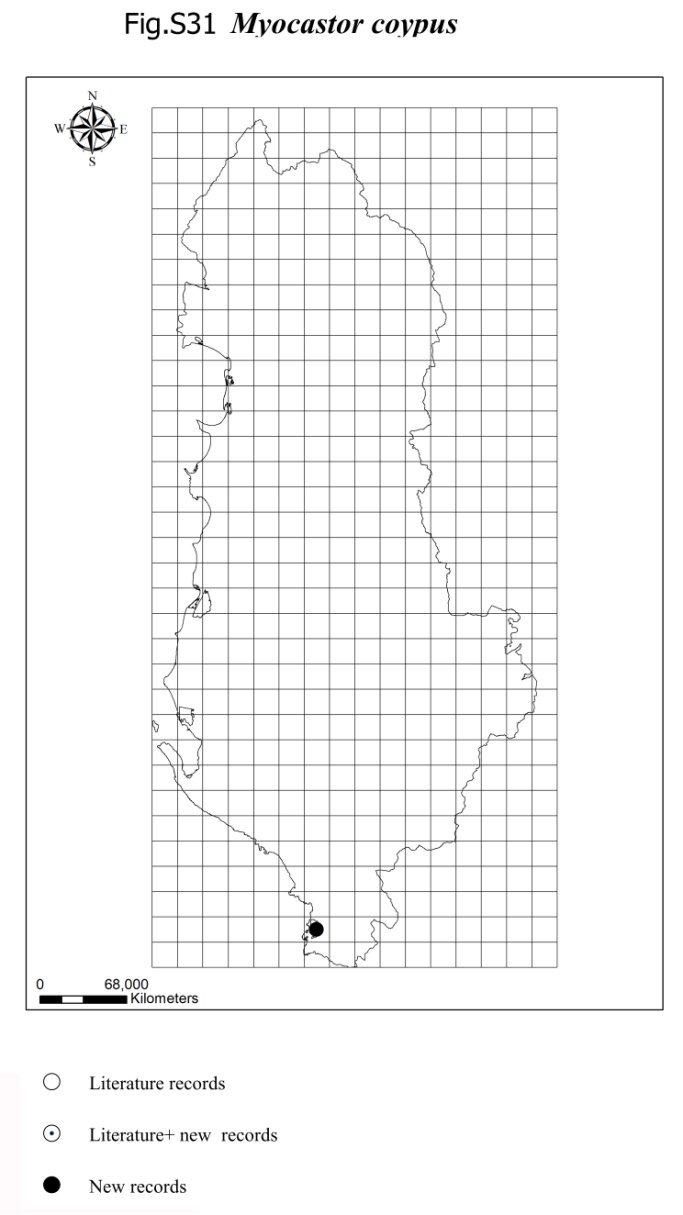

Supplement: Supplementary material 3 [file zookeys-742-127-s003.docx]
